# Supplementary material for: Exploring how infectious diseases control interventions are delivered in low-income urban communities in low- and middle-income countries: A scoping review protocol
Source: PLoS One. 2025 Dec 10;20(12):e0337949. doi: 10.1371/journal.pone.0337949 (PMC12694824; doi:10.1371/journal.pone.0337949)
Supplement: S2 File — (DOCX) [file pone.0337949.s002.docx]

# **Scoping Review: ID Interventions and Low-Income Urban Communities**

**Databases and Strategies:** MEDLINE, Embase, Global Health, and Web of Science

**Initial PRISMA Numbers**

Records Identified from Databases: 7,2724

Duplicate Records Removed (with EndNote 20): 3,295

Duplicate Records Removed (automatically with Covidence): 9

Records Uploaded for Title/Abstract Screening:

| Ovid MEDLINE(R) ALL 1946 to June 13, 2025 | | |
| --- | --- | --- |
| **Date Searched** | 5/17/2025 | |
| **Concept** | **Search String** | **Results** |
| **1. ID Interventions** | ((communicable disease*.ti,ab. OR  infectious disease*.ti,ab. OR  neglected disease*.ti,ab. OR  neglected tropical disease*.ti,ab. OR  vaccine preventable disease*.ti,ab. OR  exp Communicable Diseases/ OR  Neglected Diseases/ OR  Vaccine-Preventable Diseases/ OR  exp Disease Transmission, Infectious/ OR  Communicable Disease Control/) AND  (mass screening.ti,ab. OR  screening.ti,ab. OR  face mask*.ti,ab. OR  "Test Treat and Track".ti,ab. OR  "Test Treat & Track".ti,ab. OR  "Test and Treat".ti,ab. OR  "Test & Treat".ti,ab. OR  Mandatory Testing/ OR  exp Mass Screening/)) OR  (bednet* OR  bed net* OR  condom* OR  contact tracing OR  deworming OR  immuniz* OR  immunis* OR  inoculat* OR  mass drug administration OR  preventive chemotherap* OR  vaccin* OR  insecticide treated net* OR  mosquito net OR  mosquito nets OR  "Surgery, Antibiotics, Facial cleanliness and Environmental improvements" OR  "Surgery, Antibiotics, Facial cleanliness & Environmental improvements" OR  (SAFE ADJ4 (World Health Organization OR WHO))).ti,ab,kf. OR  exp Anti-Infective Agents/ OR  Condoms/ OR  Contact Tracing/ OR  Disease Eradication/ OR  Insecticide-Treated Bednets/ OR  Mosquito Nets/ OR  Immunization/ OR  Immunization Programs/ OR  Mass Drug Administration/ OR  Mass Vaccination/ OR  Post-Exposure Prophylaxis/ OR  Pre-Exposure Prophylaxis/ OR  Vaccination/ OR  exp Vaccines/ | 2,701,209 |
| **2. Low-Income and Urban** | ((cities OR  city OR  municipalit* OR  peri urban* OR  periurban* OR  township* OR  urban*)  AND  ("aashwa'i" OR  arrabal OR  asentamiento* OR  barraca OR  brarek OR  campamento* OR  ciudad perdida OR  comuna OR  comunas OR  habitat precaire OR  habitat spontane OR  homeless camp OR  homeless encampment OR  hovel* OR  human settlement development OR  illegal settlement* OR  illegal shack* OR  imijondolo* OR  immigrant camp OR  immigrant camps OR  immigrant settlement OR  immigrant settlements OR  impoverish* OR  informal encampment* OR  informal housing OR  informal settlement* OR  informal shack* OR  irregular settlement* OR  irregular shack* OR  katra OR  kijiji OR  loteamento OR  low income OR  low socioeconomic OR  migrant camp OR  migrant camps OR  migrant settlement* OR  poblacion callampa OR  poor OR  poverty OR  precario OR  public squalor OR  quartier irregulier OR  refugee camp OR  refugee camps OR  refugee settlement* OR  rundown settlement* OR  shack dweller* OR  shackdweller* OR  shack settlement* OR  shack town* OR  shanties OR  shanty OR  shantyhouse* OR  skid row OR  squalid accommodation OR  squalid housing OR  squatter camp* OR  street dweller* OR  street encampment* OR  tanake OR  tenement district* OR  tenement hous* OR  tent city OR  tent cities OR  tugurio* OR  umjondolo)).ti,ab,kf. OR  (ahata OR  barriada* OR  barrio baja OR  barrio pobre OR  basti* OR  bidon OR  bidons OR  bidonville* OR  bustee* OR  cantegril* OR  cardboard city OR  chacarita OR  chawl OR  chawls OR  cite soleil OR  colonia popular OR  comunidade OR  conventillos OR  deponija OR  dharavi OR  edina achouaia OR  elendsviertel OR  favela* OR  foundouks OR  gecekondu* OR  ghetto OR  ghettos OR  informal city OR  informal cities OR  informal urban settlement* OR  jhopadpatti OR  jihuggi OR  kampung OR  kartonsko naselje OR  katchi abadi* OR  khayelitsha OR  kibera OR  lahbach OR  mabanda OR  masseque* OR  morro OR  mudun safi OR  orangi town OR  pelli gewal OR  pueblos joven* OR  rundown neighborhood* OR  rundown neighbourhood* OR  shantytown* OR  slum OR  slums OR  squatter cit* OR  taudi OR  taudis OR  truschobi OR  urban blight OR  urban encampment* OR  urban poor OR  urban slum* OR  urban squat* OR  villa miseria OR  watta).ti,ab,kf. OR  ((Poverty Areas/ OR  Poverty/ OR  Child Poverty/ OR  Ill-Housed Persons/)  AND  (Urban Population/ OR  Urban Health/ OR  Urbanization/ OR  Cities/)) | 48,142 |
| **3. LIMCs** | ("afghanistan" OR "albania" OR "algeria" OR "american samoa" OR "angola" OR "antigua and barbuda" OR "antigua" OR "barbuda" OR "argentina" OR "armenia" OR "armenian" OR "aruba" OR "azerbaijan" OR "bahrain" OR "bangladesh" OR "barbados" OR "republic of belarus" OR "belarus" OR "byelarus" OR "belorussia" OR "byelorussian" OR "belize" OR "british honduras" OR "benin" OR "dahomey" OR "bhutan" OR "bolivia" OR "bosnia and herzegovina" OR "bosnia" OR "herzegovina" OR "botswana" OR "bechuanaland" OR "brazil" OR "brasil" OR "bulgaria" OR "burkina faso" OR "burkina fasso" OR "upper volta" OR "burundi" OR "urundi" OR "cabo verde" OR "cape verde" OR "cambodia" OR "kampuchea" OR "khmer republic" OR "cameroon" OR "cameron" OR "cameroun" OR "central african republic" OR "ubangi shari" OR "chad" OR "chile" OR "china" OR "colombia" OR "comoros" OR "comoro islands" OR "iles comores" OR "mayotte" OR "democratic republic of the congo" OR "democratic republic congo" OR "congo" OR "zaire" OR "costa rica" OR "cote d’ivoire" OR "cote d’ ivoire" OR "cote divoire" OR "cote d ivoire" OR "ivory coast" OR "croatia" OR "cuba" OR "cyprus" OR "czech republic" OR "czechoslovakia" OR "djibouti" OR "french somaliland" OR "dominica" OR "dominican republic" OR "ecuador" OR "egypt" OR "united arab republic" OR "el salvador" OR "equatorial guinea" OR "spanish guinea" OR "eritrea" OR "estonia" OR "eswatini" OR "swaziland" OR "ethiopia" OR "fiji" OR "gabon" OR "gabonese republic" OR "gambia" OR "georgia (republic)" OR "georgian" OR "ghana" OR "gold coast" OR "gibraltar" OR "greece" OR "grenada" OR "guam" OR "guatemala" OR "guinea" OR "guinea bissau" OR "guyana" OR "british guiana" OR "haiti" OR "hispaniola" OR "honduras" OR "hungary" OR "india" OR "indonesia" OR "timor" OR "iran" OR "iraq" OR "isle of man" OR "jamaica" OR "jordan" OR "kazakhstan" OR "kazakh" OR "kenya" OR "democratic people’s republic of korea" OR "republic of korea" OR "north korea" OR "south korea" OR "korea" OR "kosovo" OR "kyrgyzstan" OR "kirghizia" OR "kirgizstan" OR "kyrgyz republic" OR "kirghiz" OR "laos" OR "lao pdr" OR "lao people's democratic republic" OR "latvia" OR "lebanon" OR "lebanese republic" OR "lesotho" OR "basutoland" OR "liberia" OR "libya" OR "libyan arab jamahiriya" OR "lithuania" OR "macau" OR "macao" OR "republic of north macedonia" OR "macedonia" OR "madagascar" OR "malagasy republic" OR "malawi" OR "nyasaland" OR "malaysia" OR "malay federation" OR "malaya federation" OR "maldives" OR "indian ocean islands" OR "mali" OR "malta" OR "micronesia" OR "federated states of micronesia" OR "kiribati" OR "marshall islands" OR "nauru" OR "northern mariana islands" OR "palau" OR "tuvalu" OR "mauritania" OR "mauritius" OR "mexico" OR "moldova" OR "moldovian" OR "mongolia" OR "montenegro" OR "morocco" OR "ifni" OR "mozambique" OR "portuguese east africa" OR "myanmar" OR "burma" OR "namibia" OR "nepal" OR "netherlands antilles" OR "nicaragua" OR "niger" OR "nigeria" OR "oman" OR "muscat" OR "pakistan" OR "panama" OR "papua new guinea" OR "new guinea" OR "paraguay" OR "peru" OR "philippines" OR "philipines" OR "phillipines" OR "phillippines" OR "poland" OR "polish people's republic" OR "portugal" OR "portuguese republic" OR "puerto rico" OR "romania" OR "russia" OR "russian federation" OR "ussr" OR "soviet union" OR "union of soviet socialist republics" OR "rwanda" OR "ruanda" OR "samoa" OR "pacific islands" OR "polynesia" OR "samoan islands" OR "navigator island" OR "navigator islands" OR "sao tome and principe" OR "saudi arabia" OR "senegal" OR "serbia" OR "seychelles" OR "sierra leone" OR "slovakia" OR "slovak republic" OR "slovenia" OR "melanesia" OR "solomon island" OR "solomon islands" OR "norfolk island" OR "norfolk islands" OR "somalia" OR "south africa" OR "south sudan" OR "sri lanka" OR "ceylon" OR "saint kitts and nevis" OR "st. kitts and nevis" OR "saint lucia" OR "st. lucia" OR "saint vincent and the grenadines" OR "saint vincent" OR "st. vincent" OR "grenadines" OR "sudan" OR "suriname" OR "surinam" OR "dutch guiana" OR "netherlands guiana" OR "syria" OR "syrian arab republic" OR "tajikistan" OR "tadjikistan" OR "tadzhikistan" OR "tadzhik" OR "tanzania" OR "tanganyika" OR "thailand" OR "siam" OR "timor leste" OR "east timor" OR "togo" OR "togolese republic" OR "tonga" OR "trinidad and tobago" OR "trinidad" OR "tobago" OR "tunisia" OR "turkey" OR "turkiye" OR "turkmenistan" OR "turkmen" OR "uganda" OR "ukraine" OR "uruguay" OR "uzbekistan" OR "uzbek" OR "vanuatu" OR "new hebrides" OR "venezuela" OR "vietnam" OR "viet nam" OR "middle east" OR "west bank" OR "gaza" OR "palestine" OR "yemen" OR "yugoslavia" OR "zambia" OR "zimbabwe" OR "northern rhodesia").ti,ab,sh,kf. OR (global south OR africa south of the sahara OR sub-saharan africa OR subsaharan africa OR africa, central OR central africa OR africa, northern OR north africa OR northern africa OR magreb OR maghrib OR sahara OR africa, southern OR southern africa OR africa, eastern OR east africa OR eastern africa OR africa, western OR west africa OR western africa OR west indies OR indian ocean islands OR caribbean OR central america OR latin america OR "south and central america" OR south america OR asia, central OR central asia OR asia, northern OR north asia OR northern asia OR asia, southeastern OR southeastern asia OR south eastern asia OR southeast asia OR south east asia OR asia, western OR western asia OR europe, eastern OR east europe OR eastern europe OR developing country OR developing countries OR developing nation? OR developing population? OR developing world OR less developed countr* OR less developed nation? OR less developed population? OR less developed world OR lesser developed countr* OR lesser developed nation? OR lesser developed population? OR lesser developed world OR under developed countr* OR under developed nation? OR under developed population? OR under developed world OR underdeveloped countr* OR underdeveloped nation? OR underdeveloped population? OR underdeveloped world OR middle income countr* OR middle income nation? OR middle income population? OR low income countr* OR low income nation? OR low income population? OR lower income countr* OR lower income nation? OR lower income population? OR underserved countr* OR underserved nation? OR underserved population? OR underserved world OR under served countr* OR under served nation? OR under served population? OR under served world OR deprived countr* OR deprived nation? OR deprived population? OR deprived world OR poor countr* OR poor nation? OR poor population? OR poor world OR poorer countr* OR poorer nation? OR poorer population? OR poorer world OR developing econom* OR less developed econom* OR lesser developed econom* OR under developed econom* OR underdeveloped econom* OR middle income econom* OR low income econom* OR lower income econom* OR low gdp OR low gnp OR low gross domestic OR low gross national OR lower gdp OR lower gnp OR lower gross domestic OR lower gross national OR lmic OR lmics OR third world OR lami countr* OR transitional countr* OR emerging economies OR emerging nation?).ti,ab,sh,kf. | 2,706,157 |
| **4. Combo** | 1 AND 2 AND 3 | 3,075 |
| **5. Limit** | (exp Animals/ NOT Humans/) |  |
| **6. Limit Combo** | 4 NOT 5 |  |
| **7. Limit** | (Comment OR Clinical Trial Protocol OR Editorial OR Preprint).pt. |  |
| **8. Limit Combo** | 6 NOT 7 |  |
| **9. Limit** | Limit Line 8 to Publication Years: 2000-Current |  |
| **Limits** | AND NOT (exp Animals/ NOT Humans/)  AND 2000-Current  AND NOT (Comment OR Clinical Trial Protocol OR Editorial OR Preprint).pt. |  |
| **Total** |  | 2,549 |

| Ovid Embase Classic+Embase 1947 to 2025 July 01 | | |
| --- | --- | --- |
| **Date Searched** | 7/2/2025 | |
| **Concept** | **Search String** | **Results** |
| **1. ID Interventions** | ((communicable disease*.ti,ab. OR  infectious disease*.ti,ab. OR  neglected disease*.ti,ab. OR  neglected tropical disease*.ti,ab. OR  vaccine preventable disease*.ti,ab. OR  exp Communicable Disease/ OR  Neglected Disease/ OR  Vaccine Preventable Disease/ OR  exp Disease Transmission/ OR  Communicable Disease Control/) AND  (mass screening.ti,ab. OR  screening.ti,ab. OR  face mask*.ti,ab. OR  "Test Treat and Track".ti,ab. OR  "Test Treat & Track".ti,ab. OR  "Test and Treat".ti,ab. OR  "Test & Treat".ti,ab. OR  Mandatory Testing/ OR  Mass Screening/)) OR  (bednet* OR  bed net* OR  condom* OR  contact tracing OR  deworming OR  immuniz* OR  immunis* OR  inoculat* OR  mass drug administration OR  preventive chemotherap* OR  vaccin* OR  insecticide treated net* OR  mosquito net OR  mosquito nets OR  "Surgery, Antibiotics, Facial cleanliness and Environmental improvements" OR  "Surgery, Antibiotics, Facial cleanliness & Environmental improvements" OR  (SAFE ADJ4 (World Health Organization OR WHO))).ti,ab,kf. OR  exp antiinfective agent/ OR  Condom/ OR  Contact Examination/ OR  Disease Eradication/ OR  Insecticide Treated Net/ OR  Bed Net/ OR  Immunization/ OR  preventive health service/ OR  Mass Drug Administration/ OR  Mass Immunization/ OR  Post Exposure Prophylaxis/ OR  Pre-Exposure Prophylaxis/ OR  Vaccination/ OR  exp Vaccine/ | 6,512,208 |
| **2. Low-Income and Urban** | ((cities OR  city OR  municipalit* OR  peri urban* OR  periurban* OR  township* OR  urban*)  AND  ("aashwa'i" OR  arrabal OR  asentamiento* OR  barraca OR  brarek OR  campamento* OR  ciudad perdida OR  comuna OR  comunas OR  habitat precaire OR  habitat spontane OR  homeless camp OR  homeless encampment OR  hovel* OR  human settlement development OR  illegal settlement* OR  illegal shack* OR  imijondolo* OR  immigrant camp OR  immigrant camps OR  immigrant settlement OR  immigrant settlements OR  impoverish* OR  informal encampment* OR  informal housing OR  informal settlement* OR  informal shack* OR  irregular settlement* OR  irregular shack* OR  katra OR  kijiji OR  loteamento OR  low income OR  low socioeconomic OR  migrant camp OR  migrant camps OR  migrant settlement* OR  poblacion callampa OR  poor OR  poverty OR  precario OR  public squalor OR  quartier irregulier OR  refugee camp OR  refugee camps OR  refugee settlement* OR  rundown settlement* OR  shack dweller* OR  shackdweller* OR  shack settlement* OR  shack town* OR  shanties OR  shanty OR  shantyhouse* OR  skid row OR  squalid accommodation OR  squalid housing OR  squatter camp* OR  street dweller* OR  street encampment* OR  tanake OR  tenement district* OR  tenement hous* OR  tent city OR  tent cities OR  tugurio* OR  umjondolo)).ti,ab,kf. OR  (ahata OR  barriada* OR  barrio baja OR  barrio pobre OR  basti* OR  bidon OR  bidons OR  bidonville* OR  bustee* OR  cantegril* OR  cardboard city OR  chacarita OR  chawl OR  chawls OR  cite soleil OR  colonia popular OR  comunidade OR  conventillos OR  deponija OR  dharavi OR  edina achouaia OR  elendsviertel OR  favela* OR  foundouks OR  gecekondu* OR  ghetto OR  ghettos OR  informal city OR  informal cities OR  informal urban settlement* OR  jhopadpatti OR  jihuggi OR  kampung OR  kartonsko naselje OR  katchi abadi* OR  khayelitsha OR  kibera OR  lahbach OR  mabanda OR  masseque* OR  morro OR  mudun safi OR  orangi town OR  pelli gewal OR  pueblos joven* OR  rundown neighborhood* OR  rundown neighbourhood* OR  shantytown* OR  slum OR  slums OR  squatter cit* OR  taudi OR  taudis OR  truschobi OR  urban blight OR  urban encampment* OR  urban poor OR  urban slum* OR  urban squat* OR  villa miseria OR  watta).ti,ab,kf. OR  ((Poverty/ OR  Child Poverty/ OR  exp homeless person/)  AND  (Urban Population/ OR  Urban Health/ OR  Urbanization/ OR  City/)) | 60,578 |
| **3. LIMCs** | ("afghanistan" OR "albania" OR "algeria" OR "american samoa" OR "angola" OR "antigua and barbuda" OR "antigua" OR "barbuda" OR "argentina" OR "armenia" OR "armenian" OR "aruba" OR "azerbaijan" OR "bahrain" OR "bangladesh" OR "barbados" OR "republic of belarus" OR "belarus" OR "byelarus" OR "belorussia" OR "byelorussian" OR "belize" OR "british honduras" OR "benin" OR "dahomey" OR "bhutan" OR "bolivia" OR "bosnia and herzegovina" OR "bosnia" OR "herzegovina" OR "botswana" OR "bechuanaland" OR "brazil" OR "brasil" OR "bulgaria" OR "burkina faso" OR "burkina fasso" OR "upper volta" OR "burundi" OR "urundi" OR "cabo verde" OR "cape verde" OR "cambodia" OR "kampuchea" OR "khmer republic" OR "cameroon" OR "cameron" OR "cameroun" OR "central african republic" OR "ubangi shari" OR "chad" OR "chile" OR "china" OR "colombia" OR "comoros" OR "comoro islands" OR "iles comores" OR "mayotte" OR "democratic republic of the congo" OR "democratic republic congo" OR "congo" OR "zaire" OR "costa rica" OR "cote d’ivoire" OR "cote d’ ivoire" OR "cote divoire" OR "cote d ivoire" OR "ivory coast" OR "croatia" OR "cuba" OR "cyprus" OR "czech republic" OR "czechoslovakia" OR "djibouti" OR "french somaliland" OR "dominica" OR "dominican republic" OR "ecuador" OR "egypt" OR "united arab republic" OR "el salvador" OR "equatorial guinea" OR "spanish guinea" OR "eritrea" OR "estonia" OR "eswatini" OR "swaziland" OR "ethiopia" OR "fiji" OR "gabon" OR "gabonese republic" OR "gambia" OR "georgia (republic)" OR "georgian" OR "ghana" OR "gold coast" OR "gibraltar" OR "greece" OR "grenada" OR "guam" OR "guatemala" OR "guinea" OR "guinea bissau" OR "guyana" OR "british guiana" OR "haiti" OR "hispaniola" OR "honduras" OR "hungary" OR "india" OR "indonesia" OR "timor" OR "iran" OR "iraq" OR "isle of man" OR "jamaica" OR "jordan" OR "kazakhstan" OR "kazakh" OR "kenya" OR "democratic people’s republic of korea" OR "republic of korea" OR "north korea" OR "south korea" OR "korea" OR "kosovo" OR "kyrgyzstan" OR "kirghizia" OR "kirgizstan" OR "kyrgyz republic" OR "kirghiz" OR "laos" OR "lao pdr" OR "lao people's democratic republic" OR "latvia" OR "lebanon" OR "lebanese republic" OR "lesotho" OR "basutoland" OR "liberia" OR "libya" OR "libyan arab jamahiriya" OR "lithuania" OR "macau" OR "macao" OR "republic of north macedonia" OR "macedonia" OR "madagascar" OR "malagasy republic" OR "malawi" OR "nyasaland" OR "malaysia" OR "malay federation" OR "malaya federation" OR "maldives" OR "indian ocean islands" OR "mali" OR "malta" OR "micronesia" OR "federated states of micronesia" OR "kiribati" OR "marshall islands" OR "nauru" OR "northern mariana islands" OR "palau" OR "tuvalu" OR "mauritania" OR "mauritius" OR "mexico" OR "moldova" OR "moldovian" OR "mongolia" OR "montenegro" OR "morocco" OR "ifni" OR "mozambique" OR "portuguese east africa" OR "myanmar" OR "burma" OR "namibia" OR "nepal" OR "netherlands antilles" OR "nicaragua" OR "niger" OR "nigeria" OR "oman" OR "muscat" OR "pakistan" OR "panama" OR "papua new guinea" OR "new guinea" OR "paraguay" OR "peru" OR "philippines" OR "philipines" OR "phillipines" OR "phillippines" OR "poland" OR "polish people's republic" OR "portugal" OR "portuguese republic" OR "puerto rico" OR "romania" OR "russia" OR "russian federation" OR "ussr" OR "soviet union" OR "union of soviet socialist republics" OR "rwanda" OR "ruanda" OR "samoa" OR "pacific islands" OR "polynesia" OR "samoan islands" OR "navigator island" OR "navigator islands" OR "sao tome and principe" OR "saudi arabia" OR "senegal" OR "serbia" OR "seychelles" OR "sierra leone" OR "slovakia" OR "slovak republic" OR "slovenia" OR "melanesia" OR "solomon island" OR "solomon islands" OR "norfolk island" OR "norfolk islands" OR "somalia" OR "south africa" OR "south sudan" OR "sri lanka" OR "ceylon" OR "saint kitts and nevis" OR "st. kitts and nevis" OR "saint lucia" OR "st. lucia" OR "saint vincent and the grenadines" OR "saint vincent" OR "st. vincent" OR "grenadines" OR "sudan" OR "suriname" OR "surinam" OR "dutch guiana" OR "netherlands guiana" OR "syria" OR "syrian arab republic" OR "tajikistan" OR "tadjikistan" OR "tadzhikistan" OR "tadzhik" OR "tanzania" OR "tanganyika" OR "thailand" OR "siam" OR "timor leste" OR "east timor" OR "togo" OR "togolese republic" OR "tonga" OR "trinidad and tobago" OR "trinidad" OR "tobago" OR "tunisia" OR "turkey" OR "turkiye" OR "turkmenistan" OR "turkmen" OR "uganda" OR "ukraine" OR "uruguay" OR "uzbekistan" OR "uzbek" OR "vanuatu" OR "new hebrides" OR "venezuela" OR "vietnam" OR "viet nam" OR "middle east" OR "west bank" OR "gaza" OR "palestine" OR "yemen" OR "yugoslavia" OR "zambia" OR "zimbabwe" OR "northern rhodesia").ti,ab,sh,kf. OR (global south OR africa south of the sahara OR sub-saharan africa OR subsaharan africa OR africa, central OR central africa OR africa, northern OR north africa OR northern africa OR magreb OR maghrib OR sahara OR africa, southern OR southern africa OR africa, eastern OR east africa OR eastern africa OR africa, western OR west africa OR western africa OR west indies OR indian ocean islands OR caribbean OR central america OR latin america OR "south and central america" OR south america OR asia, central OR central asia OR asia, northern OR north asia OR northern asia OR asia, southeastern OR southeastern asia OR south eastern asia OR southeast asia OR south east asia OR asia, western OR western asia OR europe, eastern OR east europe OR eastern europe OR developing country OR developing countries OR developing nation? OR developing population? OR developing world OR less developed countr* OR less developed nation? OR less developed population? OR less developed world OR lesser developed countr* OR lesser developed nation? OR lesser developed population? OR lesser developed world OR under developed countr* OR under developed nation? OR under developed population? OR under developed world OR underdeveloped countr* OR underdeveloped nation? OR underdeveloped population? OR underdeveloped world OR middle income countr* OR middle income nation? OR middle income population? OR low income countr* OR low income nation? OR low income population? OR lower income countr* OR lower income nation? OR lower income population? OR underserved countr* OR underserved nation? OR underserved population? OR underserved world OR under served countr* OR under served nation? OR under served population? OR under served world OR deprived countr* OR deprived nation? OR deprived population? OR deprived world OR poor countr* OR poor nation? OR poor population? OR poor world OR poorer countr* OR poorer nation? OR poorer population? OR poorer world OR developing econom* OR less developed econom* OR lesser developed econom* OR under developed econom* OR underdeveloped econom* OR middle income econom* OR low income econom* OR lower income econom* OR low gdp OR low gnp OR low gross domestic OR low gross national OR lower gdp OR lower gnp OR lower gross domestic OR lower gross national OR lmic OR lmics OR third world OR lami countr* OR transitional countr* OR emerging economies OR emerging nation?).ti,ab,sh,kf. | 3,329,692 |
| **4. Combo** | 1 AND 2 AND 3 | 4,854 |
| **5. Limit** | (exp Animal/ NOT Human/) |  |
| **6. Limit Combo** | 4 NOT 5 |  |
| **7. Limit** | (Case Report/ OR Clinical Trial.pt. OR Editorial.pt. OR Preprint.pt. OR Abstract.pt. OR Conference.pt. OR Conference Abstract.pt.) |  |
| **8. Limit Combo** | 6 NOT 7 |  |
| **9. Limit** | Limit Line 8 to dc=20000101-20251231 |  |
| **10. Limit** | Remove MEDLINE Records |  |
| **Total** |  | 812 |

| Ovid Global Health 1973 to 2025 Week 26 | | |
| --- | --- | --- |
| **Date Searched** | 7/2/2025 | |
| **Concept** | **Search String** | **Results** |
| **1. ID Interventions** | ((communicable disease*.ti,ab. OR  infectious disease*.ti,ab. OR  neglected disease*.ti,ab. OR  neglected tropical disease*.ti,ab. OR  vaccine preventable disease*.ti,ab. OR  exp infectious diseases/ OR  neglected tropical diseases/) AND  (mass screening.ti,ab. OR  screening.ti,ab. OR  face mask*.ti,ab. OR  "Test Treat and Track".ti,ab. OR  "Test Treat & Track".ti,ab. OR  "Test and Treat".ti,ab. OR  "Test & Treat".ti,ab. OR  exp Screening/)) OR  (bednet* OR  bed net* OR  condom* OR  contact tracing OR  deworming OR  immuniz* OR  immunis* OR  inoculat* OR  mass drug administration OR  preventive chemotherap* OR  vaccin* OR  insecticide treated net* OR  mosquito net OR  mosquito nets OR  "Surgery, Antibiotics, Facial cleanliness and Environmental improvements" OR  "Surgery, Antibiotics, Facial cleanliness & Environmental improvements" OR  (SAFE ADJ4 (World Health Organization OR WHO))).ti,ab. OR  antiinfective agents/ OR  Condoms/ OR  Contact Tracing/ OR  exp Bed Nets/ OR  Mosquito Nets/ OR  Immunization/ OR  immunization programmes/ OR  Mass Drug Administration/ OR  Mass Vaccination/ OR  Pre-Exposure Prophylaxis/ OR  Vaccination/ OR  exp Vaccines/ | 415,535 |
| **2. Low-Income and Urban** | ((cities OR  city OR  municipalit* OR  peri urban* OR  periurban* OR  township* OR  urban*)  AND  ("aashwa'i" OR  arrabal OR  asentamiento* OR  barraca OR  brarek OR  campamento* OR  ciudad perdida OR  comuna OR  comunas OR  habitat precaire OR  habitat spontane OR  homeless camp OR  homeless encampment OR  hovel* OR  human settlement development OR  illegal settlement* OR  illegal shack* OR  imijondolo* OR  immigrant camp OR  immigrant camps OR  immigrant settlement OR  immigrant settlements OR  impoverish* OR  informal encampment* OR  informal housing OR  informal settlement* OR  informal shack* OR  irregular settlement* OR  irregular shack* OR  katra OR  kijiji OR  loteamento OR  low income OR  low socioeconomic OR  migrant camp OR  migrant camps OR  migrant settlement* OR  poblacion callampa OR  poor OR  poverty OR  precario OR  public squalor OR  quartier irregulier OR  refugee camp OR  refugee camps OR  refugee settlement* OR  rundown settlement* OR  shack dweller* OR  shackdweller* OR  shack settlement* OR  shack town* OR  shanties OR  shanty OR  shantyhouse* OR  skid row OR  squalid accommodation OR  squalid housing OR  squatter camp* OR  street dweller* OR  street encampment* OR  tanake OR  tenement district* OR  tenement hous* OR  tent city OR  tent cities OR  tugurio* OR  umjondolo)).ti,ab. OR  (ahata OR  barriada* OR  barrio baja OR  barrio pobre OR  basti* OR  bidon OR  bidons OR  bidonville* OR  bustee* OR  cantegril* OR  cardboard city OR  chacarita OR  chawl OR  chawls OR  cite soleil OR  colonia popular OR  comunidade OR  conventillos OR  deponija OR  dharavi OR  edina achouaia OR  elendsviertel OR  favela* OR  foundouks OR  gecekondu* OR  ghetto OR  ghettos OR  informal city OR  informal cities OR  informal urban settlement* OR  jhopadpatti OR  jihuggi OR  kampung OR  kartonsko naselje OR  katchi abadi* OR  khayelitsha OR  kibera OR  lahbach OR  mabanda OR  masseque* OR  morro OR  mudun safi OR  orangi town OR  pelli gewal OR  pueblos joven* OR  rundown neighborhood* OR  rundown neighbourhood* OR  shantytown* OR  slum OR  slums OR  squatter cit* OR  taudi OR  taudis OR  truschobi OR  urban blight OR  urban encampment* OR  urban poor OR  urban slum* OR  urban squat* OR  villa miseria OR  watta).ti,ab. OR  ((Economically Disadvantaged/ OR  Landlessness/ OR  Poverty/) AND  (Urban Population/ OR  Urban Areas/ OR  Urbanization/ OR  Cities/)) | 36,580 |
| **3. LIMCs** | ("afghanistan" OR "albania" OR "algeria" OR "american samoa" OR "angola" OR "antigua and barbuda" OR "antigua" OR "barbuda" OR "argentina" OR "armenia" OR "armenian" OR "aruba" OR "azerbaijan" OR "bahrain" OR "bangladesh" OR "barbados" OR "republic of belarus" OR "belarus" OR "byelarus" OR "belorussia" OR "byelorussian" OR "belize" OR "british honduras" OR "benin" OR "dahomey" OR "bhutan" OR "bolivia" OR "bosnia and herzegovina" OR "bosnia" OR "herzegovina" OR "botswana" OR "bechuanaland" OR "brazil" OR "brasil" OR "bulgaria" OR "burkina faso" OR "burkina fasso" OR "upper volta" OR "burundi" OR "urundi" OR "cabo verde" OR "cape verde" OR "cambodia" OR "kampuchea" OR "khmer republic" OR "cameroon" OR "cameron" OR "cameroun" OR "central african republic" OR "ubangi shari" OR "chad" OR "chile" OR "china" OR "colombia" OR "comoros" OR "comoro islands" OR "iles comores" OR "mayotte" OR "democratic republic of the congo" OR "democratic republic congo" OR "congo" OR "zaire" OR "costa rica" OR "cote d’ivoire" OR "cote d’ ivoire" OR "cote divoire" OR "cote d ivoire" OR "ivory coast" OR "croatia" OR "cuba" OR "cyprus" OR "czech republic" OR "czechoslovakia" OR "djibouti" OR "french somaliland" OR "dominica" OR "dominican republic" OR "ecuador" OR "egypt" OR "united arab republic" OR "el salvador" OR "equatorial guinea" OR "spanish guinea" OR "eritrea" OR "estonia" OR "eswatini" OR "swaziland" OR "ethiopia" OR "fiji" OR "gabon" OR "gabonese republic" OR "gambia" OR "georgia (republic)" OR "georgian" OR "ghana" OR "gold coast" OR "gibraltar" OR "greece" OR "grenada" OR "guam" OR "guatemala" OR "guinea" OR "guinea bissau" OR "guyana" OR "british guiana" OR "haiti" OR "hispaniola" OR "honduras" OR "hungary" OR "india" OR "indonesia" OR "timor" OR "iran" OR "iraq" OR "isle of man" OR "jamaica" OR "jordan" OR "kazakhstan" OR "kazakh" OR "kenya" OR "democratic people’s republic of korea" OR "republic of korea" OR "north korea" OR "south korea" OR "korea" OR "kosovo" OR "kyrgyzstan" OR "kirghizia" OR "kirgizstan" OR "kyrgyz republic" OR "kirghiz" OR "laos" OR "lao pdr" OR "lao people's democratic republic" OR "latvia" OR "lebanon" OR "lebanese republic" OR "lesotho" OR "basutoland" OR "liberia" OR "libya" OR "libyan arab jamahiriya" OR "lithuania" OR "macau" OR "macao" OR "republic of north macedonia" OR "macedonia" OR "madagascar" OR "malagasy republic" OR "malawi" OR "nyasaland" OR "malaysia" OR "malay federation" OR "malaya federation" OR "maldives" OR "indian ocean islands" OR "mali" OR "malta" OR "micronesia" OR "federated states of micronesia" OR "kiribati" OR "marshall islands" OR "nauru" OR "northern mariana islands" OR "palau" OR "tuvalu" OR "mauritania" OR "mauritius" OR "mexico" OR "moldova" OR "moldovian" OR "mongolia" OR "montenegro" OR "morocco" OR "ifni" OR "mozambique" OR "portuguese east africa" OR "myanmar" OR "burma" OR "namibia" OR "nepal" OR "netherlands antilles" OR "nicaragua" OR "niger" OR "nigeria" OR "oman" OR "muscat" OR "pakistan" OR "panama" OR "papua new guinea" OR "new guinea" OR "paraguay" OR "peru" OR "philippines" OR "philipines" OR "phillipines" OR "phillippines" OR "poland" OR "polish people's republic" OR "portugal" OR "portuguese republic" OR "puerto rico" OR "romania" OR "russia" OR "russian federation" OR "ussr" OR "soviet union" OR "union of soviet socialist republics" OR "rwanda" OR "ruanda" OR "samoa" OR "pacific islands" OR "polynesia" OR "samoan islands" OR "navigator island" OR "navigator islands" OR "sao tome and principe" OR "saudi arabia" OR "senegal" OR "serbia" OR "seychelles" OR "sierra leone" OR "slovakia" OR "slovak republic" OR "slovenia" OR "melanesia" OR "solomon island" OR "solomon islands" OR "norfolk island" OR "norfolk islands" OR "somalia" OR "south africa" OR "south sudan" OR "sri lanka" OR "ceylon" OR "saint kitts and nevis" OR "st. kitts and nevis" OR "saint lucia" OR "st. lucia" OR "saint vincent and the grenadines" OR "saint vincent" OR "st. vincent" OR "grenadines" OR "sudan" OR "suriname" OR "surinam" OR "dutch guiana" OR "netherlands guiana" OR "syria" OR "syrian arab republic" OR "tajikistan" OR "tadjikistan" OR "tadzhikistan" OR "tadzhik" OR "tanzania" OR "tanganyika" OR "thailand" OR "siam" OR "timor leste" OR "east timor" OR "togo" OR "togolese republic" OR "tonga" OR "trinidad and tobago" OR "trinidad" OR "tobago" OR "tunisia" OR "turkey" OR "turkiye" OR "turkmenistan" OR "turkmen" OR "uganda" OR "ukraine" OR "uruguay" OR "uzbekistan" OR "uzbek" OR "vanuatu" OR "new hebrides" OR "venezuela" OR "vietnam" OR "viet nam" OR "middle east" OR "west bank" OR "gaza" OR "palestine" OR "yemen" OR "yugoslavia" OR "zambia" OR "zimbabwe" OR "northern rhodesia").ti,ab,sh. OR (global south OR africa south of the sahara OR sub-saharan africa OR subsaharan africa OR africa, central OR central africa OR africa, northern OR north africa OR northern africa OR magreb OR maghrib OR sahara OR africa, southern OR southern africa OR africa, eastern OR east africa OR eastern africa OR africa, western OR west africa OR western africa OR west indies OR indian ocean islands OR caribbean OR central america OR latin america OR "south and central america" OR south america OR asia, central OR central asia OR asia, northern OR north asia OR northern asia OR asia, southeastern OR southeastern asia OR south eastern asia OR southeast asia OR south east asia OR asia, western OR western asia OR europe, eastern OR east europe OR eastern europe OR developing country OR developing countries OR developing nation? OR developing population? OR developing world OR less developed countr* OR less developed nation? OR less developed population? OR less developed world OR lesser developed countr* OR lesser developed nation? OR lesser developed population? OR lesser developed world OR under developed countr* OR under developed nation? OR under developed population? OR under developed world OR underdeveloped countr* OR underdeveloped nation? OR underdeveloped population? OR underdeveloped world OR middle income countr* OR middle income nation? OR middle income population? OR low income countr* OR low income nation? OR low income population? OR lower income countr* OR lower income nation? OR lower income population? OR underserved countr* OR underserved nation? OR underserved population? OR underserved world OR under served countr* OR under served nation? OR under served population? OR under served world OR deprived countr* OR deprived nation? OR deprived population? OR deprived world OR poor countr* OR poor nation? OR poor population? OR poor world OR poorer countr* OR poorer nation? OR poorer population? OR poorer world OR developing econom* OR less developed econom* OR lesser developed econom* OR under developed econom* OR underdeveloped econom* OR middle income econom* OR low income econom* OR lower income econom* OR low gdp OR low gnp OR low gross domestic OR low gross national OR lower gdp OR lower gnp OR lower gross domestic OR lower gross national OR lmic OR lmics OR third world OR lami countr* OR transitional countr* OR emerging economies OR emerging nation?).ti,ab,sh. | 2,045,883 |
| **4. Combo** | 1 AND 2 AND 3 | 2,453 |
| **5.** | (exp Vertebrates/ NOT Man/) |  |
| **6.** | 4 NOT 5 |  |
| **7.** | (Abstract Only OR Conference Proceeding OR Editorial OR Preprint).pt. |  |
| **8.** | 6 NOT 7 |  |
| **9.** | Limit Line 8 to Publication Years: 2000-Current |  |
| **Total** |  | 2,218 |

| Web of Science Core Collection | | |
| --- | --- | --- |
| **Date Searched** | 7/2/2025 | |
| **Concept** | **Search String** | **Results** |
| **1. ID Interventions** | TS=(("communicable disease*" OR  "infectious disease*" OR  "neglected disease*" OR  "neglected tropical disease*" OR  "vaccine preventable disease*") AND  ("mass screening" OR  "screening" OR  "face mask*" OR  "Test Treat and Track" OR  "Test Treat & Track" OR  "Test and Treat" OR  "Test & Treat")) OR  TS=("bednet*"OR  "bed net*" OR  "condom*" OR  "contact tracing" OR  "deworming" OR  "immuniz*" OR  "immunis*" OR  "inoculat*" OR  "mass drug administration" OR  "preventive chemotherap*" OR  "vaccin*" OR  "insecticide treated net*" OR  "mosquito net" OR  "mosquito nets" OR  "Surgery, Antibiotics, Facial cleanliness and Environmental improvements" OR  "Surgery, Antibiotics, Facial cleanliness & Environmental improvements" OR  ("SAFE" NEAR/4 ("World Health Organization" OR "WHO"))) | 856,205 |
| **2. Low-Income and Urban** | TS=(("cities" OR  "city" OR  "municipalit*" OR  "peri urban*" OR  "periurban*" OR  "township*" OR  "urban*")  AND  ("aashwa'i" OR  "arrabal" OR  "asentamiento*" OR  "barraca" OR  "brarek" OR  "campamento*" OR  "ciudad perdida" OR  "comuna" OR  "comunas" OR  "habitat precaire" OR  "habitat spontane" OR  "homeless camp" OR  "homeless encampment" OR  "hovel*" OR  "human settlement development" OR  "illegal settlement*" OR  "illegal shack*" OR  "imijondolo*" OR  "immigrant camp" OR  "immigrant camps" OR  "immigrant settlement" OR  "immigrant settlements" OR  "impoverish*" OR  "informal encampment*" OR  "informal housing" OR  "informal settlement*" OR  "informal shack*" OR  "irregular settlement*" OR  "irregular shack*" OR  "katra" OR  "kijiji" OR  "loteamento" OR  "low income" OR  "low socioeconomic" OR  "migrant camp" OR  "migrant camps" OR  "migrant settlement*" OR  "poblacion callampa" OR  "poor" OR  "poverty" OR  "precario" OR  "public squalor" OR  "quartier irregulier" OR  "refugee camp" OR  "refugee camps" OR  "refugee settlement*" OR  "rundown settlement*" OR  "shack dweller*" OR  "shackdweller*" OR  "shack settlement*" OR  "shack town*" OR  "shanties" OR  "shanty" OR  "shantyhouse*" OR  "skid row" OR  "squalid accommodation" OR  "squalid housing" OR  "squatter camp*" OR  "street dweller*" OR  "street encampment*" OR  "tanake" OR  "tenement district*" OR  "tenement hous*" OR  "tent city" OR  "tent cities" OR  "tugurio*" OR  "umjondolo")) OR  TS=("ahata" OR  "barriada*" OR  "barrio baja" OR  "barrio pobre" OR  "basti*" OR  "bidon" OR  "bidons" OR  "bidonville*" OR  "bustee*" OR  "cantegril*" OR  "cardboard city" OR  "chacarita" OR  "chawl" OR  "chawls" OR  "cite soleil" OR  "colonia popular" OR  "comunidade" OR  "conventillos" OR  "deponija" OR  "dharavi" OR  "edina achouaia" OR  "elendsviertel" OR  "favela*" OR  "foundouks" OR  "gecekondu*" OR  "ghetto" OR  "ghettos" OR  "informal city" OR  "informal cities" OR  "informal urban settlement*" OR  "jhopadpatti" OR  "jihuggi" OR  "kampung" OR  "kartonsko naselje" OR  "katchi abadi*" OR  "khayelitsha" OR  "kibera" OR  "lahbach" OR  "mabanda" OR  "masseque*" OR  "morro" OR  "mudun safi" OR  "orangi town" OR  "pelli gewal" OR  "pueblos joven*" OR  "rundown neighborhood*" OR  "rundown neighbourhood*" OR  "shantytown*" OR  "slum" OR  "slums" OR  "squatter cit*" OR  "taudi" OR  "taudis" OR  "truschobi" OR  "urban blight" OR  "urban encampment*" OR  "urban poor" OR  "urban slum*" OR  "urban squat*" OR  "villa miseria" OR  "watta") | 95,944 |
| **3. LIMCs** | TS=("afghanistan" OR "albania" OR "algeria" OR "american samoa" OR "angola" OR "antigua and barbuda" OR "antigua" OR "barbuda" OR "argentina" OR "armenia" OR "armenian" OR "aruba" OR "azerbaijan" OR "bahrain" OR "bangladesh" OR "barbados" OR "republic of belarus" OR "belarus" OR "byelarus" OR "belorussia" OR "byelorussian" OR "belize" OR "british honduras" OR "benin" OR "dahomey" OR "bhutan" OR "bolivia" OR "bosnia and herzegovina" OR "bosnia" OR "herzegovina" OR "botswana" OR "bechuanaland" OR "brazil" OR "brasil" OR "bulgaria" OR "burkina faso" OR "burkina fasso" OR "upper volta" OR "burundi" OR "urundi" OR "cabo verde" OR "cape verde" OR "cambodia" OR "kampuchea" OR "khmer republic" OR "cameroon" OR "cameron" OR "cameroun" OR "central african republic" OR "ubangi shari" OR "chad" OR "chile" OR "china" OR "colombia" OR "comoros" OR "comoro islands" OR "iles comores" OR "mayotte" OR "democratic republic of the congo" OR "democratic republic congo" OR "congo" OR "zaire" OR "costa rica" OR "cote d’ivoire" OR "cote d’ ivoire" OR "cote divoire" OR "cote d ivoire" OR "ivory coast" OR "croatia" OR "cuba" OR "cyprus" OR "czech republic" OR "czechoslovakia" OR "djibouti" OR "french somaliland" OR "dominica" OR "dominican republic" OR "ecuador" OR "egypt" OR "united arab republic" OR "el salvador" OR "equatorial guinea" OR "spanish guinea" OR "eritrea" OR "estonia" OR "eswatini" OR "swaziland" OR "ethiopia" OR "fiji" OR "gabon" OR "gabonese republic" OR "gambia" OR "georgia (republic)" OR "georgian" OR "ghana" OR "gold coast" OR "gibraltar" OR "greece" OR "grenada" OR "guam" OR "guatemala" OR "guinea" OR "guinea bissau" OR "guyana" OR "british guiana" OR "haiti" OR "hispaniola" OR "honduras" OR "hungary" OR "india" OR "indonesia" OR "timor" OR "iran" OR "iraq" OR "isle of man" OR "jamaica" OR "jordan" OR "kazakhstan" OR "kazakh" OR "kenya" OR "democratic people’s republic of korea" OR "republic of korea" OR "north korea" OR "south korea" OR "korea" OR "kosovo" OR "kyrgyzstan" OR "kirghizia" OR "kirgizstan" OR "kyrgyz republic" OR "kirghiz" OR "laos" OR "lao pdr" OR "lao people's democratic republic" OR "latvia" OR "lebanon" OR "lebanese republic" OR "lesotho" OR "basutoland" OR "liberia" OR "libya" OR "libyan arab jamahiriya" OR "lithuania" OR "macau" OR "macao" OR "republic of north macedonia" OR "macedonia" OR "madagascar" OR "malagasy republic" OR "malawi" OR "nyasaland" OR "malaysia" OR "malay federation" OR "malaya federation" OR "maldives" OR "indian ocean islands" OR "mali" OR "malta" OR "micronesia" OR "federated states of micronesia" OR "kiribati" OR "marshall islands" OR "nauru" OR "northern mariana islands" OR "palau" OR "tuvalu" OR "mauritania" OR "mauritius" OR "mexico" OR "moldova" OR "moldovian" OR "mongolia" OR "montenegro" OR "morocco" OR "ifni" OR "mozambique" OR "portuguese east africa" OR "myanmar" OR "burma" OR "namibia" OR "nepal" OR "netherlands antilles" OR "nicaragua" OR "niger" OR "nigeria" OR "oman" OR "muscat" OR "pakistan" OR "panama" OR "papua new guinea" OR "new guinea" OR "paraguay" OR "peru" OR "philippines" OR "philipines" OR "phillipines" OR "phillippines" OR "poland" OR "polish people's republic" OR "portugal" OR "portuguese republic" OR "puerto rico" OR "romania" OR "russia" OR "russian federation" OR "ussr" OR "soviet union" OR "union of soviet socialist republics" OR "rwanda" OR "ruanda" OR "samoa" OR "pacific islands" OR "polynesia" OR "samoan islands" OR "navigator island" OR "navigator islands" OR "sao tome and principe" OR "saudi arabia" OR "senegal" OR "serbia" OR "seychelles" OR "sierra leone" OR "slovakia" OR "slovak republic" OR "slovenia" OR "melanesia" OR "solomon island" OR "solomon islands" OR "norfolk island" OR "norfolk islands" OR "somalia" OR "south africa" OR "south sudan" OR "sri lanka" OR "ceylon" OR "saint kitts and nevis" OR "st. kitts and nevis" OR "saint lucia" OR "st. lucia" OR "saint vincent and the grenadines" OR "saint vincent" OR "st. vincent" OR "grenadines" OR "sudan" OR "suriname" OR "surinam" OR "dutch guiana" OR "netherlands guiana" OR "syria" OR "syrian arab republic" OR "tajikistan" OR "tadjikistan" OR "tadzhikistan" OR "tadzhik" OR "tanzania" OR "tanganyika" OR "thailand" OR "siam" OR "timor leste" OR "east timor" OR "togo" OR "togolese republic" OR "tonga" OR "trinidad and tobago" OR "trinidad" OR "tobago" OR "tunisia" OR "turkey" OR "turkiye" OR "turkmenistan" OR "turkmen" OR "uganda" OR "ukraine" OR "uruguay" OR "uzbekistan" OR "uzbek" OR "vanuatu" OR "new hebrides" OR "venezuela" OR "vietnam" OR "viet nam" OR "middle east" OR "west bank" OR "gaza" OR "palestine" OR "yemen" OR "yugoslavia" OR "zambia" OR "zimbabwe" OR "northern rhodesia") OR TS=("global south" OR "africa south of the sahara" OR "sub-saharan africa" OR "subsaharan africa" OR "africa, central" OR "central africa" OR "africa, northern" OR "north africa" OR "northern africa" OR "magreb" OR "maghrib" OR "sahara" OR "africa, southern" OR "southern africa" OR "africa, eastern" OR "east africa" OR "eastern africa" OR "africa, western" OR "west africa" OR "western africa" OR "west indies" OR "indian ocean islands" OR "caribbean" OR "central america" OR "latin america" OR "south and central america" OR "south america" OR "asia, central" OR "central asia" OR "asia, northern" OR "north asia" OR "northern asia" OR "asia, southeastern" OR "southeastern asia" OR "south eastern asia" OR "southeast asia" OR "south east asia" OR "asia, western" OR "western asia" OR "europe, eastern" OR "east europe" OR "eastern europe" OR "developing country" OR "developing countries" OR "developing nation?" OR "developing population?" OR "developing world" OR "less developed countr*" OR "less developed nation?" OR "less developed population?" OR "less developed world" OR "lesser developed countr*" OR "lesser developed nation?" OR "lesser developed population?" OR "lesser developed world" OR "under developed countr*" OR "under developed nation?" OR "under developed population?" OR "under developed world" OR "underdeveloped countr*" OR "underdeveloped nation?" OR "underdeveloped population?" OR "underdeveloped world" OR "middle income countr*" OR "middle income nation?" OR "middle income population?" OR "low income countr*" OR "low income nation?" OR "low income population?" OR "lower income countr*" OR "lower income nation?" OR "lower income population?" OR "underserved countr*" OR "underserved nation?" OR "underserved population?" OR "underserved world" OR "under served countr*" OR "under served nation?" OR "under served population?" OR "under served world" OR "deprived countr*" OR "deprived nation?" OR "deprived population?" OR "deprived world" OR "poor countr*" OR "poor nation?" OR "poor population?" OR "poor world" OR "poorer countr*" OR "poorer nation?" OR "poorer population?" OR "poorer world" OR "developing econom*" OR "less developed econom*" OR "lesser developed econom*" OR "under developed econom*" OR "underdeveloped econom*" OR "middle income econom*" OR "low income econom*" OR "lower income econom*" OR "low gdp" OR "low gnp" OR "low gross domestic" OR "low gross national" OR "lower gdp" OR "lower gnp" OR "lower gross domestic" OR "lower gross national" OR "lmic" OR "lmics" OR "third world" OR "lami countr*" OR "transitional countr*" OR "emerging economies" OR "emerging nation?") | 5,450,162 |
| **4. Combo** | 1 AND 2 AND 3 | 1,866 |
| **5. Limit** | TS=((animal* OR rat OR rats OR mouse OR mice OR murine OR dog OR dogs OR canine OR cat OR cats OR feline OR rabbit OR cow OR cows OR bovine OR rodent* OR sheep OR ovine OR pig OR swine OR porcine OR veterinar* OR chick* OR zebrafish* OR baboon* OR nonhuman* OR primate* OR cattle* OR goose OR geese OR duck OR macaque* OR avian* OR bird* OR fish*) NOT (human* OR patient* OR women OR woman OR men OR man)) |  |
| **6. Limit Combo** | #4 NOT #5 |  |
| **7. Limit** | TI=("Trial Protocol") |  |
| **8. Limit Combo** | #6 NOT #7 |  |
| **9. Limit** | DOP=2000-01-01/2025-12-31 |  |
| **10. Limit Combo** | #8 NOT #9 |  |
| **11. Limit** | Exclude (Editorial Material OR Meeting Abstract) |  |
| **Total** |  | 1,695 |
