# Supplementary material for: Exploring how infectious diseases control interventions are delivered in low-income urban communities in low- and middle-income countries: A scoping review protocol
Source: PLoS One. 2025 Dec 10;20(12):e0337949. doi: 10.1371/journal.pone.0337949 (PMC12694824; doi:10.1371/journal.pone.0337949)
Supplement: Attachment — Submitted filename: PONE-D-25-40772_R1_reviewed by Mickael Essouma.pdf [file pone.0337949.s007.pdf]

PLOS ONE

Exploring how infectious diseases control interventions are delivered in low-income urban communities in in low- and middle-income countries: a scoping review protocol

--Manuscript Draft--

**Manuscript Number:**	PONE-D-25-40772R1
**Article Type:**	Study Protocol
**Full Title:**	Exploring how infectious diseases control interventions are delivered in low-income urban communities in in low- and middle-income countries: a scoping review protocol
**Short Title:**	Exploring how infectious diseases control interventions are delivered in urban poor community settings
**Corresponding Author:**	Hammed Oladeji Mogaji, Ph.D<br>Marian University<br>UNITED STATES OF AMERICA
**Keywords:**	Infectious Diseases; Urban poor; Low Middle Income Countries; Interpersonal Interventions
**Abstract:**	Infectious diseases thrive in densely populated low-income urban communities where living conditions, hygiene, and access to health services are sub-optimal. Information on how to adapt infectious diseases control interventions to be effective in these settings is limited. The proposed scoping review will follow Joanna Briggs Institute (JBI) guidelines and the PRISMA methodology adapted for scoping reviews (PRISMA-ScR). Research questions were created using the Person, Concept, Context (PCC) framework. In this study person refers to low-income urban communities, concept refers to community-based infectious disease interventions adapted to their setting, and the context is low- and middle-income countries. Relevant papers published after 2000 will be retrieved using a search strategy that incorporates all the PCC topics. Searches will be made in four databases: MEDLINE, Embase, Global Health (all via Ovid), and Web of Science. Duplicates will be identified and removed with EndNote 20, and all articles will be uploaded into Covidence for screening in two stages using pre-defined inclusion and exclusion criteria. Quantitative and qualitative data will be extracted into Excel spreadsheets and analyzed. Our review will identify different approaches that have been taken to adapt infectious disease interventions in low-income urban communities of LMICs that can be further tested in future programs and studies. We will also highlight methods that have been used to study such interventions in their context.
**Order of Authors:**	H O Mogaji, Ph.D
	I Fatah
	C S Dorris
	M C Baker
**Opposed Reviewers:**	
**Response to Reviewers:**	Additional Editor Comments :<br><br>C1: In addition to the reviewer's comments, I advise the authors to thoroughly revise their manuscript showing creativity so that the aim of their upcoming scoping review and the methodology used to debelop it differ substantially from two recent major reviews in the same research domain:<br><br>1. Epling et al. Lancet Infect Dis. 2025 May;25(5):e269-e279. doi: 10.1016/S1473-3099(24)00744-8.<br>2. Shafique et l. Syst Rev. 2024 Oct 4;13(1):253. doi: 10.1186/s13643-024-02651-9.<br><br>More details are available in the word document PONE-D-25-40772_reviewed by Mickael Essouma attached to this decision letter.<br><br>Response to Reviewer

Powered by Editorial Manager® and ProduXion Manager® from Aries Systems Corporation

	We appreciate the reviewer's suggestion to clarify how our scoping review differs from recent reviews in this area, particularly Epling et al. (Lancet Infect Dis, 2025) – authored by M Baker, also an author of this review - and Shafique et al. (Syst Rev, 2024).<br>This study focuses on populations living in low income urban settings in LMICs while the Epling et al. study focused on populations marginalized by ethnicity, race, or religion - globally. These are intended to be part of a series of papers exploring adaptations of interventions for marginalized populations – with each paper focusing on a different marginalized population, while looking across diseases.<br>Shafique et al. synthesized community-based interventions for infectious disease control in urban informal settlements in LMICs, identifying a range of interventions. However, their review did not examine how these interventions were adapted to fit urban poor settings. Our study addresses this gap by focusing specifically on adaptation processes and mechanisms of delivery in urban low income settings in LMICs.<br><br>This paper is therefore distinct in two ways:<br>(1) it hones in on population living in low income urban settings, in LMICs—a group both underserved in research and highly relevant to current global health priorities;<br>(2) it explicitly examines adaptation of interventions to these settings, rather than simply listing interventions; and<br>It therefore builds on but does not duplicate the earlier reviews, contributing to a planned series of complementary papers that map how delivery strategies vary across different marginalized populations and contexts.<br>We will update both the Introduction and Discussion to make these distinctions clearer, citing the two prior reviews directly and specifying how this study extends, rather than duplicates, their contributions.<br><br>C2: Editors comments on Title within the text<br>•Do you wish to focus on ID outbreaks or on all types of IDs? This question stems from the fact that IDs are ubiquitous whereas the concern of IDs with regard to rapid urbanization in LMICs is most often raised in the context of ID outbreaks because ID outbreaks are more likely to occur in urban localities of so-called LMICs than in urban localities of purported developed countries.<br><br>Focusing on ID outbreaks could also help the authors make a review differing from this 2025 scoping review (PMID: 39922209, DOI: 10.1016/S1473-3099(24)00744-8) and from this 2024 systematic review (SR; https://doi.org/10.1186/s13643-024-02651-9) especially since a SR is seen as a more useful form of evidence synthesis than a scoping review and the lag time required to update that SR may not have been reached yet (https://doi.org/10.1136/bmj.i3507). Also see https://doi.org/10.1038/s41579-021-00639-z. If you focus on ID outbreaks, then this should be specified throughout the manuscript (from the title to the end of the manuscript), not on IDs in general.<br><br>We thank the editor for this suggestion. To clarify, our review is not restricted to outbreaks but instead examines all infectious disease interventions delivered interpersonally, at scale, in urban poor settings in LMICs. This scope is already described in the Introduction (lines beginning: "This scoping review aims to enhance understanding of how infectious disease interventions can be adapted to urban poor settings…") and detailed in the Eligibility criteria and Table 1 – Type of intervention. The scope of interventions we include is consistent with that used in our earlier Epling et al. review, which examined delivery to racially, ethnically, and religiously marginalized populations.<br><br>2.Could you also specify, at least throughout the text (if not here as well), whether the interventions you wish to assess are mass/population-based public health interventions as it seems, in opposition to individual-level interventions? Based on Table 1, it seems you also wish to assess the outcomes of those interventions. However, this information does not appear in this title and in the introduction as expected.<br><br>Our focus is on mass population-level interventions – that include interpersonal delivery. We have revised lines 101 for better clarity to read: "Given our focus on the delivery context, we will concentrate on a subset of population-based interventions

characterized by one-on-one interactions…") and reiterated in Table 1 – Inclusion criteria under "Type of intervention."
As noted in Table 1 – Reported outcome and in the Objective section, we intend to extract information on outcomes reported (e.g., coverage, cost, prevalence, knowledge/attitudes). However, we will not be assessing interventions based on these results, as this falls outside the scope of a scoping review. A systematic review would be required for that level of synthesis, and at this stage we do not believe such evidence is available. This information will be used to report out on the types of outcomes measured – to inform the design of future studies.

3.As mentioned below, the term "poor" could be considered to be discriminative and I would remove it, especially since urban areas in LMICs most often have limited all-type resources. See BMJ Glob Health. 2022 Jun;7(6):e009704. doi: 10.1136/bmjgh-2022-009704.

We appreciate this important point and acknowledge the concern about terminology. To address it, we will replace "urban poor" with "low-income urban communities" throughout the manuscript. This phrasing avoids defining people by what they lack while remaining consistent with our scope. As described in our Methods (search strategy), our review includes related descriptors used by study authors, such as "slums," "informal settlements," and "street dwellers," to ensure comprehensiveness.

Editors comment on abstract
1.Conform to PLOS One author guidelines when formatting the revised manuscript. Accordingly, consider removing the Ethics statement in the abstract as well as sub-sections. Briefly, the abstract should look like in this article: https://doi.org/10.1371/journal.pone.0328645.

We agree and will reformat the abstract to align with PLOS ONE guidelines. Specifically, we will remove the subheadings and the ethics statement, and restructure the abstract to match the journal's style. Please see the revised abstract

Editor comments on Keywords
1.Why not just write «Urban» throughout the manuscript instead of «Urban poor»? Is it not possible to have a non-poor urban area in LMICs? See BMJ Glob Health. 2022 Jun;7(6):e009704. doi: 10.1136/bmjgh-2022-009704.

We appreciate this important point and acknowledge the concern about terminology. To address it, we will replace "urban poor" with "low-income urban communities" throughout the manuscript.

More specific comments from Editor
C1: Introduction: The introduction is currently unnecessarily very long. The greatest length of this manuscript should be reserved to the Materials and Methods section below.

We agree that the Introduction could be more concise and have revised it accordingly.

C2: Line 56-57: Could you complete this statement? Where do people moving to urban areas in LMICs come from? Or where did they come from? Rural areas of LMICs or from urban areas of high-income countries? What in their previous living areas drove them to urban areas of LMICs? Why did they not move to urban r rural areas of high-income countries?

We appreciate this thoughtful set of questions. The drivers of urban migration in LMICs vary widely by country and context. As this is the study protocol we have not yet extracted the information on setting. We therefore do not propose changes to the current text in the introduction, however we will ensure that our data extraction framework (see Box 1 and Data Extraction section) is designed to capture contextual details described in the included studies, including migration patterns and community-level characteristics. This approach will allow us to better reflect the nuances of where populations come from and why, based on empirical evidence.

C3: Line 62: I would complete this sentence with a statement like: «owing to limited

hygiene and therefore highly prone to the development of infectious outbreaks.» See https://doi.org/10.1038/s41579-021-00639-z

Drawing on the cover letter, the authors would also like to complete this statement with another one showing data about how rapid urbanization, increase in population density and the lack of health care infrastructures and resources drive the widespread of infectious diseases (IDs) in urban areas in LMICs.
We appreciate the suggestion. However, our review is not limited to outbreaks but is designed to capture the full range of infectious disease interventions delivered interpersonally in urban poor LMIC settings as explained above. The current text already highlights how rapid urbanization, high population density, and weak infrastructure create environments where infectious diseases thrive (see Introduction, second paragraph). To keep the Introduction concise (in line with recommendations made earlier) we prefer not to expand further.

C4: Line 63: Is this an urban area of a LMIC? If need be to take such an example alongside the above-suggested revisions, I would take the example of a prototypic highly populated urban area of a LMIC with increased ID burden (e.g., an urban area of Nigeria).

We appreciate this suggestion. We have removed these lines completely to ensure our introduction is concise as earlier suggested.

C5: Line66-67: Alongside the comment I made in line 62, this sentence would better be moved when presenting data about how rapid urbanization, increase in population density and the lack of health care infrastructures and resources drive the widespread of infectious diseases (IDs) in urban areas in LMICs. Once again, I suggest revising and completing this sentence to focus on urban areas of LMICs and on consequences of this fact with regard to the spread and control of infectious diseases.
This article may be helpful in this regard; https://doi.org/10.1038/s41579-021-00639-z

We thank the editor for this suggestion. As noted in our response to C3, our review is not restricted to outbreaks but covers a broader set of infectious disease interventions in urban poor LMIC settings. The current placement of this sentence in the Introduction is intended to show how rapid urbanization and weak infrastructure create conditions where infectious diseases thrive, thereby motivating the need for adapted interventions. While we recognize the value of framing in terms of outbreak vulnerability, shifting the focus in this way would change the scope of the review. We therefore propose to retain the current structure, while ensuring that our extraction framework captures how interventions were adapted in response to the full range of challenges faced in urban poor settings.

C6: It would also be great to end this comment with a few details about why the

I think this comment was cut off and we are unsure how to reply.

C7: While commenting on how urbanization in LMICs drives the spread of ID outbreaks, it would be great to mention whether and eventually why the problematic of ID control in rural areas of LMICs would be less relevant than that of ID control in urban areas of LMICs.
We thank the editor for raising this point. Most infectious disease control programs operate in rural areas, usually within existing primary health care units. However, our focus on urban poor settings does not imply that rural infectious disease control is less relevant. Rather, the delivery of interventions differs substantially between urban and rural contexts. Urban areas pose distinct challenges—including density, heterogeneity, and mobility—that require different forms of adaptation than those typically needed in rural settings. To keep the review focused and feasible, we have chosen to examine urban poor contexts, while recognizing that rural delivery remains equally important but outside the scope of this protocol.

C8: Line 69: If the authors implement the revisions suggested above, they would have highlighted how rapid urbanization with increase in population density and lack of

Powered by Editorial Manager® and ProduXion Manager® from Aries Systems Corporation

health care infrastructures and other resources in urban areas of LMICs drive and sustain the widespread of ID outbreaks in urban areas of LMICs at this level of the introduction. Accordingly, from line 69 to line 109, they would better focus on the current state of knowledge about interventions to control IDs in urban areas of LMICs in one paragraph, and on gaps in knowledge about interventions to control ID outbreaks in urban areas of LMICs in another paragraph. When presenting the current state of knowledge and gaps of knowledge, the authors would like to address issues such as whether the interventions are tailored to the context and culture of the researched populations and how much they contributed to the development and implementation of those interventions, including the role of community leaders and local researchers. While doing this, the authors
1.DOI: 10.1016/S1473-3099(24)00744-8
2.https://doi.org/10.1186/s13643-024-02651-9
These articles would greatly help the authors frame the needed text:
https://doi.org/10.1186/s13643-024-02651-9
https://doi.org/10.1136/bmjgh-2019-001855
Bayingana et al. Lancet Glob Health 2025; 13: e1627–35.
https://doi.org/10.1038/s41579-021-00639-z
https://doi.org/10.1371/journal.pone.0328645.
https://doi.org/10.1371/journal.pone.0317177

We thank the editor for this helpful suggestion. We agree that the Introduction could be strengthened by distinguishing more clearly between (1) what is already known about infectious disease interventions in urban LMIC contexts, and (2) the remaining gaps in knowledge. We have thus revised our third paragraph along those lines.

C9: Line 97: This is rather a scoping review protocol.

We agree with this clarification. The manuscript is indeed a protocol for a scoping review, not a completed review. This is already indicated in the title ("…a scoping review protocol") and reiterated in the Protocol Design section (line 120 onwards).
• In the Abstract, Materials and Methods we wrote:
"This scoping review will follow Joanna Briggs Institute (JBI) guidelines and the PRISMA methodology adapted for scoping reviews (PRISMA-ScR)."
• In the Protocol Design section we wrote:
"…we will conduct a scoping review using the Joanna Briggs Institute (JBI) guidelines [24] and utilize the PRISMA extension for scoping reviews (PRISMA-ScR) guidelines for reporting [25]."

C10: Line 111-117 (on objectives): Consider revising this text using a simple language (simple sentences). It seems to me that you would like to assess whether interventions used to control the spread of ID (outbreaks) so far in urban areas of LMICs were contextually and culturally appropriate or relevant. If this is the case, it should be clearly stated. You should also consider the rationale for conducting any scoping review (map the body of evidence on a given subject) when framing the aim of this scoping review protocol (not yet the scoping review). See
https://doi.org/10.1186/s12874-018-0611-x
https://doi.org/10.1371/journal.pone.0328645
https://doi.org/10.1371/journal.pone.0317177

We thank the reviewer for this observation. The objective of our review is not to assess the effectiveness of interventions in outbreaks, but rather to map how interventions have been adapted for urban poor LMIC settings. This is already reflected in our research questions (lines 98–103), but we agree the aim could be stated more clearly. We will revise the objective to emphasize that our review will (1) describe the types of adaptations reported and their relation to context, and (2) examine the methods and outcomes used to study these adaptations.

C11: Apart from the aim of the review, what is its overarching aim in terms of public/global health? What specific alert different from that sent by recent reviews on a similar topic do you wish to send to the Global Health community with the upcoming scoping review

We appreciate this question. Our overarching aim is to strengthen the evidence base

	on delivering infectious disease interventions equitably to marginalized populations. Whereas Epling et al. examined racial, ethnic, and religious marginalization, globally this protocol focuses on urban poor settings in LMICs, where marginalization is shaped by socioeconomic and environmental conditions. By mapping adaptations in this distinct context, our review complements earlier work and extends understanding of how delivery strategies must adapt to different populations.<br>We have summarized and added these lines into our manuscript.<br><br>C12: Line 121-126: I suggest moving this statement to lines 113 to 117 where you highlight the aim of the upcoming scoping review, along with my comment above.<br>We agree and have moved the objectives into a dedicated subsection to improve clarity and alignment with PLOS ONE formatting.<br><br>C13: Line 125-126: I understand that PLOS One recommends the authors upload the PRISMA-P checklist when submitting a scoping review protocol. However, I would like to inform the authors that the PRISMA-P checklist provided as S1 is for systematic review and meta-analysis protocols whereas this is a scoping review protocol. Given the use of the JBI and PRISMA-Scr guidelines for the upcoming scoping review, I suggest also using either of both guidelines for reporting this scoping review protocol.<br><br>Thank you for catching this. We have replaced the checklist with the PRISMA-ScR version.<br><br>C14: Line 126: I understand a scoping review protocol article may serve as registration record of a scoping review (https://www.icmje.org/recommendations/). However, did the authors register this protocol article or consider registering it? See<br>https://doi.org/10.1371/journal.pone.0328645<br>https://doi.org/10.1371/journal.pone.0317177<br><br>We thank the reviewer for this suggestion. In addition to publishing in PLOS ONE, we have registered the protocol on the Open Science Framework (OSF) to ensure transparency and accessibility, and have added the registration link to the Methods section.<br><br>C15: Line 128: I suggest deleting this sub-title.<br>We agree and have removed this sub-title to simplify the structure and improve flow.<br><br>C16: Line 129-130: This statement belongs to the first sub-section (Protocol design) of the Materials and methods section above.<br>We agree. We have moved this statement into the Protocol Design subsection to improve clarity and structure.<br><br>C17: Line 131: This sub-section should be termed “Eligibility criteria” and clearly describe inclusion and exclusion criteria in separate paragraphs.<br>We agree, and we have now included a section on eligibility criteria with most of the information presented in table format per your comment below.<br><br>C18: Line 135: Consider this sub-section after the Eligibility criteria sub-section.<br>We agree and have moved this subsection so that it follows directly after the Eligibility criteria. This reordering will improve the logical flow of the Methods section.<br><br>C18: Line 136: Consider replacing this statement with something like «onwards».<br>We have replaced the statement with onwards.<br><br>C19: Line 140: I insist is it necessary to mention the word «poor», stereotypic term which can contribute to the maintenance of social class inequalities?<br>See https://doi.org/10.1016/j.copsyc.2017.07.033<br>https://doi.org/10.1093/hrlr/ngab016<br><br>Please see response above, we have changed to use “low-income urban communities”<br><br>C20: Line 140-142: You need to provide refs here.<br>We have provided 3 references here

Powered by Editorial Manager® and ProduXion Manager® from Aries Systems Corporation

C21: Line 141-142: I understand there is no universal definition for spatial concepts or a definition specific to the so-called LMICs. However, you may consider proposing a new definition of spatial concepts based on knowledge from another group of countries such as European countries, especi ally since references 17 and 28 used to support the use of these terms (see line 142) do not seem to support the use of these search terms to refer to the term «urban». Accordingly, you could rather consider this literature, especially since there are also so-called LMICs in Europe: https://ec.europa.eu/regional_policy/sources/work/2014_01_new_urban.pdf or that from your country.

We thank the reviewer for this comment and their opinion. However, we decide to cite other articles that have used these terms in recent reviews.

C22: Line 143-151: Following the comment made just above, is this citation gaming? https://doi.org/10.1177/05390184221142218
We are unable to respond to this comment, because the lines cited do not have any citations.

C23: Line 143-151: Can you revise the preliminary search strategies presented in the S2 file and frame this sub-section as did these authors?
https://doi.org/10.1371/journal.pone.0328645
We have revised our search strategies to conform to the PRISMA-Search guidelines, which specify to "Include the search strategies for each database and information source, copied and pasted exactly as run". We have also framed the sub-section as much as we can to the style of the article shared.
Rethlefsen, M.L., Kirtley, S., Waffenschmidt, S. et al. PRISMA-S: an extension to the PRISMA Statement for Reporting Literature Searches in Systematic Reviews. Syst Rev 10, 39 (2021). https://doi.org/10.1186/s13643-020-01542-z

C24: Conform to search strategy guidelines:
http://dx.doi.org/10.1016/j.jclinepi.2016.01.021
DOI: dx.doi.org/10.5195/jmla.2018.283
Given that the key search concepts should emanate from the aim of the review, would you not consider these four terms as the key search concepts: ID, intervention, urban and LMICs and then develop each of those terms in the search strategies? Obviously, the LMICs mentioned in search strategies should be those of the LMIC classification chosen as highlighted when commenting on Table 1?
Thank you, we are unsure how to interpret this comment as it pertains to the provided DOIs: the first article is a guideline about peer reviewing search strategies which is recommended and not required for scoping reviews. The second article provides recommendations for searching but no requirements or guidelines. However, following your comment 23 above, we have updated the presentation of our search tables to conform to the PRISMA-S extension guidelines (see C23 above, and our S2 File).
Our PCC Framework also corresponds exactly with our search strategy: low-income urban communities (Search 2), infectious disease interventions (Search 1), and LMICs (Search 3).

C25: Line 152-164: As commented above, the sub-title should be revised to “Eligibility criteria” and the sub-section should be moved to line 128
We have made these changes as requested (See C17)

C26: Line 153-164: You get to choose whether you detail the inclusion and exclusion criteria in the text or in a table, not in both formats. Given that the table presentation appears to be more complete than the text, I would choose the table format.
Thank you for the guidance. We have summarized the inclusion and exclusion criteria description in the text to the barest minimum, and made reference to the table which is more detailed.

C27: Table 1: Have you provided all the socioeconomic and demographic characteristics of the population group to be included in the scoping review? I mean age limit, sex and gender, race, ethnicity, nationality, geographic origin, economic status
Beyond specifying that studies must focus on populations living in urban poor settings

Powered by Editorial Manager® and ProduXion Manager® from Aries Systems Corporation

	in LMICs, we have not imposed additional restrictions by age, sex, gender, race, ethnicity, or nationality. This is consistent with the exploratory nature of a scoping review and will allow us to capture the widest possible range of evidence. Where such characteristics are reported in the included studies, we will extract them as part of the data charting process (see Data Extraction section and Box 1).<br><br>C28: Table 1 (on concept): Did you wish to write "Concept" here?<br>We thank the reviewer for catching this error. Yes, this was a mistake, and we will correct the heading in Table 1 to read "Concept."<br><br>C29: Could you provide more details here about the type of (mass/population-based/public health as opposed to individual interventions which seem to be out of the scope of the upcoming review) interventions classified as preventive interventions, diagnostic interventions, and curative interventions? This means you would need three arrows for the variable "Intervention". For each type of intervention, you would then study the intervention modalities, how they were evaluated and how they were adapted to the context and the culture.<br>Thank you for this helpful comment. Our scope is population-level interventions that require interpersonal delivery. We agree it will be clearer if we explicitly subdivide "Intervention" into preventive, diagnostic, and curative categories. We will also extract (i) delivery modality, (ii) adaptation to context, and (iii) study methods.<br><br>C30: Table 1 (on evaluation): Do you wish to only assess how interventions were adapted and evaluated without studying the interventions themselves? Because it does not seem that you wish to do so when going through this table.<br>We apologize for the confusion. We have deleted this row.<br><br>C31: Table 1: (on outcome): Outcome of intervention is a variable that should be distinct from the intervention variable and so, be addressed in another arrow of the concept part of the PCC concept.<br>We apologize for the confusion regarding the formatting of the table. To clarify, intervention, information on adaptation, and reported outcomes are all distinct elements within the "Concept" domain. In the revised Table 1, we present them as separate rows under the broader PCC category of "Concept".<br><br>C32: Could you subdivide the outcome of intervention variable based on timeline (short-term, mid-term and long-term) and presence of absence of post-ID survival (survival outcomes versus hard outcomes [including quality of life, psychosocial well-being…)?<br><br>Please allow us to clarify, Table 1 presents our eligibility criteria, not our data extraction framework. For inclusion, studies must report at least one outcome, since our focus is on identifying interventions that have been delivered and adapted in practice. We will revise Table 1 to make this clearer.<br><br>C33: Table 1: on context: How will you ensure the readers believe that the countries included in the review are indeed LMICs? In other words, LMICs according to the World Bank or other classifiers? See BMJ Glob Health. 2022 Jun;7(6):e009704. doi: 10.1136/bmjgh-2022-009704. High-income countries based on which classification?<br>When finalizing the eligibility criteria and search strategy, we referred to The World Bank Classifications to determine which LMICs should be included. (please see ref 23). We have revised the text under objectives to reflect this.<br>World Bank. World Bank Country and Lending Groups. [cited March 25, 2025]. In: Knowledge Base > Country Classification [Internet]. Available from: https://datahelpdesk.worldbank.org/knowledgebase/articles/906519-world-bank-country-and-lending-groups<br><br>C34: Table 1: (under context): Do you make a difference between the arrow on countries and this one or did you wish to write "Study setting" rather than "Residence"?<br>We thank the reviewer for this comment. We have now revised this as Study Settings.<br><br>C35: Still regarding the setting, did you talk about the locality in a given country? Urban versus rural vs semi-urban? Bayingana et al. Lancet Glob Health 2025; 13: e1627–35. How will you define an urb an area? See for example how they defined an urb an are a

Powered by Editorial Manager® and ProduXion Manager® from Aries Systems Corporation

	in a so-called LMIC: https://doi.org/10.1016/j.jrurstud.2020.08.014.<br>We agree this is an important point. As noted in the Introduction (and in the reference you cite above), there is no universal definition of "urban." In our Methods (search strategy) we already specify that a wide range of terms were used (e.g., "slum," "informal settlement," "street dwellers"). To make this clearer, we have revised the Context description in Table 1/Eligibility criteria to state explicitly that studies were included if authors described the setting as urban using any of these terms.<br><br>C36: Table 1: Type of study: Did you wish to say: "Study design" and "Type of publication"? Consider reframing this box to make it readable. For instance, can a mixed-methods or a qualitative study be a quasi-experimental study? What is RCT? What is the "Case studies" study design? The authors also need to reflect on the study designs most likely to address the preventive, diagnostic and curative interventions they wish to assess when framing the text of this box.<br>Thank you for the comments, we agree. We meant Study design and Type of publication. We have revised the text to read: Studies that are peer-reviewed and published. Can be qualitative, quantitative or mixed methods, and also descriptive or analytic.<br><br>C37: Type of publication and study design should be addressed in different arrows of the context part of the PCC concept.<br>Please see comments C36, AND our revised table on how we have framed it<br><br>C38: Table 1 (on type of study): Why do you exclude these types of publication records/ is it not possible to include information from reviews, opinions, textbooks (e.g., chapters) and grey literature in a scoping review which ethymologically aims to map the body of evidence on a given subject? https://doi.org/10.1186/s12874-018-0611-x<br>We appreciate the reviewer's question and agree that scoping reviews can, in principle, include a broad range of evidence sources, including reviews, opinion pieces, textbooks, and grey literature. However, the aim of our review is more narrowly defined: to map how primary research studies have described the implementation and adaptation of infectious disease interventions in low-income urban communities. We also note that grey literature and non-empirical sources vary widely in quality and reporting detail, and a full appraisal of these materials is beyond the scope and resources of this review. We therefore restrict inclusion to original research articles that report empirical findings on interventions in these settings.<br>That said, we will consult relevant reviews and grey literature to ensure our search strategy is comprehensive and to contextualize findings, but they will not be included in the data charting or synthesis.<br><br>C39: Table 1 (on study subject): Consider deleting this arrow because the studied population is mentioned in the first arrow of this table and that is therefore the place where to specify that the review will focus on human subjects rather than animal subjects.<br>We agree, and have deleted the row<br><br>C39: Table 1: (on year of publication): Consider deleting this arrow. The information provided here belongs to "Information sources and search strategy" sub-section.<br>We agree, and have deleted the row<br><br>C40: Table 1: (language): Did you wish to specify "Publication language"?<br>Yes, thank you for catching this error. We have updated the manuscript.<br><br>C41: Line 168 (article screening): I am glad you will do this. Given that all authors are affiliated with institutions located in the USA where English language is the only official language, how are you going to ensure the accuracy of the information retrieved from publications in languages other than English? Consider specifying.<br><br>Our research team includes Portuguese, Arabic, French, and Spanish speakers and we have a wide network of colleagues that we can reach out to with other languages should they arise.<br>We recognize that positionality - including our own location and identities - can introduce risks of Anglocentric or Western lens bias, especially in screening and interpreting studies. It is therefore worth noting that our first author (MHO) is Nigerian

	living in the US, our last and corresponding author (MCB) was brought up in Peru and worked embedded in Ministries of Health in the S Pacific and Latin America, and a third author (IF) has lived experience in East Africa. This brings diversity of perspective to our study.<br><br>C42: Line 168: Consider renaming this sub-section «Study selection». Cn you make the text of this sub-section more understandable in a simpler language as in already published scoping review protocols? See https://doi.org/10.1371/journal.pone.0328645 https://doi.org/10.1371/journal.pone.0317177<br><br>C43: Line 171 (about pilot screenings): This sentence is difficult to understand for me<br>We have revised the text for better clarity<br><br>C44: Line 172 (about authors): Can you provide the full-spellings of these abbreviations<br>Those abbreviations are the author initials. However, we have removed them.<br><br>C45: Line 180: Why a PRISMA flow diagram? Will the review be a systematic or scoping one?<br>This is a scoping review, which also uses a PRISMA diagram - per JBI guidelines.<br><br>C46: Line 186-189: Could you better specify the variables (variables about study characteristics, variables about participant characteristics, and main review variables including the intervention variable [intervention, adaptation and evaluation of the intervention] and the outcome of intervention variable) whose data will be considered when extracting data from primary studies?<br>These are described in box 1. Specific nature of what is collected will depend on what is included in the published articles being reviewed.<br><br>C47: Line 191-192: Can you explain in more details how the ERIC and PRISM recommendations informed the conception of the data extraction process for the intervention variable and the outcome of intervention variable for this protocol article? I ask this question because apart from the fact that the upcoming review should be different from recent reviews on a similar topic, ERIC recommendations were made by experts from USA and Canada, but not by experts located in so-called LMICs. I do not know whether LMIC experts were involved in the recommendations recorded in ref 30 came from because I am un able to access its full-text. However, it seems to me that those recommendations would be useful mainly to synthesize data on the implementation of interventions and the evaluation of those interventions. It is difficult for me to visualize how ref 29 will be helpful here when going through it because it seems to be helpful for assessing the feasibility and relevance of public health interventions, but I missed in the text above information about whether you are willing to assess whether those interventions were feasible before their implementation and relevant, or at least it was not so clearly stated in the introduction and preceding sub-sections of this section of the manuscript.<br><br>We have clarified above how the topic of this review is different to what has been published.<br>As the aim of our paper is to describe how interventions were adapted, it is important for us to capture in detail the intervention descriptions in the articles that we review. In this protocol, we have drawn on both the ERIC (Expert Recommendations for Implementing Change) and PRISM (Practical, Robust Implementation and Sustainability Model) frameworks to guide the design of our data extraction.<br>We used ERIC as a reference to ensure our extraction captures not only what the intervention was but also how it was implemented and adapted to local context. For example, in Box 1 we include extraction items such as how communities were approached, which organizations led delivery, what coordination mechanisms were used, and what facilitators/barriers were described.<br><br>PRISM similarly has broad uptake in implementation science and guided our attention to contextual and outcome variables. It emphasizes how interventions interact with organizational, community, and system-level factors, and how these shape sustainability. Accordingly, our extraction of outcomes goes beyond effectiveness to include implementation outcomes (coverage, uptake, fidelity), sustainability outcomes

Powered by Editorial Manager® and ProduXion Manager® from Aries Systems Corporation

	(duration, maintenance of activities), and experiential outcomes (acceptability, perceptions).<br><br>C48: Box 1: I suggest renaming this title: «Information to be extracted for the interventions and outcome of interventions variables»<br>Thank you for this. We have revised the title to - Information to be extracted about the interventions.<br><br>C49: Box 1: Drawing on the box title proposed above, I suggest revising this box so that the information about the interventions variable (type implemented [preventive, diagnostic and curative], implemented interventions for each type, how each intervention was adapted, and how each intervention was evaluated) and about the outcome of interventions variable are presented separately and clearly.<br>We have revised the box accordingly to include the intervention type, delivery modalities, and to note that outcomes measured will be recorded.<br><br>C50: If this box was built based on information from reference 30, did you get permission to modify their figure/table? Even if you did not modify one of its figure/table, you would better specify in the footnote of this box that you built it based on reference 30.<br>It was informed by ERIC and PRISMA frameworks and then applied to this study, it is not a modification of a table or figure in those studies.<br><br>C51: Box 1 (effectiveness): To my knowledge, these are not bits of information about outcomes of interventions, but rather about the cost-effectiveness of those interventions. I am glad you would potentially like to include information about the cost-effectiveness of those interventions because this information would<br>We have revised the sub-head to read outcomes and measures<br><br>C52: Line 200-205 (analysis of results): Consider thoroughly revising this sub-section, specifying how the ERIC and PRISM guidelines would eventually help synthesize the upcoming scoping review. How will data be presented? Will you stratify any data? If yes, which ones and by which variable (e.g., world region)? In brief, see how other authors framed this sub-section: https://doi.org/10.1371/journal.pone.0322753<br><br>ERIC and PRISM were used to inform the design of the extraction framework only. We have thoroughly revised this section. Please see below<br>We have added a sub-section to the methods section describing in detail how we will analyze the data,.<br><br>C53: Line 207: Consider removing this sub-title.<br>We have removed the title and the section completely<br><br>C54: Line 208-212: This text belongs to the Discussion section which should be inserted after the Materials and Methods section, and should be further elaborated, describing the strengths and pitfalls of the methodology conceived for the upcoming scoping review. The authors would like to go through the previously provided scoping review protocol articles for inspiration on how to frame the discussion section.<br>We have removed the title and the section completely<br><br>C55: Line 214-221: Is this realistic? When are the implementation of the se arch strategy and the elimination of duplicates due to take place? Is this timeframe realistic?<br>We believe our timeframe is realistic. We were able to start the screening process based on feedback received in this review and are on track.<br><br>C56: Line 221: I suggest adding the sub-sections protocol deviation, data sharing and Ethical considerations, and Patient and public involvement at the end of the Materials and Methods section. See how the others did that:<br>https://doi.org/10.1371/journal.pone.0322753<br>https://doi.org/10.1371/journal.pone.0328645<br><br>These sections has been added now

	C57: Line 224: Before this statement and just after the Discussion section, consider adding a «Dissemination» statement as did these authors: https://doi.org/10.1371/journal.pone.0328645<br>This section has been added<br><br>C58: References: Strengthening and update will increase the relevance of this protocol.<br>We have updated the references.<br><br>Reviewer 1:<br>C1: Abstract needs minor improvements; “Materials and methods” section is slightly dense; simplifying sentences (e.g., breaking long lists of steps) would improve readability.<br>Thanks for these comments. We have significantly revised the abstract and methods<br><br>C2: The conclusion could be more specific by emphasizing the expected contribution of the scoping review (e.g., identifying adaptation strategies, informing future program design).<br>Thanks for these comments. We have now revised the conclusion as suggested<br><br>C3: Consider consistency in terminology: “urban poor setting” could be pluralized to “urban poor settings” to match the rest of the manuscript.<br>Following revisions proposed by the editor, we have replaced the term “urban poor” with “low income urban communities” through the text<br><br>C4: Some sentences are long and could be simplified for clarity. For example, the sentence beginning line 60 “The rapid pace and scale of urbanization…” could be broken into two.<br>Thank you – we have further edited the text.<br><br>C5: Minor typographical issues: “leisMHOaniasis” line 80 appears to be a typo.<br>Thank you for catching this - we have revised it.<br><br>C6: The aim and objectives are currently somewhat broad and overlapping with background information, which makes them less clear. The aim uses vague language (“to understand what has been published”) and would benefit from being more action-oriented, for example, “to map and synthesize evidence on adaptations of infectious disease interventions in urban poor settings in LMICs.” The objectives should flow directly from the aim and clearly reflect the research questions, specifying the population, setting, and type of interventions studied. Reframing the objectives to explicitly address (1) intervention adaptations in relation to context and (2) study designs, methods, and outcomes would improve clarity, specificity, and alignment with the research questions<br>Thank you for these comments. We have carefully reworded the aim, and objective of the review. Please see our new section on "Aims and objective of the scoping review”. This has helped clarify our own thinking.<br><br>C7: Some sentences are long and could be simplified for clarity, particularly in the “Information sources and search strategy” and “Data Extraction” sections. Breaking these into shorter sentences would improve readability.<br>Thank you – we have further edited the text.<br><br>C8: The use of the PCC (Population, Concept, Context) framework is suitable, but the description could be slightly more concise. For example, the “concept” could clearly state it focuses on interpersonal delivery adaptations rather than listing examples at this stage.<br>We have revised this section to ensure clarity. Please see our section on “Protocol design”.<br><br>C9: The rationale for selecting 2000 as the start year is not explicitly stated; adding a brief justification would be helpful.<br>We selected 2000 as the start year to ensure the review captures evidence from a time frame that is both relevant to current global health delivery models and feasible for review. Many large-scale infectious disease control initiatives in LMICs—including

	global immunization campaigns, scale-up of HIV/TB programs, and mass drug administration for NTDs—expanded significantly after 2000, often with increased attention to delivery in urban contexts.<br>We have now added this line under our inclusion criteria.<br><br>C10: Consider providing more detail on how search results will be managed across multiple languages, especially for non-English studies.<br><br>Thank you for the opportunity to clarify. As noted, we will not exclude studies based on language at this stage. If we have language capacity on our team, studies will be read in the original language. Where this is not possible, we will use translation software (e.g., Google Translate) to produce an English version and ask someone proficient in the language to compare the translation with the original to confirm accuracy. If this is not possible, we will document the exclusion of the study and report it transparently in the review.<br><br>C11: The table is detailed and clear. However, there is a minor inconsistency: in the text, the authors state that grey literature will be included, page 9, line 160, but in the table, grey literature is listed under exclusion. Clarification is needed.<br>Thank you for this observation. We have corrected line 160, as grey literature will not be included.<br><br>C12: The criteria could also briefly explain how mixed interventions or multi-component studies will be handled.<br>In mixed interventions or multi-component study information on each intervention and component will be collected.We have now added this text under our Analysis section<br><br>C13: Box 1 is detailed but long; consider summarizing key elements in the main text and moving detailed subpoints to an appendix.<br>We have revised this box also following comments from the editor to add more detail and would prefer to retain it within the main file. We believe pulling out the detail of interventions and how they were implemented is critical in implementation science.<br><br>C14: Clarify whether any software or tools will be used to manage qualitative synthesis (e.g., NVivo, MAXQDA) or if this will be entirely manual.<br>Thanks for this comment. Qualitative synthesis will be managed manually and supported by AI. We have added more detail on how this will be done to the sub-section on analysis under results.<br><br>C15; The timeline is realistic, but adding anticipated challenges or mitigation strategies (e.g., delays in full-text retrieval or translation) could strengthen planning transparency.<br>We appreciate this thought. However we have been able to start the screening, implementing feedback received in this review, and we believe we are on track. If our timings change we will report.<br><br>C16; Some citations are repeated or appear as placeholders (e.g., “[17, 17,28]”); these should be checked for accuracy, line 142.<br>We have also revised this. Thanks for catching this error.<br><br>C17: One minor point: the funding statement contains a typographical error (“No was received” should likely read “No funding was received”). Otherwise, this section is clear and adequate.<br>Thanks for catching this error. We have now revised it<br><br>C18: Minor language, typographical, and consistency issues should be corrected.<br>We have carefully revised the manuscript and corrected typographical errors.<br><br>C19: Emphasizing action-oriented language in the objective will strengthen the manuscript’s clarity and alignment with scoping review methodology.<br>Thank you, we have revised this and think it is now stronger.<br><br>C20: Please check your references and be consistent<br>We have carefully revised the manuscript and addressed this.

	C21: Line 97: The aim and objectives are currently written in a way that is somewhat wordy and overlaps with background information. Ideally, the aim should be a clear, concise statement of the overall purpose of the review, separate from the context<br>We have carefully revised the manuscript and addressed this.<br><br>C22: Line 97; The aim uses the phrase “to understand what has been published,” which is vague and passive. For a scoping review, it is better to use action-oriented language such as “to map,” “to synthesize,” or “to describe.”<br>We have carefully revised the manuscript and addressed this. Please see our comments above on this.<br><br>C23: Line 113: The objectives should flow naturally from the aim and clearly reflect the research questions. Currently, there is a slight disconnect: the aim emphasizes understanding what has been published, whereas the objectives focus on intervention adaptations, contextual factors, methods, and outcomes. Please align the objectives to your research question<br>We have carefully revised the manuscript and addressed this. Please see our comments above on this.<br><br>C24: Line 114: The objectives could be more specific regarding the population (urban poor), setting (LMICs), and type of interventions (interpersonal delivery-focused interventions). This specificity will make the aim and objectives sharper and more actionable. Be clear with your scope<br>Thank you. We have carefully revised the manuscript and addressed this.<br><br>C25; Line 114: Why re you writing research questions mixed with research objectives? Reframe the aim to a concise, action-oriented statement. Reframe the objectives to explicitly reflect the research questions.<br>We have carefully revised the manuscript and addressed this.<br><br>C26: Line 166. Table 1: This PCC table is too broad, make it concise<br>We have carefully revised the manuscript and addressed this.
**Additional Information:**	
**Question**	**Response**
**Financial Disclosure**<br><br>Enter a financial disclosure statement that describes the sources of funding for the work included in this submission. Review the submission guidelines for detailed requirements. View published research articles from *PLOS ONE* for specific examples.<br><br>This statement is required for submission and **will appear in the published article** if the submission is accepted. Please make sure it is accurate.	The author(s) received no specific funding for this work.

**Funded studies**<br>Enter a statement with the following details:<br>• Initials of the authors who received each award<br>• Grant numbers awarded to each author<br>• The full name of each funder<br>• URL of each funder website<br>• Did the sponsors or funders play any role in the study design, data collection and analysis, decision to publish, or preparation of the manuscript?<br><br>Did you receive funding for this work?	
**Competing Interests**<br><br>Use the instructions below to enter a competing interest statement for this submission. On behalf of all authors, disclose any competing interests that could be perceived to bias this work—acknowledging all financial support and any other relevant financial or non-financial competing interests.<br><br>This statement is required for submission and **will appear in the published article** if the submission is accepted. Please make sure it is accurate and that any funding sources listed in your Funding Information later in the submission form are also declared in your Financial Disclosure statement.<br><br>View published research articles from *PLOS ONE* for specific examples.	The authors have declared that no competing interests exist.

**NO authors have competing interests**<br><br>Enter: *The authors have declared that no competing interests exist.*<br><br>**Authors with competing interests**<br><br>Enter competing interest details beginning with this statement:<br><br>*I have read the journal's policy and the authors of this manuscript have the following competing interests: [insert competing interests here]*<br><br>* typeset	
**Ethics Statement**<br><br>Enter an ethics statement for this submission. This statement is required if the study involved:<br><br>• Human participants<br>• Human specimens or tissue<br>• Vertebrate animals or cephalopods<br>• Vertebrate embryos or tissues<br>• Field research<br><br>Write "N/A" if the submission does not require an ethics statement.<br><br>General guidance is provided below. Consult the submission guidelines for detailed instructions. **Make sure that all information entered here is included in the Methods section of the manuscript.**	Ethics approval will not be required. Patients nor the public will not be involved at any stage of this scoping review.

Powered by Editorial Manager® and ProduXion Manager® from Aries Systems Corporation

**Format for specific study types**<br><br>**Human Subject Research (involving human participants and/or tissue)**<br>• Give the name of the institutional review board or ethics committee that approved the study<br>• Include the approval number and/or a statement indicating approval of this research<br>• Indicate the form of consent obtained (written/oral) or the reason that consent was not obtained (e.g. the data were analyzed anonymously)<br><br>**Animal Research (involving vertebrate animals, embryos or tissues)**<br>• Provide the name of the Institutional Animal Care and Use Committee (IACUC) or other relevant ethics board that reviewed the study protocol, and indicate whether they approved this research or granted a formal waiver of ethical approval<br>• Include an approval number if one was obtained<br>• If the study involved *non-human primates*, add *additional details* about animal welfare and steps taken to ameliorate suffering<br>• If anesthesia, euthanasia, or any kind of animal sacrifice is part of the study, include briefly which substances and/or methods were applied<br><br>**Field Research**<br><br>Include the following details if this study involves the collection of plant, animal, or other materials from a natural setting:<br>• Field permit number<br>• Name of the institution or relevant body that granted permission	
**Data Availability**<br><br>Authors are required to make the data underlying their research findings fully available, without restriction.<br><br>If the manuscript reports pilot or preliminary results, the data underlying those results must be made available at the time of publication. A Data Availability	N/A - No results are reported

Powered by Editorial Manager® and ProduXion Manager® from Aries Systems Corporation

Statement describing where the data can be found is required at submission. Your answers to this question constitute the Data Availability Statement and will be published in the article, if accepted. When legal or ethical requirements prohibit public data sharing, authors must indicate how researchers may obtain access to the data. See the PLOS Data Policy and FAQ for detailed information.<br><br>**Important:** Stating 'data available on request from the author' is not sufficient. If your data are only available upon request, select 'No' for the first question and explain your situation in the text box.<br><br>For protocols without pilot or preliminary data, authors are strongly encouraged to state how they plan to share research data from their study when it is completed or published.<br><br>If your study includes pilot data, will they be made fully available without restriction?	
**Describe where the data may be found in full sentences. If you are copying our sample text, replace any instances of XXX with the appropriate details.**<br><br>• If **no pilot data are reported** in the manuscript, please enter the following: *No datasets were generated or analysed during the current study. All relevant data from this study will be made available upon study completion.*<br>• If the manuscript reports pilot data **held in a public repository**, include URLs, accession numbers or DOIs. If this information will only be available after acceptance, indicate this by ticking the box below. For example: *All XXX files are available from the XXX database (accession number(s) XXX, XXX.).*<br>• If the manuscript reports pilot data contained **within the manuscript and/or**	No datasets were generated or analysed during the current study. All relevant data from this study will be made available upon study completion.

**Supporting Information files**, enter the following:<br>*All relevant data are within the manuscript and its Supporting Information files.*<br>• If neither of these applies but you are able to provide **details of access elsewhere**, with or without limitations, please do so. For example:<br><br>*Data cannot be shared publicly because of [XXX]. Data are available from the XXX Institutional Data Access / Ethics Committee (contact via XXX) for researchers who meet the criteria for access to confidential data.*<br><br>*The data underlying the results presented in the study are available from (include the name of the third party and contact information or URL).*<br>• This text is appropriate if the data are owned by a third party and authors do not have permission to share the data.<br><br>For study protocols with data management or sharing plans, authors are encouraged to briefly describe in their Data Availability Statement how data they generate will be made accessible when the study is completed. For example:<br>• Deidentified research data will be made publicly available when the study is completed and published.<br><br>* typeset	
Additional data availability information:	

Powered by Editorial Manager® and ProduXion Manager® from Aries Systems Corporation

Editor-in-Chief
PLoS ONE
06 October 2025

RE-SUBMISSION OF ORIGINAL RESEARCH ARTICLE FOR PUBLICATION IN YOUR JOURNAL

We are pleased to re-submit our manuscript titled **“Exploring how infectious disease control interventions are delivered in low-income urban communities in low- and middle-income countries: a scoping review protocol”** for your consideration for publication in PLoS ONE.

Rapid urbanization in low- and middle-income countries has intensified the challenges of infectious disease control in densely populated, under-resourced urban poor communities. These settings often face severe gaps in infrastructure, sanitation, and access to health services, creating conditions where infectious diseases can spread rapidly and remain endemic. Yet, the literature remains fragmented on how interventions are being effectively adapted to meet the unique needs of these environments.

Here we present a revised manuscript following the comments from the editor and reviewers. Our protocol itemizes the plan to provide evidence and actionable insights into adaptation strategies, implementation outcomes, and the contextual facilitators and barriers that shape success.

We believe this review protocol is relevant to your journal’s readership given the growing urban health disparities and the global push for inclusive, equitable public health strategies. This protocol will be valuable not only to researchers and implementers, but also to policymakers aiming to design responsive and effective infectious disease control programs in urbanizing LMICs.

We confirm that this manuscript has not been published elsewhere and is not under consideration by any other publication. All authors have reviewed and approved the manuscript for submission.

Thank you for considering our work. We hope you will find this version worthy of publishing .

Sincerely,
Hammed O. Mogaji, PhD (Corresponding Author)
On behalf of all co-authors
Public Health Unit, Department of Applied and Behavioral Sciences,
College of Arts and Sciences, Marian University, USA

Manuscript

Click here to access/download;Manuscript;manuscript_clean copy_Oct 6 2025.docx

Exploring how infectious diseases control interventions are delivered in low-income urban communities in low- and middle-income countries: a scoping review protocol

Mogaji, H.O.[1], Fatah, I[2], Dorris, C.S[3], and Baker, M.C[4*]

[1]Department of Behavioral and Applied Sciences, Marian University, Indianapolis, Indiana, United States of America;

[2] Georgetown University, Washington, United States of America;

[3] Dahlgren Memorial Library, Georgetown University, Washington, United States of America;

[4]Department of Global Health, Georgetown University, Washington, United States of America;

Corresponding author*: **Email:** mcb93@georgetown.edu; **Address**: Department of Global Health, Georgetown University, Washington, United States of America

Mogaji, H.O: mogajihammed@gmail.com

Fatah, I: iaf4@georgetown.edu

Dorris, C.S: csd24@georgetown.edu

Baker, M.C: mcb93@georgetown.edu

Abstract

Infectious diseases thrive in densely populated low-income urban communities where living conditions, hygiene, and access to health services are sub-optimal. Information on how to adapt infectious diseases control interventions to be effective in these settings is limited. The proposed scoping review will follow Joanna Briggs Institute (JBI) guidelines and the PRISMA methodology adapted for scoping reviews (PRISMA-ScR). Research questions were created using the Person, Concept, Context (PCC) framework. In this study person refers to low-income urban communities, concept refers to community-based infectious disease interventions adapted to their setting, and the context is low- and middle-income countries. Relevant papers published after 2000 will be retrieved using a search strategy that incorporates all the PCC topics. Searches will be made in four databases: MEDLINE, Embase, Global Health (all via Ovid), and Web of Science. Duplicates will be identified and removed with EndNote 20, and all articles will be uploaded into Covidence for screening in two stages using pre-defined inclusion and exclusion criteria. Quantitative and qualitative data will be extracted into Excel spreadsheets and analyzed. Our review will identify different approaches that have been taken to adapt infectious disease interventions in low-income urban communities of LMICs that can be further tested in future programs and studies. We will also highlight methods that have been used to study such interventions in their context.

Keywords:

Infectious diseases, Urban poor, Low Middle-Income Countries, Interpersonal Interventions

Introduction

Urbanization has accelerated rapidly in this century, with approximately 57% of the world's population now living in cities [1]. Furthermore, the global urban population is projected to reach 6.25 billion by 2050 [2]. This growth is driven by multiple factors, including government policies on public housing, land use, property ownership, and economic opportunities [3, 4]. To date, the largest expansion of urban populations has been focused on low and middle-income countries (LMICs) [5]. This rapid pace and scale of urbanization has resulted in significant pressure on available infrastructure and resources, with expansion into areas previously considered uninhabitable, often referred to as informal settlements or slums and are characterized by inadequate housing conditions, poor health and safety, and fewer work opportunities [4,6,7].

The impact of urbanization on the spread of infectious diseases is highly contextualized. On one hand, urbanization can reduce mortality and morbidity caused by infectious agents in areas where living conditions, hygiene, and access to health services are optimal [8]. On the other hand, rapid urbanization has also resulted in the growth of informal settlements [9], where infectious diseases thrive. Factors associated with the spread of infectious diseases in urban areas, especially low-income urban communities, have been summarized by Alirol *et al* [8] and Neiderud *et al* [10]. High population density allows air-borne infections like influenza, measles, and *Mycobacterium tuberculosis* to rapidly spread [11]. Inadequate water and sanitation services contribute to diarrhoeal diseases caused by cholera and *E. coli* [12,13], to the transmission of soil-transmitted helminthiasis and other intestinal parasites [14], and to the proliferation of rodents such as rats which can carry *Leptospira interrogans* [15]. Accumulated waste can provide breeding sites for vectors like sand flies that carry parasites causing leishmaniasis and of *Aedes* mosquitoes that are vectors for dengue, yellow fever, and chikungunya [16].

While many interventions have proven to be effective in preventing infectious diseases [17], implementation failure, including in urban settings, has also been documented. For example, immunization programs have reported challenges in reaching urban dwellers due to working with large populations that are transient in nature - with inconsistent employment patterns, and

multicultural beliefs and practices [18]. Similarly, mass drug administrations (MDA) for controlling neglected tropical diseases (NTDs) in Africa and the Americas, have had lower reported coverage in urban settings due to the heterogeneity that exists within urban environments [19,20]. The success of immunization, contraception, and antenatal care services was also reported to be sub-optimal in urban slum areas of Chandigarh in the North of India. [18, 21]. Community health interventions in urban settings, especially, in low-income communities, must therefore contend with population mobility, informal housing, and cultural diversity, which together add complexity to the delivery of services.

These persistent challenges highlight the need to understand how interventions are being adapted in practice. Yet, despite this recognition, there is limited synthesized evidence on adaptations to low-income urban communities in LMICs. A recent review begun to address related questions by mapping the types of community-based interventions delivered in informal urban settlements, however they stopped short of providing insight on how interventions were adapted to this setting [17]. These gaps demonstrate the need for a review that specifically identifies approaches taken to adapting interventions and how these were studied.

This review will address that gap – across multiple infectious diseases - by concentrating on a subset of population-based mass interventions characterized by including one-on-one interactions between a distributor and a recipient within the community. This delivery mode, often referred to as ‘campaigns’, is commonly used for vaccinations, mass drug administration (MDA), screening and test-and-treat initiatives, and distribution of products such as bed nets, condoms, and facemasks. This focus aligns with the methodological approach taken in a previous scoping review focused on a different marginalized population, namely ethnically, racially, and religiously marginalized populations. [22]

Aim and objectives of the review

The aim of this scoping review is to map and synthesize evidence on how infectious disease interventions have been adapted for delivery in low-income urban communities in LMICs. In line with this aim, our objectives are to: (1) catalogue the types of adaptations reported; (2) examine how these adaptations relate to context; (3) assess the extent and distribution of the available evidence; and (4) review the study designs and methods used to investigate these adaptations.

Materials and Methods

Protocol design

Scoping reviews use standardized methods, taking a systematic approach, and are the methodology of choice when the aim is to get a broad overview of a potentially diverse body of literature [24]. This protocol documents how we will conduct a scoping review using the Joanna Briggs Institute (JBI) guidelines [24], and the Preferred Reporting Items for Systematic Review and Meta-Analysis extension for Scoping Reviews (PRISMA-ScR) guidelines [25]. This protocol has also been registered on the Open Science Framework (OSF), accessible here https://doi.org/10.17605/OSF.IO/T8EWA. The PRISMA ScR checklist, completed for this study, has been included as Supplementary File 1.

In this review we used the Person, Concept, Context (PCC) framework to guide the formation of research questions and search strategies [26]. Our population will include those living in low-income urban communities. The concept will be adaptations of infectious disease interventions characterized by one-on-one interactions between a distributor and a recipient within the community. The context will be low-middle-income countries as defined by the World Bank [27].

Eligibility Criteria

Our inclusion and exclusion criteria is detailed in Table 1. We consider studies published from 2000 relevant because many large-scale infectious disease control initiatives in LMICs - including global immunization campaigns, scale-up of HIV/TB programs, and mass drug administration for NTDs - expanded significantly after 2000, often with increased attention to delivery in urban

contexts. We exclude studies based in rural areas, not because these studies are less important, but to align with our stated objective.

Table 1: Inclusion and exclusion criteria

**Framework**	**Items**	**Inclusion**	**Exclusion**
Population	Communities	Studies concerning low-income urban communities.	High income urban communities and rural communities.
Concept	Type of intervention	Studies on interventions that require interpersonal contact in a community setting, specifically: immunizations, mass drug administration, deworming, screening, contact tracing, and distribution of commodities (e.g. condoms, facemasks, bed nets).	Studies on interventions that do not rely on one-one interaction. This includes interventions reaching many people simultaneously with no interpersonal interaction in the delivery (e.g., mass communication and health education campaigns) and interventions that rely on environmental modifications (e.g., spraying for vector control or water and sanitation projects).<br>Interventions delivered exclusively in facility settings.
Concept	Information on adaptation	Studies that describe how the intervention was adapted to low-income urban communities, even if very limited.	Studies that did not describe how the intervention was adapted to low-income urban communities.
Concept	Reported outcome	Studies must report at least one outcome (e.g., implementation, clinical/epidemiologic, economic, or experiential) indicating that the intervention was delivered.	Studies that did not report any implementation or intervention outcome.

Context	Countries	Studies implemented in Low- and Middle-Income Countries (LMICs).	Studies implemented in High Income Countries (HICs).
Context	Study design and type of publication	Research studies that are peer-reviewed and published. Can be qualitative, quantitative or mixed methods, descriptive or analytic, and primary or secondary research.	Literature reviews, textbooks, dissertations, editorials, opinion pieces, and grey literature.
Context	Publication language	Studies reported in all languages.	

Information sources and search strategy

Relevant articles published from the year 2000 onwards until the date of search will be retrieved from MEDLINE, Embase, Global Health (all via Ovid), and Web of Science with the goal to identify articles from both medical and social sciences fields. The search strategy incorporates both database-specific subject headings and keywords that represent all three PCC topics: low-income urban communities, infectious disease interventions, and LMICs. As there is no universally accepted global definition of urban, we will use search terms like “urban,” “peri-urban,” “city,” “municipality,” “urbanization,” and “township.” used in other literature reviews on this subject. [17,28]. Terms related to low-income urban communities including slums, informal settlements, street dwellers, are also used to select for the population of interest. As our concept focuses on mass infectious disease interventions that require interpersonal interaction between those delivering a health product and those receiving it, we included search terms related to vaccine, mass drug administration, and mass screening or testing for infectious diseases. The search strategy for Ovid MEDLINE is shown in [see S2 File]. The search strategy was developed by a university librarian specialist and peer-reviewed with the other authors, then tested to obtain an optimal balance between allowing for a broad scope and feasibility. These search terms were subsequently translated for the remaining databases.

Study selection

All retrieved articles will be uploaded into Covidence Software for screening. Duplicates will be identified and removed with EndNote 20 (Clarivate Analytics, Philadelphia, PA). Article screening will be performed by four trained research assistants. In the first phase, titles and abstracts of at least 200 articles will each be independently reviewed by two researchers - until an inter-rater reliability of 95% agreement is achieved. Discrepancies will be resolved through discussion. Once this threshold is reached, screening will proceed with one researcher per article. Articles included in the title and abstract stage of screening will then undergo full-text review, with the same eligibility criteria used to determine relevance. A PRISMA flow diagram will be created to summarize the results of the search and reasons for exclusion. Reviewers will continue to meet every 1-2 weeks to discuss the studies, including any articles for which there is uncertainty on whether to include it.

Data Extraction

The data extraction process will be conducted by two researchers who will extract data from assigned articles. This review will be checked by a second researcher. A standardized data extraction form in Microsoft Excel will be used to capture both quantitative and qualitative information. The extraction form will include information on the topics presented in ~~Table 2~~ as well as bibliographic information (including author, year of publication, and language) and information on methods and outcomes. The extraction framework was developed with reference to the Expert Recommendations for Implementing Change (ERIC) framework [29] and the Practical, Robust Implementation and Sustainability Model (PRISM) [30], both of which have been widely applied in implementation research.

Box 1: Information to be extracted about the interventions and context

Intervention:

- What the intervention is e.g., bed net distribution, health education campaign, screening
- What the intervention type is e.g. preventive, diagnostic, curative
- Why the intervention was needed
- Intervention goal, including disease(s) targeted

Context:

- Where implemented: Countries, sub-country, number of sites
- Community setting environment: socio- economic, geography, political
- Population receiving intervention

Implementation and adaptation made to the context:

- Delivery modalities with explanation on why selected including point of delivery and timing
- How the community was initially approached and how they were engaged during each stage of the study
- What organization(s) led delivery of the intervention and the roles of staff within these organizations
- Other stakeholders and their role
- Processes put in place to coordinate between all stakeholders
- Adoption of intervention (e.g., number of workshops, amount of product delivered) and fidelity to the original plan
- How decisions were made related to the context
- Facilitators: what worked well and why
- Barriers: what did not work, why, and how these were overcome
- Sustainability: how long were the interventions delivered f
- Costs and funding

Analysis of results

We will analyze data using both descriptive summaries and thematic narrative analysis. For descriptive summaries we will present tables summarizing basic study characteristics: country (and region), year of publication, intervention type, type of urban setting (e.g., informal settlement vs other), and population demographics. All elements of mixed or multi-component studies will be extracted. We will not attempt meta-analysis or effectiveness estimates as this review focuses on identifying how interventions have been adapted and how these adaptations have been studied.

Thematic analysis will focus on how interventions were adapted; what was reported to work and not work; barriers and facilitators; how stakeholders were engaged; context-driven decisions; and the socio-economic, geographic, and political variables that shaped adaptation. Analysis will be qualitative and conducted by the two senior researchers. All textual data will first be carefully reviewed and emerging themes identified. Additional columns will then be created in the extraction spreadsheet, and thematic labels (codes) manually entered. Results will be synthesized within each theme, with a detailed description of common trends as well as notable exceptions. To complement researcher perspectives, ChatGPT will be queried to identify potential additional themes and to further explore the data within each theme. Any information provided by ChatGPT will be manually cross-checked against the original data sources before inclusion.

Status and timeline of the study

The scoping review will be conducted over a six-month period, from August 2025 to January 2026. Development of the search strategy will take place in August, followed by the initial screening of titles and abstracts within the same month. Full-text screening will occur in September, followed by data extraction in October. Data analysis and interpretation will take place in November.

Protocol deviation

Any deviations from the described protocol will be documented and transparently reported in future publications and presentations of study results. The research team will ensure that any deviations are justified, minimized, and do not compromise the integrity or objectives of the study.

Data-sharing

In line with open science principles, we will make the final data extraction sheet openly available through a public repository such as the Open Science Framework.

Ethical considerations

Ethical approval was not required because the study does not include participants. However, this study is in accordance with the Declaration of Helsinki [31] and Ethical Guidelines for Nursing Research in the Nordic Countries [32].

Patient and public involvement

None

Discussion

Our overarching aim in this review is to map and synthesize evidence on how community-based infectious disease interventions have been adapted for delivery in low-income urban communities in LMICs. The findings will enhance understanding of how such interventions might be adapted for these communities and how adaptations relate to context. The review will highlight key facilitators and barriers to implementing adaptations as well as methods used to study these - providing valuable insights to guide future research and practice in similar contexts.

The strengths of this review include its methodological rigor, with procedures designed to minimize selection bias. These are reinforced by the authors' prior experience conducting a related review among other marginalized groups [22], collaboration with a senior trained librarian, and the inclusion of articles published in multiple languages, supported by the team's diverse linguistic capabilities. We also employed a pilot-screening approach to strengthen selection procedures, drew upon the team's extensive experience delivering infectious disease interventions in LMICs across three world regions, and applied established frameworks for data extraction and analysis.

A potential limitation of our review is the availability of studies that meet our inclusion criteria. The delivery of infectious disease interventions is usually led by health program implementers and frontline workers, with relatively few academic researchers engaged in sustained collaboration with these groups. More broadly, implementation research - and the networks linking academics with program practitioners - remains a relatively nascent field. As a result, the pool of eligible studies may be limited; however, documenting this gap will itself be an important contribution.

Dissemination plan

The results from the scoping review will be submitted for publication in a peer-reviewed journal also be prepared for submission to conferences.

Conclusion

Our review will provide critical insights into approaches used to adapt infectious disease interventions for marginalized populations in LMIC low-income urban communities. By identifying effective strategies and highlighting contextual facilitators and barriers, the study will inform both future research and innovative program design. Ultimately, this work will contribute to strengthening the evidence base for sustainable, culturally appropriate, and impactful infectious disease interventions in resource-constrained urban settings.

Supplementary Information

S1 File: PRISMA ScR Checklist

S2 File: Search terminology

Declarations

Authors' contributions

MBC conceptualized the study; MCB, HOM, IF prepared the protocol; MCB, HOM, CSD designed the search strategy; CSD constructed the search framework; HOM, IF, and MCB prepared the first version of the manuscript. All authors revised and approved the manuscript.

Ethics approval and consent to participate

Ethics approval will not be required. Patients nor the public will not be involved at any stage of this scoping review.

Consent for publication

Not applicable

Availability of data and materials

No data has been collected yet. Upon completion of the review the final extraction sheet will be provided as a supplementary file.

Funding

No funding was received for this study.

References

1. World Bank Group. World Urbanization Prospects, United Nations. https://data.worldbank.org/indicator/SP.URB.TOTL.IN.ZS

2. United Nations Department of Economic and Social Affairs. 2014 revision of the World Urbanization Prospects. https://www.un.org/en/development/desa/publications/2014-revision-world-urbanization-prospects.html

3. Yu D, Li X, Yu J, Shi X, Liu P, Tian P. Whether Urbanization Has Intensified the Spread of Infectious Diseases—Renewed Question by the COVID-19 Pandemic. *Front Public Health*. 2021;9. doi: 10.3389/fpubh.2021.699710.

4. World Bank Group. Urban Development. https://www.worldbank.org/en/topic/urbandevelopment/overview

5. Gunaratna, KL, Eduardo Cesar LM, Yansui L, Liana R, Akiko O, Obudho R, Berma M, Sumanm KK, Jan Q, Harrison P, Maharaj B, Knowles C, Sandro G, Seto KC, Ndilila FM. IAP Statement on Implications of Urbanization in Low- and- Middle- Income Countries. The Inter Academy Partnership, 2022.

6. United Nations. Rapid urbanization and population growth are outpacing the construction of adequate and affordable housing. https://unstats.un.org/sdgs/report/2019/goal-11/

7. Cociña, C and Landesman, T. Better cities are possible: transforming informal settlements on a warming planet. 2025 IIED, London.

8. Alirol E, Getaz L, Stoll B, Chappuis F, Loutan L. Urbanisation and infectious diseases in a globalised world. *Lancet Infect Dis*. 2011;11: 131–141. doi: 10.1016/S1473-3099(10)70223-1.

9. Alene, ET. Determinant factors for the expansion of informal settlement in Gondar city, Northwest Ethiopia. *J. Urban Manag.* 2022; 11:321-337

10. Neiderud C. How urbanization affects the epidemiology of emerging infectious diseases. *Infect Ecol Epidemiol*. 2015;5: 27060. doi: 10.3402/iee. v5.27060.

11. Fu H, Zhu C. The impact of population influx on infectious diseases - from the mediating effect of polluted air transmission. *Front Public Health.* 2024 Jul 30; 12:1344306. doi: 10.3389/fpubh.2024.1344306. Erratum in: Front Public Health. 2024 Sep 03; 12:1480516. doi: 10.3389/fpubh.2024.1480516. PMID: 39139663; PMCID: PMC11319163.

12. Saba D, Ercan A, Senkaya I, Gebitekin C, Ozkan H. The impact of water, sanitation and hygiene on key health and social outcomes: review of evidence, 2016. UN Children's Fund, New York.

13. Liu Q, Deng J, Yan W, Qin C, Du M, Wang Y, et al. Burden and trends of infectious disease mortality attributed to air pollution, unsafe water, sanitation, and hygiene, and non-optimal temperature globally and in different socio-demographic index regions. *Glob Health Res Policy*. 2024;9: 23. doi: 10.1186/s41256-024-00366-x.

14. Mogaji HO, Olamiju FO, Oyinlola F, Achu I, Adekunle ON, Udofia LE, Edelduok EG, Yaro CA, Oladipupo OO, Kehinde AY, Oyediran F, Aderogba M, Makau-Barasa LK, Ekpo UF. Prevalence, intensity and risk factors of soil-transmitted helminthiasis after five effective rounds of preventive chemotherapy across three implementation units in Ondo State, Nigeria. *PLoS Negl Trop Dis*. 2025 Jan 6;19(1): e0012533. doi: 10.1371/journal.pntd.0012533.

15. Khalil H, Santana R, de Oliveira D, Palma F, Lustosa R, Eyre MT, et al. Poverty, sanitation, and Leptospira transmission pathways in residents from four Brazilian slums. *PLoS Negl Trop Dis.* 2021;15: e0009256. doi: 10.1371/journal.pntd.0009256.

16. Krystosik A, Njoroge G, Odhiambo L, Forsyth JE, Mutuku F, LaBeaud AD. Solid Wastes Provide Breeding Sites, Burrows, and Food for Biological Disease Vectors, and Urban Zoonotic Reservoirs: A Call to Action for Solutions-Based Research. Front Public Health. 2020 Jan 17; 7:405. doi: 10.3389/fpubh.2019.00405.

17. Shafique S, Bhattacharyya DS, Nowrin I, Sultana F, Islam MR, Dutta GK, et al. Effective community-based interventions to prevent and control infectious diseases in urban informal settlements in low- and middle-income countries: a systematic review. *Syst Rev*. 2024;13. doi: 10.1186/s13643-024-02651-9.

18. Zimba B, Mpinganjira S, Msosa T, Bickton FM. The urban-poor vaccination: Challenges and strategies in low-and-middle income countries. *Human Vaccines & Immunotherapeutics*. 2024;20. doi: 10.1080/21645515.2023.2295977.

19. Adams AM, Vuckovic M, Birch E, Brant TA, Bialek S, Yoon D, et al. Eliminating Neglected Tropical Diseases in Urban Areas: A Review of Challenges, Strategies and Research Directions for Successful Mass Drug Administration. *Trop Med Infect Dis.* 2018;3: 122. doi: 10.3390/tropicalmed3040122.

20. Olamiju FO, Nebe O, Mogaji H, Abdus-Salam I, Jenrola L, Marcus AJ, et al. The first mass drug administration campaign for schistosomiasis control in Lagos, Nigeria: lessons for future control programs. *Front in Trop Dis*. 2023;4. doi: 10.3389/fitd.2023.1094320.

21. Gupta M, Thakur JS, Kumar R. Reproductive and child health inequities in Chandigarh Union Territory of India. *J Urban Health*. 2008;85: 291–299. doi: 10.1007/s11524-007-9250-y.

22. Epling SWM, Bjork AM, Martinez Cruz L, Baker MC. Approaches to increase access to community-based infectious disease control for ethnically, racially, and religiously

marginalised populations: a scoping review. *Lancet Infect Dis*. 2025;25: e269–e279. doi: 10.1016/S1473-3099(24)00744-8.

23. The World Bank. World Bank Country and Lending Groups. World Bank Data Help Desk. Available: https://datahelpdesk.worldbank.org/knowledgebase/articles/906519-world-bank-country-and-lending-groups.

24. Peters MDJ, Godfrey C, McInerney P, Khalil H, Larsen P, Marnie C, et al. best practice guidance and reporting items for the development of scoping review protocols. *JBI Evid Synth*. 2022;20: 953–968. doi: 10.11124/JBIES-21-00242.

25. Tricco AC, Lillie E, Zarin W, O'Brien KK, Colquhoun H, Levac D, et al. PRISMA Extension for Scoping Reviews (PRISMA-ScR): Checklist and Explanation. *Ann Intern Med*. 2018;169: 467–473. doi: 10.7326/M18-0850.

26. Pollock D, Peters MDJ, Khalil H, McInerney P, Alexander L, Tricco AC, Evans C, de Moraes ÉB, Godfrey CM, Pieper D, Saran A, Stern C, Munn Z. Recommendations for the extraction, analysis, and presentation of results in scoping reviews. *JBI Evid Synth*. 2023 Mar 1;21(3):520-532. doi: 10.11124/JBIES-22-00123. PMID: 36081365.

27. Turley R, Saith R, Bhan N, Rehfuess E, Carter B. Slum upgrading strategies involving physical environment and infrastructure interventions and their effects on health and socio-economic outcomes. *Cochrane Database Syst Rev*. 2013;(1):CD010067. doi: CD010067. doi: 10.1002/14651858.CD010067.pub2.

28. Henson RM, Ortigoza A, Martinez-Folgar K, Baeza F, Caiaffa W, Vives Vergara A, et al. Evaluating the health effects of place-based slum upgrading physical environment interventions: A systematic review (2012–2018). *Soc Sci Med*. 2020;261: 113102. doi: 10.1016/j.socscimed.2020.113102.

29. Waltz TJ, Powell BJ, Matthieu MM, Damschroder LJ, Chinman MJ, Smith JL, et al. Use of concept mapping to characterize relationships among implementation strategies and assess their feasibility and importance: results from the Expert Recommendations for Implementing Change (ERIC) study. *Implement Sci*. 2015;10: 109. doi: 10.1186/s13012-015-0295-0.

30. Feldstein AC, Glasgow RE. A practical, robust implementation and sustainability model (PRISM) for integrating research findings into practice. *Jt Comm J Qual Patient Saf*. 2008;34: 228–243. doi: 10.1016/s1553-7250(08)34030-6.

31. World Medical Association. World medical association declaration of helsinki: ethical principles for medical research involving human subjects. JAMA. 2013;310(20):2191–4. pmid:24141714

32. Sygeplejerskers samarbejde i N. Ethical guidelines for nursing research in the Nordic countries. Revidert opptrykk ed Typotequet; 1995.

Click here to access/download
Supporting Information
S2 File.docx

Click here to access/download
Supporting Information
S1 File.docx

Click here to access/download
Other
F_PLOS Authorship Change Form.pdf

Exploring how infectious diseases control interventions are delivered in low-income urban communities in urban poor community settings in low- and middle-income countries: a scoping review protocol

Mogaji, H.O.[1,2]Baker, M.C[11], Fatah, I[12], Dorris, C.S[233], and Baker, M.C[14*] Mogaji, H.O.[1,3*]

[1]Department of Global Health, Georgetown University, Washington, United States of America;[21]Department of Behavioral and Applied Sciences, Marian University, Indianapolis, Indiana, United States of America;[32]

[12] Department of Global Health, Georgetown University, Washington, United States of America;

[13] Dahlgren Memorial Library, Department of Global Health, Georgetown University, Washington, United States of America;

[14]Department of Global Health, Department of Global Health, GGeorgetown University, Washington, United States of America;

Dahlgren Memorial Library, Georgetown University, Washington, United States of America;

[3]Department of Behavioral and Applied Sciences, Marian University, Indianapolis, Indiana, United States of America.

Corresponding author*: Email: mcb93@georgetown.edumogajihammed@gmail.com ;

Address: Department of Global Health, Georgetown University, Washington, United States of AmericaDepartment of Behavioral and Applied Sciences, Marian University, Cold Springs Road, 46222, Indianapolis, Indiana, United States of America

Mogaji, H.O: mogajihammed@gmail.com

Baker, M.C: mcb93@georgetown.edu

Fatah, I: iaf4@georgetown.edu

Dorris, C.S: csd24@georgetown.edu

Baker, M.C: mcb93@georgetown.edu

Formatted: Font: 12 pt

Formatted: Line spacing: 1.5 lines

Formatted: Superscript

Formatted: Superscript

Formatted: Superscript

Formatted: Space After: 8 pt, Line spacing: 1.5 lines, Widow/Orphan control

Formatted: Line spacing: 1.5 lines

Formatted: Superscript

Formatted: Superscript

Formatted: Font: (Default) Times New Roman

Formatted: Not Superscript/ Subscript

Formatted: Font: (Default) Times New Roman

Formatted: Font: (Default) Times New Roman

Mogaji, H.O: mogajihammed@gmail.com

Formatted: Font: (Default) Times New Roman

Formatted: Font: (Default) Times New Roman

Abstract

Background:

Infectious diseases thrive in densely populated low-income urban communitiesurban poor areas where living conditions, hygiene, and access to health services are sub-optimal. Information on how to adapt infectious diseases control interventions to be effective in these settings is limited. This scoping review protocol aims to identify specific adaptations of made to infectious disease interventions to delivered in low-income urban communitiesurban poor settings and- the research methods used to test thesereported outcomes to aid understanding and potential future applications. This The proposed scoping review will follow Joanna Briggs Institute (JBI) guidelines and the PRISMA methodology adapted for scoping reviews (PRISMA-ScR). Research questions were created using the Person, Concept, Context (PCC) framework - where the Person person (i.e.,is low-income urban communitiesUrban poor), the Concept concept (i.e.,is interpersonal infectious disease interventions), Context and the context (i.e.,is low- and middle-income countries) framework. Relevant papers published after 1999 2000 will be retrieved using a pre-defined search strategy that incorporatesing all the PCC topics. followed by a comprehensive search of Searches will be made in four databases: MEDLINE, Embase, Global Health (all via Ovid), and Web of Science. Duplicates will be identified and removed with EndNote 20, and all articles will be uploaded into Covidence for screening in two stages using pre-defined inclusion and exclusion criteria. Quantitative and qualitative data will be extracted into Excel spreadsheets and analyzed following the Expert Recommendations for Implementing Change (ERIC) framework.

Ethics Statement:

This study does not require ethical approval because it will extract secondary data from openly accessible published articles.

Conclusion:

Formatted: Highlight

Our review will identify ~~how~~different approaches that have been taken to adapt infectious disease interventions ~~can be adapted and delivered equitably to marginalized populations~~ in low-income urban communities of LMICs~~, and also provide insights that inform future research and practical program~~ , that can be further tested in future programs and studies. ~~design~~We will also highlight methods that have been used to study such interventions in their context. ~~ltimately, this work will contribute to strengthening the evidence base for sustainable, culturally appropriate, and impactful infectious disease interventions in resource-constrained urban settings.The findings from this review will provide understanding on how infectious diseases control interventions were adapted in low-income urban communitiesurban poor settings, and associated implementation and intervention outcomes~~

Keywords:

Infectious diseases, Urban poor, Low Middle-Income Countries, Interpersonal Interventions

Formatted: Font: Not Bold, Font color: Gray-87.5%

Formatted: Font: (Default) Times New Roman, 12 pt

Formatted: Font: Not Bold, Font color: Gray-87.5%

Formatted: Font: (Default) Times New Roman, 12 pt

Formatted: Font: Not Bold, Font color: Gray-87.5%

Formatted: Font: (Default) Times New Roman, 12 pt

Formatted: Font: Not Bold, Font color: Gray-87.5%

Formatted: Font: (Default) Times New Roman

Introduction

Urbanization has accelerated rapidly in this century, with approximately 57% of the world's population now living in cities [1], and . Furthermore, the global urban population is projected to reach 6.25 billion by 2050 [2]. This growth is driven by multiple factors, including government policies on public housing, land use, property ownership and potential for economic opportunities [3]. Cities that contribute to the largest share of national GDPsand are where private sector jobs and consumers are increasingly concentrated [3, 4]. To date, the largest expansion of urban populations has been , and is projected to continue to be, focused on low and middle-income countries (LMICs) [5]. The This rapid pace and scale of urbanization has resulted in significant pressure on available infrastructure and resources, with expansion into areas previously considered uninhabitable, and often referred to as informal settlements or slums [4,6]. For instance, more than 75,000 km^2 of new urban land, equivalent to about 50 times the area of Greater London, has been developed in areas prone to severe flooding for human habitation [4]. These areas, often home to informal settlements, and are characterized by inadequate housing conditions, poor health and safety, and fewer work opportunities [4,6,76]. Over a billion people currently reside in informal settlements in urban areas, with numbers projected to rise by more than 300% by 2030 [7].

The impact of urbanization on the spread of infectious diseases is highly contextualized. On one hand, urbanization can reduce mortality and morbidity caused by infectious agents in areas where living conditions, hygiene, and access to health services are optimal [8]. On the other hand, rapid urbanization has also resulted in the growth of informal settlements [9], where infectious diseases thrive. Factors associated with the spread of infectious diseases in urban areasurban, especially low-income urban communitiesurban poor settings, have been summarized by Alirol et al [8] and Neiderud et al [10]. High population density allows air-borne infections like influenza, measles, and *Mycobacterium tuberculosis* to rapidly spread [11]. Inadequate water and sanitation services contribute to diarrhoeal diseases caused by cholera and *E. coli* [12,13], to the transmission of soil-transmitted helminthiasis and other intestinal parasites [14], and to the proliferation of rodents such as rats which can carry *Leptospira interrogans* [15]. Accumulated waste can provide breeding sites for vectors like sand flies that carry parasites causing leishmaniasisMHOaniasis and of *Aedes* mosquitoes that are vectors for dengue, yellow fever, and chikungunya [16].

While many interventions have proven to be effective in preventing infectious diseases~~ (e.g. WASH, health education, community-based screening, socio-economic support, community-based vector control, behavior change, social mobilization, and electronic(e-) and mobile(m-) health) ~~[17], implementation failure, including in urban settings, has also been ~~widely ~~documented. For example, immunization programs have reported challenges in reaching urban dwellers due to working with large populations that are transient in nature - ~~ (including seasonal workers), ~~with inconsistent employment patterns, and multicultural beliefs and practices~~ ~~[18]. Similarly, ~~-~~mass drug administrations (MDA) for controlling neglected tropical diseases (NTDs) in Africa and the Americas, ~~also report~~have had lower reported coverage in urban settings due to the heterogeneity that exists within urban environments [19,20]. The success of immunization, contraception, and antenatal care services was also reported to be sub-optimal in urban slum areas of Chandigarh in the North of India. [18, 21]. Community health interventions in urban settings, especially, in low-income communities, must therefore contend with population mobility, informal housing, and cultural diversity, which together add complexity to the delivery of services.

These persistent challenges highlight the need to understand how interventions are being adapted in practice. Yet, despite this recognition, there is limited synthesized evidence on adaptations to low income urban communities in LMICs. A recent review begun to address related questions by mapping the types of community-based interventions delivered in informal urban settlements, however they stopped short of providing insight on how interventions were adapted to this setting.[17] These gaps demonstrate the need for a review that specifically identifies approaches taken to adapting interventions and how these were studied.

This review will address that gap by concentrating on a subset of population-based mass interventions characterized by including one-on-one interactions between a distributor and a recipient within the community. This delivery mode, often referred to as ‘campaigns’, is commonly used for vaccinations, mass drug administration (MDA), screening and test-and-treat initiatives, and distribution of products such as bed nets, condoms, and facemasks. This focus

Formatted: Font color: Auto
Formatted: Font color: Auto
Formatted: Font color: Auto
Formatted: Font color: Auto
Formatted: Font color: Auto
Formatted: Font color: Auto
Formatted: Font color: Auto
Formatted: Font color: Auto
Formatted: Font color: Auto
Formatted: Font color: Auto
Formatted: Font color: Custom Color(RGB(34,34,34))
Formatted: Font: (Default) Times New Roman

aligns with the methodological approach taken in a previous scoping review focused on a different marginalized population, namely ethnically, racially, and religiously marginalized populations. [22]

Formatted: Font: (Default) Times New Roman

Aim and objectives of the review

Formatted: Justified, Space Before: 12 pt, After: 12 pt, Line spacing: 1.5 lines

Formatted: Font: (Default) Times New Roman

The aim of this scoping review is to map and synthesize evidence on how infectious disease interventions have been adapted for delivery in low-income urban communities in LMICs. In line with this aim, our objectives are to: (1) catalogue the types of adaptations reported; (2) examine how these adaptations relate to context; (3) assess the extent and distribution of the available evidence; and (4) review the study designs and methods used to investigate these adaptations.

Materials and Methods

~~Protocol Design~~

~~Scoping reviews use standardized methods, taking a systematic approach, and are the methodology of choice when the aim is to get a broad overview of a potentially diverse body of literature [24]. Hence, we will conduct a scoping review using the Joanna Briggs Institute (JBI) guidelines [24] and utilize the PRISMA extension for scoping reviews (PRISMA-ScR) guidelines for reporting [25]. This protocol has been written following the Preferred Reporting Items for Systematic Review and Meta-Analysis Protocols (PRISMA-P) [See S1 File]~~

Protocol design

Scoping reviews use standardized methods, taking a systematic approach, and are the methodology of choice when the aim is to get a broad overview of a potentially diverse body of literature [24] and is therefore the method of choice for this study. ~~Hence, this~~This protocol documents how we will conduct a scoping review using the Joanna Briggs Institute (JBI) guidelines [24], -and the Preferred Reporting Items for Systematic Review and Meta-Analysis extension for Scoping Reviews (PRISMA-ScR) guidelines [25]. ~~This protocol has been written using the Preferred Reporting Items for Systematic Review and Meta-Analysis Protocols (PRISMA-P) guidelines [See S1 File], and~~It has -also been registered on the Open Science Framework (OSF), accessible here https://doi.org/10.17605/OSF.IO/T8EWA.

~~Methodological Framework~~

~~This scoping review will use~~In this review we used the Person, Concept, Context (PCC) framework to guide the formation of research questions and search strategies [26]. Our population will include those living in low-income urban communities.~~urban poor communities.~~ The concept will be adaptations of infectious disease interventions characterized by one-on-one interactions

Formatted: Line spacing: 1.5 lines
Formatted: Justified, Line spacing: 1.5 lines
Formatted: Line spacing: 1.5 lines
Formatted: Space Before: 0 pt, After: 8 pt, Line spacing: 1.5 lines
Formatted: Font: Bold
Formatted: Line spacing: 1.5 lines
Formatted: Font: Not Bold
Formatted: Font: (Default) Times New Roman

between a distributor and a recipient within the community. The context will be low-middle-income countries as defined by the World Bank.

Eligibility Criteria

Our inclusion and exclusion criteria is detailed in Table 1. ~~We will include studies of infectious disease interventions delivered in urban poor settings in low- and middle-income countries (LMICs). Eligible interventions are those delivered at a population level but involving interpersonal interaction (e.g., vaccination, mass drug administration, community-based screening, distribution of medicines or commodities). We will include peer-reviewed articles published from 2000 onwards, in any language, provided an English abstract is available (Table 1).~~ We consider studies published from 2000 relevant because many large-scale infectious disease control initiatives in LMICs— - including global immunization campaigns, scale-up of HIV/TB programs, and mass drug administration for NTDs— - expanded significantly after 2000, often with increased attention to delivery in urban contexts. We exclude studies based in rural areas, not because these studies are less important, but to align with our stated objective.

~~We will exclude interventions delivered exclusively in rural settings or in high-income country contexts. We will also exclude studies that focus only on individual clinical care without a community or population-level delivery component, as well as studies describing only mass communication interventions (e.g., radio campaigns) that lack interpersonal delivery. Editorials, commentaries, and opinion pieces without primary data will also be excluded (Table 1).~~

~~Information sources and search strategy~~

~~Relevant articles published from the year 2000~~ onwards ~~until [the date of search] will be retrieved from MEDLINE, Embase, Global Health (all via Ovid), and Web of Science with the goal to~~

Formatted: All caps

Formatted: Font: Not Bold, Highlight

Formatted: Font: Not Bold, Font color: Red

Formatted: Font: Not Bold, Font color: Red

Formatted: Font: Not Bold, Font color: Red

Formatted: Font: Not Bold, Font color: Red

Formatted: Font: Not Bold, Highlight

Formatted: Font: Not Bold, Highlight

Formatted: Font: Not Bold

identify articles from both medical and social sciences fields. The search strategy will incorporate both database-specific subject headings and keywords that represent all three PCC topics: low-income urban communitiesurban poor, infectious disease interventions, and LMICs. As there is no universally accepted global definition of urban, we will use search terms like "urban," "peri-urban," "city," "municipality," "urbanization," and "township." used in other literature reviews on this subject. [17, 17,28]. Terms related to low-income urban communitiesurban poor populations including slums, informal settlements, street dwellers, will also be used to select for the population of interest. As our concept focuses on adapting the type of infectious disease interventions that require interpersonal interaction between those delivering a health product and those receiving it, in a community setting, we will develop search terms related to vaccine, mass drug administration, and mass screening or testing for infectious diseases. We will develop the search strategy by running initial search terms in Ovid MEDLINE and test the results to obtain an optimal balance between allowing for a broad scope and feasibility. We will then translate the search terms for the remaining databases. [see S2 File]

Inclusion and Exclusion Criteria

We will include peer-reviewed studies published from the year 2000 onwards that focus on individuals residing in low-income urban communitiesurban poor areas of low- and middle-income countries (LMICs). When finalizing the eligibility criteria and search strategy, we referred to The World Bank Classifications to determine which LMICs should be included.[citation] Eligible studies must involve infectious diseases control interventions requiring interpersonal contact in a community setting, such as immunization, mass drug administration, screening, contact tracing, or distribution of health commodities. Studies must report on efforts made to adapt interventions to increase access to our defined population (providing at least a minimum description of the intervention), include some form of evaluation of the intervention and report on at least one outcome. Only community-based studies involving human subjects will be included. Studies other than literature reviews, editorials, opinion pieces, grey literature will be included. All languages will be included as the authors spoke several languages among them and other languages could be rapidly translated using online software. Articles will be excluded if they do not meet the inclusion criteria. All inclusion and exclusion criteria are summarized in Table 1.

Formatted: Indent: First line: 0", Space Before: 0 pt, After: 8 pt, Line spacing: 1.5 lines

Table 1: Inclusion and exclusion criteria

**Framework**	**Items**	**Inclusion**	**Exclusion**
Population	Individuals	Studies concerning individuals residing in low-income urban communities~~urban poor areas~~	Studies that are not specific to individuals residing in low-income urban communities~~urban poor areas~~
Concept~~text~~	Type of intervention	Studies on Interventions that require interpersonal contact in a community setting, specifically: immunizations, mass drug administration/ deworming, screening, contact tracing, distribution of commodities (e.g. condoms, facemasks, bed nets).	Studies on Interventions that do not rely on one-one interaction - either reaching many people simultaneously (e.g. mass communication health education campaigns targeting behavior change) or interventions that rely on environmental modifications (e.g. spraying for vector control or water and sanitation projects).<br>~~In contrast, we will exclude interventions that do not rely on interpersonal interaction, such as environmental modifications (e.g. sewage and trash collection as a potential strategy to reduce rodents or spraying for vector control) and mass communication or health education for behavior change.~~

Formatted: Line spacing: 1.5 lines

Formatted Table

Formatted: Not Highlight

Concep t	Information on adaptation	Studies that describe how the intervention was adapted to low-income urban communitiesurban poor setting, even if very limited.	Studies that did not describe how the intervention was adapted to low-income urban communitiesurban poor setting
	Information on Evaluation	Evaluation studies about the intervention in low-income urban communitiesurban poor setting (this can be tested as part of or before the research study)	Formative studies designed to collect information to inform the design of adapted interventions, prior to any adaptations having been made.
Concep t	Reported outcome	Studies must report at least one outcome (e.g., implementation, clinical/epidemiologic, economic, or experiential) indicating that the intervention was delivered, and evaluated in practice.Studies that report at least one implementation or intervention outcome (e.g. coverage, cost, prevalence, knowledge/ attitudes, etc	Studies that did not report any implementation or intervention outcome e.g. coverage, cost, prevalence, knowledge/ attitudes, etc
Context	Countries	Studies implemented in -Low- and Middle-Income Countries (LMICs)	Studies implemented in High Income Countries (HICs)
Context	Study settings Residence	Studies where authors implemented at the community level studies were included if authors described the setting as a low-income urban community using any of our search terms including: "slum," "informal settlement," "street dwellers.	Studies that are hospital-based, or conducted in cities, rural communities, or high-income urban communities - even within LMIC.

Formatted: Line spacing: single

Formatted: Line spacing: single

Formatted: Font: (Default) Times New Roman, Not Highlight

Formatted: Line spacing: single

Formatted: Font: (Default) Times New Roman, Not Highlight

Formatted Table

Context	Study design and type of publication ~~Type of study~~	Studies that are peer-~~Peer-~~reviewed and published.~~Research studies~~ ~~.(e.g. r~~ Can be qualitative, quantitative or mixed methods, and also descriptive or analytic. ~~retrospective case studies, quasi-experimental e.g. before and after studies; RCTs). Can be qualitative or quantitative or mixed methods.~~	Literature reviews/textbooks/ dissertations/ editorials/ opinion pieces/ grey literature
~~Context~~	~~Study subject~~	~~Studies about Human species~~	~~Studies about non-Human species~~
~~Context~~	~~Year of publication~~	~~Studies published from 2000 till date~~	~~Studies published prior 2000~~
Context	Publication l~~L~~anguage	Studies reported in all languages	

Information sources and search strategy

Relevant articles published from the year 2000 onwards until ~~[~~the date of search ~~]~~ will be retrieved from MEDLINE, Embase, Global Health (all via Ovid), and Web of Science with the goal to identify articles from both medical and social sciences fields. The search strategy ~~will~~ incorporates both database-specific subject headings and keywords that represent all three PCC topics: low-income urban communities, infectious disease interventions, and LMICs. As there is no universally accepted global definition of urban, we will use search terms like "urban," "peri-urban," "city," "municipality," "urbanization," and "township." used in other literature reviews on this subject. [17,28]. Terms related to low-income urban communities including slums, informal settlements, street dwellers, ~~will~~are also ~~be~~ used to select for the population of interest. As our concept focuses on mass ~~adapting the type of~~ infectious disease interventions that require interpersonal interaction between those delivering a health product and those receiving it ~~, in a community setting,~~ we ~~will develop~~included search terms related to vaccine, mass drug administration, and mass screening or testing for infectious diseases. The ~~preliminary~~ search strategy for Ovid MEDLINE is

Formatted: Line spacing: 1.5 lines

Formatted: Font: Bold

Formatted: Font color: Custom Color(RGB(27,27,27))

Formatted: Font: Font color: Gray-87.5%

~~illustrated~~shown in [see S1~~2~~ File]. The search strategy ~~will be~~was developed ~~peer-reviewed~~ by a university librarian specialist and ~~the~~peer-reviewed with the authors, ~~and further~~then tested to obtain an optimal balance between allowing for a broad scope and feasibility. ~~We will then translate the~~These search terms were translated for the remaining databases. ~~[see S2 File]~~

Study selection~~**Article screening process**~~

All retrieved articles will be uploaded into Covidence Software for screening. ~~after duplicates have been removed.~~ Duplicates will be identified and removed with EndNote 20 (Clarivate Analytics, Philadelphia, PA). Article screening will be performed by four trained research assistants. In the first phase, titles and abstracts of at least 200 articles will be independently reviewed by two researchers until an inter-rater reliability of 95% agreement is achieved. Discrepancies will be resolved through discussion with peers and, if needed, a senior researcher. Once this threshold is reached, screening will proceed with one researcher per article. ~~Prior to study selection, we will perform two To ensure clarity of the inclusion and exclusion criteria we will perform two phased pilot screening involvings of 200 article in the first phase, and 50 articles in the second phase. Articles randomly selected articles with BMC, FI, and HOM. These articles will each be screened independently by two reviewers and discrepancies discussed. We will proceed with full title and abstract review once conflict is below 5% of every 100 articles screened. In the first stage of full screening, we will first review 100 of titles and abstracts against inclusion and exclusion criteria. To ensure calibration between the reviewers and to increase quality, all articles will be double reviewed at this stage. Conflicts that arise at this stage of screening will be resolved initially through discussions between screeners, with remaining conflicts discussed with BMC and MHO. Sources~~Articles included in the title and abstract stage of screening will then undergo full-text review, with the same eligibility criteria used to determine relevance. A PRISMA flow diagram will be created to summarize the results of the search and reasons for exclusion. ~~Subsequently, reviewers~~Reviewers will continue to meet every 1-2 weeks to discuss the studies, including any articles for which there is uncertainty on whether to include it.

Formatted: Font: Font color: Gray-87.5%

Formatted: Font: Font color: Gray-87.5%

Formatted: Font: Font color: Gray-87.5%

Formatted: Font: Font color: Gray-87.5%

Formatted: Font: Font color: Gray-87.5%

Formatted: Font: Font color: Gray-87.5%

Formatted: Font: Bold

Data Extraction

The data extraction process will be conducted by fourtwo researchers who. Two researchers will independently extract data from assigned articles, while the other two will perform blinded validation of accuracy.. Any disagreements will be resolved through discussion until consensus is reached. A standardized data extraction form in Microsoft Excel- will be used to capture both quantitative and qualitative information. InitialThe extraction form will include information on the topics presented in table 2 as well as bibliographic and study characteristics such as author, list, year of publication, and language. e, study design, objectives, research questions, study type, study participants, and other relevant contextual details. Subsequently, information on the intervention, its context, adaptations, outcomes, and measures will be collected using the framework outlined in Box 1. The extraction framework was developed with reference to the Expert Recommendations for Implementing Change (ERIC) framework [29] and the Practical, Robust Implementation and Sustainability Model (PRISM) [30], both of which are widely applied in implementation research. All extracted data will first be reviewed independently and then cross-checked by BMC and MHO to ensure consistency and accuracyby a senior researcher.

The data extraction process will be carried out by four researchers. The first two researchers will randomly extract data from assigned articles, and the other two researchers will extract data blinded from each of the first set of researchers. Any disagreement will be resolved by discussion. Quantitative and qualitative data will be extracted by researchers using a data extraction form in Microsoft excel. Details such as , including data on the authors list, year of publication, language of publication, study design, study objectives, research questions, study type, study participants, and other context relevant data will be first extracted. Following this, information on intervention, context, adaptation, outcomes and measures will be extracted using Box 1. The extraction framework type, intervention goal and objectives will be extracted. year and language of the publication, the study design (including research question, type of data collected, and outcomes measured) and on the implementation of the intervention (including the context, adaptations made, and outcomes). The extraction framework related to the intervention (see Box 1) was developed

Formatted: Font: Not Bold
Formatted: Font: Not Bold
Formatted: Font: Not Bold
Formatted: Font: Not Bold
Formatted: Font: Not Bold
Formatted: Font: Not Bold
Formatted: Font: Not Bold
Formatted: Font: Not Bold
Formatted: Font: Not Bold
Formatted: Font: Not Bold
Formatted: Font: Not Bold

with reference to concepts identified in Expert Recommendations for Implementing Change (ERIC) framework [29] and the Practical, Robust Implementation and Sustainability Model (PRISM) [30]. The former framework has been used in a similar literature review focused on different population groups [22], and the latter is used widely in implementation research [30]. The data will initially be abstracted independently and then reviewed by BMC and MHO.

Box 1: Information to be extracted about the interventions and context and their reported outcomesIntervention specific elements we will include in extraction framework

Intervention:

- What the intervention is e.g. bednet distribution, health education campaign, screening
- What the intervention type is e.g., preventive, diagnostic, curative
- Why the intervention was needed
- Intervention goal, including disease(s) targetedtargetted

Context:

- Where implemented: Countries, sub-country, number of sites
- Community setting environment: socio- economic, geography, political
- Population receiving intervention: who, perceptions on the intervention and on organizations delivering the intervention

Implementation and adaptation made to the context:

- How did decisions made to do things a specific way relate to the context?
- Delivery modalities with explanation on why selected: Point of delivery, Timing.
- How the community was initially approached and how they were engaged during each stage of the study: design, implementation, and review
- What organization(s) led delivery of the intervention, who within these organizations, and what were their perceptions on intervention and the community?
- Who were other stakeholders, what was their role, and why were they engaged?
- What processes were put in place to coordinate between all stakeholders?
- Adoption / uptake of intervention e.g. number of workshops, amount of product delivered, etc
- How did decisions made to do things a specific way relate to the context?
- What worked well and why (include facilitators)
- What did not work and why (include barriers and if/how overcome)
- Sustainability: how long were the interventions delivered for and what mention was there of maintaining activities/ outcomes long term.

Formatted: Font: Font color: Red

Formatted: Font: Font color: Red

Formatted: Space After: 0 pt

Formatted: Indent: Left: 0.5", Space After: 0 pt, No bullets or numbering

Formatted: Space After: 0 pt

Formatted: Line spacing: single

Formatted: Indent: Left: 0.5", Space After: 0 pt, No bullets or numbering

Formatted: Space After: 0 pt

Formatted: Space After: 0 pt

- Costs and funding

~~Outcomes and measures:~~

~~- Effectiveness: e.g. reduced costs, reduced disease, increased knowledge, coverage~~

~~- Sustainability: how long werewas the interventions delivered for and what mention was there of maintaining activities/ outcomes long term.~~

Analysis of results

~~Extracted data will be analyzed by counting frequency and qualitatively by reading through the summary data extracted, writing synthesized narratives on each element, and looking for patterns such as common themes and context specific differences. The research team will meet regularly during the analysis phase to share draft findings and ask probing questions that will prompt deeper analysis.~~

We will analyze data using both descriptive summaries and thematic narrative synthesis, structured in a way that enables identification of research gaps. For descriptive summaries we will present tables summarizing basic study characteristics: country (and region), year of publication, intervention type, type of urban setting (e.g., informal settlement vs other), and population demographics. ~~The the thematic narrative synthesis, we will extract textual data to identify themes regarding how interventions were adapted: what was reported by the authors to work, what didn't, barriers and facilitators, how stakeholders were engaged, context-driven decisions, and contextual variables (socio-economic, geographic, political) that shaped adaptation.~~ All elements of ~~The narrative synthesis will capture information on the reach, adoption, effectiveness, implementation success or barriers and how sustainable the interventions were.~~ m~~M~~ixed or multi-component studies will be extracted ~~as separate entries, with the author list replicated.~~ . ~~They will first be analyzed individually and then as combined interventions, enabling discussion of the adaptation strategies applied and their influence on other intervention outcomes or measures.~~ We will not attempt meta-analysis or effectiveness estimates as this review focuses on ~~what~~identifying how interventions ~~has been done and reported~~have been adapted and how these have been studied.

Formatted: Space After: 0 pt

Formatted: No bullets or numbering

Formatted: Space After: 0 pt, No bullets or numbering

Formatted: Line spacing: 1.5 lines

Formatted: Font: Font color: Red

Formatted: Font: Font color: Red

Formatted: Font: Font color: Red

Formatted: Font: Font color: Red

Formatted: Font: Font color: Red

Formatted: Font: Font color: Red

Formatted: Font: Font color: Red

Thematic analysis will focus on how interventions were adapted; what was reported to work and not work; barriers and facilitators; how stakeholders were engaged; context-driven decisions; and the socio-economic, geographic, and political variables that shaped adaptation. Analysis will be qualitative and conducted by the two senior researchers. All textual data will first be carefully reviewed and emerging themes identified. Additional columns will then be created in the extraction spreadsheet, and thematic labels (codes) manually entered. Results will be synthesized within each theme, with a detailed description of common trends as well as notable exceptions. To complement researcher perspectives, ChatGPT will be queried to identify potential additional themes and to further explore the data within each theme. Any information provided by ChatGPT will be manually cross-checked against the original data sources before inclusion.~~Qualitative synthesis will be managed manually in combination with other tools such as NVivo software and AI.~~

~~**Expected outcome of the study**~~

~~This study aims to improve understanding of how infectious disease interventions are adapted for low-income urban communitiesurban poor settings. It is expected to identify effective adaptation strategies, key implementation and intervention outcomes, and the facilitators and barriers influencing success. The findings will also generate practical recommendations to inform future research and guide program design and delivery in similar contexts.~~

Status and timeline of the study

The scoping review will be conducted over a six-month period, from August 2025 to January 2026. Development of the search strategy will take place in August, followed by the initial screening of titles and abstracts within the same month. Full-text screening will occur in September, followed by data extraction in October. Data analysis and interpretation will take place in November.

~~**Dissemination plan**~~

Formatted: Font: Font color: Red

Formatted: Font: Font color: Red

Formatted: Normal (Web), Left, Space Before: 0 pt, After: 10 pt, Line spacing: 1.5 lines

Formatted: Normal (Web), Left, Space Before: 0 pt, After: 0 pt, Line spacing: single

Formatted: Line spacing: 1.5 lines

Formatted: Font: Bold

The results from the scoping review will be submitted for publication in an open-access peer-reviewed journal, and will be shared through institutional websites and other web pages. The written as a manuscript in FebruaryDecember, and abstracts from this review will also be prepared for submission to conferences. Fully revised manuscript will be submitted to an open-access journal for publication consideration.

Protocol deviation

Formatted: Font: Bold

Any deviations from the described protocol will be documented and transparently reported in future publications and presentations of study results. The research team will ensure that any deviations are justified, minimized, and do not compromise the integrity or objectives of the study

Formatted: Font: Font color: Gray-87.5%, Highlight

Data-sharing

Formatted: Font: Bold

In line with open science principles, we will make the final data extraction sheet and study metadata openly available through a public repository such as the Open Science Framework upon completion of the review.

Formatted: Font: Font color: Gray-87.5%, Highlight

Ethical considerations

Formatted: Font: Bold

Ethical approval was not required because the study does not include participants. However, this study is in accordance with the Declaration of Helsinki [31] and Ethical Guidelines for Nursing Research in the Nordic Countries [32].

Formatted: Font: Font color: Gray-87.5%, Highlight

Patient and public involvement

Formatted: Font: Bold

None

Formatted: Font: (Default) Times New Roman

Formatted: Font: Bold

Discussion

Our overarching aim in this review is to map and synthesize evidence on how infectious disease interventions have been adapted for delivery in low-income urban communities in LMICs. ~~to synthesize existing literature to strengthen the evidence base on delivering infectious disease interventions equitably to marginalized populations in low-income urban communities in LMICs.~~ The findings will enhance understanding of how such interventions ~~are~~might be adapted for these communities and how adaptations relate to context~~, with a focus on identifying strategies that are effective, culturally and context-specific, low-cost, and sustainable~~. ~~Additionally, the~~The review will highlight key facilitators and barriers to implementing ~~successful~~ adaptations as well as methods used to study these ~~–~~, providing valuable insights to guide future research and practice in similar contexts.

The strengths of this review include its methodological rigor, with procedures designed to minimize selection bias. These are reinforced by the authors' prior experience conducting a related review among other marginalized groups [22], collaboration with a senior trained librarian, and the inclusion of articles published in multiple languages, supported by the team's diverse linguistic capabilities. We also employed a pilot-screening approach to strengthen selection procedures, drew upon the team's extensive experience delivering infectious disease interventions in LMICs across three world regions, and applied established frameworks for data extraction and analysis. ~~The strengths of our review include its methodological rigor, designed to minimize selection bias. These are supported by the authors' prior experience conducting a similar review in high-income countries among marginalized groups [22], as well as the inclusion of articles in all languages, reflecting the team's diverse linguistic capabilities. We also integrate a pilot-screening approach into our selection procedures, draw upon the team's extensive years of experience delivering infectious disease interventions in LMICs, and adopt established frameworks for data extraction and analysis. Furthermore, our synthesis will benefit from applying the RE-AIM framework to ensure a structured and comprehensive interpretation of findings.~~

Formatted: Font: Font color: Blue

Formatted: Font: Font color: Blue

Formatted: Font: Font color: Blue

Formatted: Font: Font color: Blue

Formatted: Font: Font color: Blue

Formatted: Font: Font color: Blue

Formatted: Font: Font color: Blue

Formatted: Font: Font color: Blue

Formatted: Font: Font color: Blue

Formatted: Line spacing: 1.5 lines

A potential limitation of our review is the availability of studies that meet our inclusion criteria. The delivery of infectious disease interventions is usually led by health program implementers and frontline workers, with relatively few academic researchers engaged in sustained collaboration with these groups. More broadly, implementation research - and the networks linking academics with program practitioners - remains a relatively nascent field. As a result, the pool of eligible studies may be limited; however, documenting this gap will itself be an important contribution. A potential limitation of our review is the availability of studies that meet our strict inclusion criteria. The delivery of infectious disease interventions is often the remit of health program implementers and frontline workers, with relatively few academic researchers maintaining strong collaborations with these groups. More broadly, implementation research and the networks bridging academics with program practitioners remain relatively nascent fields. As such, the pool of eligible studies may be limited

Dissemination plan

The results from the scoping review will be submitted for publication in a n open access peer-reviewed journal, and will be shared through institutional websites and other web pages. The written as a manuscript in FebruaryDecember, and abstracts from this review will also be prepared for submission to conferences.

Conclusion

Our review will provide critical insights into howapproaches used to adapt infectious disease interventions are adapted and delivered equitably tofor marginalized populations in LMIC low-income urban communities of LMICs. By identifying effective strategies and highlighting contextual facilitators and barriers, the study will inform both future research and practicalinnovative program design. Ultimately, this work will contribute to strengthening the evidence base for sustainable, culturally appropriate, and impactful infectious disease interventions in resource-constrained urban settings.

Formatted: Font: Font color: Blue

Formatted: Font: Font color: Blue

Formatted: Line spacing: 1.5 lines

Formatted: Font: Bold

Formatted: Font: Bold, Font color: Gray-87.5%

Formatted: Font: Font color: Gray-87.5%

Formatted: Font: Font color: Gray-87.5%

Formatted: Font: Font color: Gray-87.5%

Formatted: Font: Font color: Gray-87.5%

Formatted: Font: Font color: Gray-87.5%

Supplementary Information

~~S1 File: Completed PRISMA-P 2015 checklist~~

~~S2~~ S1 File: Search terminology.~~PRISMA numbers from Search Strategy~~

Declarations

Authors' contributions

~~BMC~~ MBC conceptualized the study; ~~BMC~~MBC, MHO, ~~FI~~ IF prepared the protocol; BMC, MHO, DCS designed the search strategy; DCS constructed the search framework; MHO prepared the first version for the manuscript. All authors revised and approved the manuscript. BMC is the guarantor of the review

Ethics approval and consent to participate

Ethics approval will not be required. Patients nor the public will not be involved at any stage of this scoping review.

Consent for publication

Not applicable

Availability of data and materials

Formatted: Line spacing: 1.5 lines, Pattern: Clear (White)

Formatted: Justified, Space Before: 12 pt, After: 12 pt, Line spacing: 1.5 lines

Formatted: Line spacing: 1.5 lines

~~All data are in the manuscript and/or supporting information files. However, upon~~ No data has been collected yet. Upon completion of the review~~, data will be extracted from published articles that are already accessible online, and~~ the final extraction sheet will be provided as a supplementary file.

Funding

No funding was received for this study.

Formatted: Font: (Default) Times New Roman

Formatted: Font: (Default) Times New Roman

Formatted: Font: (Default) Times New Roman

References

1. World Bank Group. World Urbanization Prospects, United Nations. https://data.worldbank.org/indicator/SP.URB.TOTL.IN.ZS

2. United Nations Department of Economic and Social Affairs. 2014 revision of the World Urbanization Prospects. https://www.un.org/en/development/desa/publications/2014-revision-world-urbanization-prospects.html

3. Yu D, Li X, Yu J, Shi X, Liu P, Tian P. Whether Urbanization Has Intensified the Spread of Infectious Diseases—Renewed Question by the COVID-19 Pandemic. *Front Public Health*. 2021;9. doi: 10.3389/fpubh.2021.699710.

4. World Bank Group. Urban Development. https://www.worldbank.org/en/topic/urbandevelopment/overview

5. Gunaratna, KL, Eduardo Cesar LM, Yansui L, Liana R, Akiko O, Obudho R, Berma M, Sumanm KK, Jan Q, Harrison P, Maharaj B, Knowles C, Sandro G, Seto KC, Ndilila FM. IAP Statement on Implications of Urbanization in Low- and- Middle- Income Countries. The Inter Academy Partnership, 2022.

6. United Nations. Rapid urbanization and population growth are outpacing the construction of adequate and affordable housing. https://unstats.un.org/sdgs/report/2019/goal-11/

7. Cociña, C and Landesman, T. Better cities are possible: transforming informal settlements on a warming planet. 2025 IIED, London.

8. Alirol E, Getaz L, Stoll B, Chappuis F, Loutan L. Urbanisation and infectious diseases in a globalised world. *Lancet Infect Dis*. 2011;11: 131–141. doi: 10.1016/S1473-3099(10)70223-1.

Formatted: Font: (Default) Times New Roman

Formatted: Font: (Default) Times New Roman

Formatted: Font: (Default) Times New Roman

Formatted: Font: (Default) Times New Roman

Formatted: Font: (Default) Times New Roman

9. Alene, ET. Determinant factors for the expansion of informal settlement in Gondar city, Northwest Ethiopia. *J. Urban Manag.* 2022; 11:321-337

10. Neiderud C. How urbanization affects the epidemiology of emerging infectious diseases. *Infect Ecol Epidemiol*. 2015;5: 27060. doi: 10.3402/iee.v5.27060.

11. Fu H, Zhu C. The impact of population influx on infectious diseases - from the mediating effect of polluted air transmission. *Front Public Health.* 2024 Jul 30;12:1344306. doi: 10.3389/fpubh.2024.1344306. Erratum in: Front Public Health. 2024 Sep 03;12:1480516. doi: 10.3389/fpubh.2024.1480516. PMID: 39139663; PMCID: PMC11319163.

12. Saba D, Ercan A, Senkaya I, Gebitekin C, Ozkan H. The impact of water, sanitation and hygiene on key health and social outcomes: review of evidence, 2016. UN Children's Fund, New York.

13. Liu Q, Deng J, Yan W, Qin C, Du M, Wang Y, et al. Burden and trends of infectious disease mortality attributed to air pollution, unsafe water, sanitation, and hygiene, and non-optimal temperature globally and in different socio-demographic index regions. *Glob Health Res Policy*. 2024;9: 23. doi: 10.1186/s41256-024-00366-x.

14. Mogaji HO, Olamiju FO, Oyinlola F, Achu I, Adekunle ON, Udofia LE, Edelduok EG, Yaro CA, Oladipupo OO, Kehinde AY, Oyediran F, Aderogba M, Makau-Barasa LK, Ekpo UF. Prevalence, intensity and risk factors of soil-transmitted helminthiasis after five effective rounds of preventive chemotherapy across three implementation units in Ondo State, Nigeria. *PLoS Negl Trop Dis*. 2025 Jan 6;19(1):e0012533. doi: 10.1371/journal.pntd.0012533.

15. Khalil H, Santana R, de Oliveira D, Palma F, Lustosa R, Eyre MT, et al. Poverty, sanitation, and Leptospira transmission pathways in residents from four Brazilian slums. *PLoS Negl Trop Dis.* 2021;15: e0009256. doi: 10.1371/journal.pntd.0009256.

16. Krystosik A, Njoroge G, Odhiambo L, Forsyth JE, Mutuku F, LaBeaud AD. Solid Wastes Provide Breeding Sites, Burrows, and Food for Biological Disease Vectors, and Urban Zoonotic Reservoirs: A Call to Action for Solutions-Based Research. Front Public Health. 2020 Jan 17;7:405. doi: 10.3389/fpubh.2019.00405.

17. Shafique S, Bhattacharyya DS, Nowrin I, Sultana F, Islam MR, Dutta GK, et al. Effective community-based interventions to prevent and control infectious diseases in urban informal settlements in low- and middle-income countries: a systematic review. *Syst Rev*. 2024;13. doi: 10.1186/s13643-024-02651-9.

18. Zimba B, Mpinganjira S, Msosa T, Bickton FM. The urban-poor vaccination: Challenges and strategies in low-and-middle income countries. *Human Vaccines & Immunotherapeutics*. 2024;20. doi: 10.1080/21645515.2023.2295977.

19. Adams AM, Vuckovic M, Birch E, Brant TA, Bialek S, Yoon D, et al. Eliminating Neglected Tropical Diseases in Urban Areas: A Review of Challenges, Strategies and Research Directions for Successful Mass Drug Administration. *Trop Med Infect Dis.* 2018;3: 122. doi: 10.3390/tropicalmed3040122.

20. Olamiju FO, Nebe O, Mogaji H, Abdus-Salam I, Jenrola L, Marcus AJ, et al. The first mass drug administration campaign for schistosomiasis control in Lagos, Nigeria: lessons for future control programs. *Front in Trop Dis*. 2023;4. doi: 10.3389/fitd.2023.1094320.

21. Gupta M, Thakur JS, Kumar R. Reproductive and child health inequities in Chandigarh Union Territory of India. *J Urban Health*. 2008;85: 291–299. doi: 10.1007/s11524-007-9250-y.

22. Epling SWM, Bjork AM, Martinez Cruz L, Baker MC. Approaches to increase access to community-based infectious disease control for ethnically, racially, and religiously

marginalised populations: a scoping review. *Lancet Infect Dis*. 2025;25: e269–e279. doi: 10.1016/S1473-3099(24)00744-8.

23. The World Bank. World Bank Country and Lending Groups. World Bank Data Help Desk. Available: https://datahelpdesk.worldbank.org/knowledgebase/articles/906519-world-bank-country-and-lending-groups.

24. Peters MDJ, Godfrey C, McInerney P, Khalil H, Larsen P, Marnie C, et al. Best practice guidance and reporting items for the development of scoping review protocols. *JBI Evid Synth*. 2022;20: 953–968. doi: 10.11124/JBIES-21-00242.

25. Tricco AC, Lillie E, Zarin W, O'Brien KK, Colquhoun H, Levac D, et al. PRISMA Extension for Scoping Reviews (PRISMA-ScR): Checklist and Explanation. *Ann Intern Med.* 2018;169: 467–473. doi: 10.7326/M18-0850.

26. Pollock D, Peters MDJ, Khalil H, McInerney P, Alexander L, Tricco AC, Evans C, de Moraes ÉB, Godfrey CM, Pieper D, Saran A, Stern C, Munn Z. Recommendations for the extraction, analysis, and presentation of results in scoping reviews. *JBI Evid Synth*. 2023 Mar 1;21(3):520-532. doi: 10.11124/JBIES-22-00123. PMID: 36081365.

27. Turley R, Saith R, Bhan N, Rehfuess E, Carter B. Slum upgrading strategies involving physical environment and infrastructure interventions and their effects on health and socio-economic outcomes. *Cochrane Database Syst Rev*. 2013;(1):CD010067. doi: CD010067. doi: 10.1002/14651858.CD010067.pub2.

28. Henson RM, Ortigoza A, Martinez-Folgar K, Baeza F, Caiaffa W, Vives Vergara A, et al. Evaluating the health effects of place-based slum upgrading physical environment interventions: A systematic review (2012–2018). *Soc Sci Med*. 2020;261: 113102. doi: 10.1016/j.socscimed.2020.113102.

Formatted: Font: (Default) Times New Roman

29. Waltz TJ, Powell BJ, Matthieu MM, Damschroder LJ, Chinman MJ, Smith JL, et al. Use of concept mapping to characterize relationships among implementation strategies and assess their feasibility and importance: results from the Expert Recommendations for Implementing Change (ERIC) study. *Implement Sci*. 2015;10: 109. doi: 10.1186/s13012-015-0295-0.

30. Feldstein AC, Glasgow RE. A practical, robust implementation and sustainability model (PRISM) for integrating research findings into practice. *Jt Comm J Qual Patient Saf*. 2008;34: 228–243. doi: 10.1016/s1553-7250(08)34030-6.

31. World Medical Association. World medical association declaration of helsinki: ethical principles for medical research involving human subjects. JAMA. 2013;310(20):2191–4. pmid:24141714

Sygeplejerskers samarbejde i N. Ethical guidelines for nursing research in the Nordic countries. Revidert opptrykk ed Typotequet; 1995.

~~30.~~

32.

Formatted: Font color: Auto

Formatted: Font: Font color: Gray-87.5%

Formatted: Font: (Default) Times New Roman, 12 pt

Formatted: Font: Font color: Gray-87.5%

Formatted: Font: (Default) Times New Roman, 12 pt

Formatted: Font color: Auto

Formatted: Font: Not Bold, Font color: Gray-87.5%

Formatted: Space After: 24 pt, Line spacing: 1.5 lines, Outline numbered + Level: 1 + Numbering Style: 1, 2, 3, … + Start at: 1 + Alignment: Left + Aligned at: 0.25" + Indent at: 0.5", Pattern: Clear (White)

Response to Editor and Reviewers' comments

1. Please ensure that your manuscript meets PLOS ONE's style requirements, including those for file naming. The PLOS ONE style templates can be found at https://journals.plos.org/plosone/s/file?id=wjVg/PLOSOne_formatting_sample_main_body.pdf and https://journals.plos.org/plosone/s/file?id=ba62/PLOSOne_formatting_sample_title_authors_affiliations.pdf

Thanks for this comment, we have now revised the text to fit PLOS ONE's style

2. Your ethics statement should only appear in the Methods section of your manuscript. If your ethics statement is written in any section besides the Methods, please move it to the Methods section and delete it from any other section. Please ensure that your ethics statement is included in your manuscript, as the ethics statement entered into the online submission form will not be published alongside your manuscript.

Thanks for this comment, we have now revised the text and moved the ethics statement into the methods section

3. Please provide a complete Data Availability Statement in the submission form, ensuring you include all necessary access information or a reason for why you are unable to make your data freely accessible. If your research concerns only data provided within your submission, please write "All data are in the manuscript and/or supporting information files" as your Data Availability Statement.

Thanks for this comment, we have now revised the text

4. Please amend your authorship list in your manuscript file to include author Hammed Oladeji Mogaji

We have revised the submission data (via Edit submission) to reflect Mogaji, H.O. which is the correct author format

5. Please amend the manuscript submission data (via Edit Submission) to include author Mogaji, H.O

We have revised the submission data (via Edit submission) to reflect Mogaji, H.O. which is the correct author format

6. Please include captions for your Supporting Information files at the end of your manuscript, and update any in-text citations to match accordingly. Please see our Supporting Information guidelines for more information: http://journals.plos.org/plosone/s/supporting-information.

We have revised the text accordingly.

7. If the reviewer comments include a recommendation to cite specific previously published works, please review and evaluate these publications to determine whether they are relevant and should be cited. There is no requirement to cite these works unless the editor has indicated otherwise.

We have revised the manuscript and made relevant citations following the reviewers suggestions.

Additional Editor Comments :

C1: In addition to the reviewer's comments, I advise the authors to thoroughly revise their manuscript showing creativity so that the aim of their upcoming scoping review and the methodology used to debelop it differ substantially from two recent major reviews in the same research domain:

1. Epling et al. Lancet Infect Dis. 2025 May;25(5):e269-e279. doi: 10.1016/S1473-3099(24)00744-8.
2. Shafique et l. Syst Rev. 2024 Oct 4;13(1):253. doi: 10.1186/s13643-024-02651-9.

More details are available in the word document PONE-D-25-40772_reviewed by Mickael Essouma attached to this decision letter.

Response to Reviewer

We appreciate the reviewer's suggestion to clarify how our scoping review differs from recent reviews in this area, particularly Epling et al. (Lancet Infect Dis, 2025) – authored by M Baker, also an author of this review - and Shafique et al. (Syst Rev, 2024).

This study focuses on populations living in low income urban settings in LMICs while the Epling et al. study focused on populations marginalized by ethnicity, race, or religion - globally. These are intended to be part of a series of papers exploring adaptations of interventions for marginalized populations – with each paper focusing on a different marginalized population, while looking across diseases.

Shafique et al. synthesized community-based interventions for infectious disease control in urban informal settlements in LMICs, identifying a range of interventions. However, their review did not examine how these interventions were *adapted* to fit urban poor settings. Our study addresses this gap by focusing specifically on adaptation processes and mechanisms of delivery in urban low income settings in LMICs.

This paper is therefore distinct in two ways:
(1) it hones in on *population living in low income urban settings, in LMICs*—a group both underserved in research and highly relevant to current global health priorities;
(2) it explicitly examines *adaptation of interventions* to these settings, rather than simply listing interventions; and
It therefore builds on but does not duplicate the earlier reviews, contributing to a planned series of complementary papers that map how delivery strategies vary across different marginalized populations and contexts.

We will update both the Introduction and Discussion to make these distinctions clearer, citing the two prior reviews directly and specifying how this study extends, rather than duplicates, their contributions.

C2: Editors comments on Title within the text

- Do you wish to focus on ID outbreaks or on all types of IDs? This question stems from the fact that IDs are ubiquitous whereas the concern of IDs with regard to rapid urbanization in LMICs is most often raised in the context of ID outbreaks because ID outbreaks are more likely to occur in urban localities of so-called LMICs than in urban localities of purported developed countries.

  Focusing on ID outbreaks could also help the authors make a review differing from this 2025 scoping review (PMID: 39922209, DOI: 10.1016/S1473-3099(24)00744-8) and from this 2024 systematic review (SR; https://doi.org/10.1186/s13643-024-02651-9) especially since a SR is seen as a more useful form of evidence synthesis than a scoping review and the lag time required to update that SR may not have been reached yet (https://doi.org/10.1136/bmj.i3507). Also see https://doi.org/10.1038/s41579-021-00639-z. If you focus on ID outbreaks, then this should be specified throughout the manuscript (from the title to the end of the manuscript), not on IDs in general.

We thank the editor for this suggestion. To clarify, our review is **not restricted to outbreaks** but instead examines all infectious disease interventions delivered interpersonally, at scale, in urban poor settings in LMICs. This scope is already described in the *Introduction* (lines beginning: *"This scoping review aims to enhance understanding of how infectious disease interventions can be adapted to urban poor settings…"*) and detailed in the *Eligibility criteria* and *Table 1 – Type of intervention*.

The scope of interventions we include is consistent with that used in our earlier *Epling et al.* review, which examined delivery to racially, ethnically, and religiously marginalized populations.

2. Could you also specify, at least throughout the text (if not here as well), whether the interventions you wish to assess are mass/population-based public health interventions as it seems, in opposition to individual-level interventions? Based on Table 1, it seems you also wish to assess the outcomes of those interventions. However, this information does not appear in this title and in the introduction as expected.

Our focus is on mass population-level interventions – that include interpersonal delivery. We have revised lines 101 for better clarity to read: *"Given our focus on the delivery context, we will concentrate on a subset of population-based interventions characterized by one-on-one interactions…"*) and reiterated in *Table 1 – Inclusion criteria* under "Type of intervention."

As noted in *Table 1 – Reported outcome* and in the *Objective* section, we intend to extract information on outcomes reported (e.g., coverage, cost, prevalence, knowledge/attitudes). However, we will not be assessing interventions based on these results, as this falls outside the scope of a scoping review. A systematic review would be required for that level of synthesis, and at this stage we do not believe such evidence is available. This information will be used to report out on the types of outcomes measured – to inform the design of future studies.

3. As mentioned below, the term "poor" could be considered to be discriminative and I would remove it, especially since urban areas in LMICs most often have limited all-type resources. See BMJ Glob Health. 2022 Jun;7(6):e009704. doi: 10.1136/bmjgh-2022-009704.

We appreciate this important point and acknowledge the concern about terminology. To address it, we will replace "urban poor" with **"low-income urban communities"** throughout the manuscript. This phrasing avoids defining people by what they lack while remaining consistent with our scope. As described in our *Methods* (search strategy), our review includes related descriptors used by study authors, such as "slums," "informal settlements," and "street dwellers," to ensure comprehensiveness.

Editors comment on abstract

1. Conform to PLOS One author guidelines when formatting the revised manuscript. Accordingly, consider removing the Ethics statement in the abstract as well as sub-sections. Briefly, the abstract should look like in this article: https://doi.org/10.1371/journal.pone.0328645.

We agree and will reformat the abstract to align with PLOS ONE guidelines. Specifically, we will remove the subheadings and the ethics statement, and restructure the abstract to match the journal's style. Please see the revised abstract

Editor comments on Keywords

1. Why not just write «Urban» throughout the manuscript instead of «Urban poor»? Is it not possible to have a non-poor urban area in LMICs? See BMJ Glob Health. 2022 Jun;7(6):e009704. doi: 10.1136/bmjgh-2022-009704.

We appreciate this important point and acknowledge the concern about terminology. To address it, we will replace "urban poor" with **"low-income urban communities"** throughout the manuscript.

<u>More specific comments from Editor</u>

C1: Introduction: The introduction is currently unnecessarily very long. The greatest length of this manuscript should be reserved to the Materials and Methods section below.

We agree that the Introduction could be more concise and have revised it accordingly.

C2: Line 56-57: Could you complete this statement? Where do people moving to urban areas in LMICs come from? Or where did they come from? Rural areas of LMICs or from urban areas of high-income countries? What in their previous living areas drove them to urban areas of LMICs? Why did they not move to urban r rural areas of high-income countries?

We appreciate this thoughtful set of questions. The drivers of urban migration in LMICs vary widely by country and context. As this is the study protocol we have not yet extracted the information on setting. We therefore do not propose changes to the current text in the introduction, however we will ensure that our data extraction framework (see *Box 1* and *Data Extraction* section) is designed to capture contextual details described in the included studies, including migration patterns and community-level characteristics. This approach will allow us to better reflect the nuances of where populations come from and why, based on empirical evidence.

C3: Line 62: I would complete this sentence with a statement like: «owing to limited hygiene and therefore highly prone to the development of infectious outbreaks.» See https://doi.org/10.1038/s41579-021-00639-z

Drawing on the cover letter, the authors would also like to complete this statement with another one showing data about how rapid urbanization, increase in population density and the lack of health care infrastructures and resources drive the widespread of infectious diseases (IDs) in urban areas in LMICs.

We appreciate the suggestion. However, our review is not limited to outbreaks but is designed to capture the full range of infectious disease interventions delivered interpersonally in urban poor LMIC settings as explained above. The current text already highlights how rapid urbanization, high population density, and weak infrastructure create environments where infectious diseases thrive (see Introduction, second paragraph). To keep the Introduction concise (in line with recommendations made earlier) we prefer not to expand further.

C4: Line 63: Is this an urban area of a LMIC? If need be to take such an example alongside the above-suggested revisions, I would take the example of a prototypic highly populated urban area of a LMIC with increased ID burden (e.g., an urban area of Nigeria).

We appreciate this suggestion. We have removed these lines completely to ensure our introduction is concise as earlier suggested.

C5: Line66-67: Alongside the comment I made in line 62, this sentence would better be moved when presenting data about how rapid urbanization, increase in population density and the lack of health care infrastructures and resources drive the widespread of infectious diseases (IDs) in urban areas in LMICs. Once again, I suggest revising and completing this sentence to focus on urban areas of LMICs and on consequences of this fact with regard to the spread and control of infectious diseases.

This article may be helpful in this regard; https://doi.org/10.1038/s41579-021-00639-z

We thank the editor for this suggestion. As noted in our response to C3, our review is not restricted to outbreaks but covers a broader set of infectious disease interventions in urban poor LMIC settings. The current placement of this sentence in the Introduction is intended to show how rapid urbanization and weak infrastructure create conditions where infectious diseases thrive, thereby motivating the need for adapted interventions. While we recognize the value of framing in terms of outbreak vulnerability, shifting the focus in this way would change the scope of the review. We therefore propose to retain the current structure, while ensuring that our extraction framework captures how interventions were adapted in response to the full range of challenges faced in urban poor settings.

<u>C6: It would also be great to end this comment with a few details about why the</u>

I think this comment was cut off and we are unsure how to reply.

C7: While commenting on how urbanization in LMICs drives the spread of ID outbreaks, it would be great to mention whether and eventually why the problematic of ID control in rural areas of LMICs would be less relevant than that of ID control in urban areas of LMICs.

We thank the editor for raising this point. Most infectious disease control programs operate in rural areas, usually within existing primary health care units. However, our focus on urban poor settings does not imply that rural infectious disease control is less relevant. Rather, the delivery of interventions differs substantially between urban and rural contexts. Urban areas pose distinct challenges—including density, heterogeneity, and mobility—that require different forms of adaptation than those typically needed in rural settings. To keep the review focused and feasible, we have chosen to examine urban poor contexts, while recognizing that rural delivery remains equally important but outside the scope of this protocol.

C8: Line 69: If the authors implement the revisions suggested above, they would have highlighted how rapid urbanization with increase in population density and lack of health care infrastructures and other resources in urban areas of LMICs drive and sustain the widespread of ID outbreaks in urban areas of LMICs at this level of the introduction.

Accordingly, from line 69 to line 109, they would better focus on the current state of knowledge about interventions to control IDs in urban areas of LMICs in one paragraph, and on gaps in knowledge about interventions to control ID outbreaks in urban areas of LMICs in another paragraph. When presenting the current state of knowledge and gaps of knowledge, the authors would like to address issues such as whether the interventions are tailored to the context and culture of the researched populations and how much they contributed to the development and implementation of those interventions, including the role of community leaders and local researchers. While doing this, the authors

1. DOI: 10.1016/S1473-3099(24)00744-8
2. https://doi.org/10.1186/s13643-024-02651-9

These articles would greatly help the authors frame the needed text:

https://doi.org/10.1186/s13643-024-02651-9

https://doi.org/10.1136/bmjgh-2019-001855

Bayingana et al. Lancet Glob Health 2025; 13: e1627–35.

https://doi.org/10.1038/s41579-021-00639-z

https://doi.org/10.1371/journal.pone.0328645.

https://doi.org/10.1371/journal.pone.0317177

We thank the editor for this helpful suggestion. We agree that the Introduction could be strengthened by distinguishing more clearly between (1) what is already known about infectious disease interventions in urban LMIC contexts, and (2) the remaining gaps in knowledge. We have thus revised our third paragraph along those lines.

C9: Line 97: This is rather a scoping review protocol.

We agree with this clarification. The manuscript is indeed a protocol for a scoping review, not a completed review. This is already indicated in the title (*"...a scoping review protocol"*) and reiterated in the *Protocol Design* section (line 120 onwards).

 In the **Abstract, Materials and Methods** we wrote:
"This scoping review will follow Joanna Briggs Institute (JBI) guidelines and the PRISMA methodology adapted for scoping reviews (PRISMA-ScR)."

 In the **Protocol Design section** we wrote:
"...we will conduct a scoping review using the Joanna Briggs Institute (JBI) guidelines [24] and utilize the PRISMA extension for scoping reviews (PRISMA-ScR) guidelines for reporting [25]."

C10: Line 111-117 (on objectives): Consider revising this text using a simple language (simple sentences). It seems to me that you would like to assess whether interventions used to control the spread of ID (outbreaks) so far in urban areas of LMICs were contextually and culturally appropriate or relevant. If this is the case, it should be clearly stated. You should also consider the rationale for conducting any scoping review (map the body of evidence on a given subject) when framing the aim of this scoping review protocol (not yet the scoping review). See https://doi.org/10.1186/s12874-018-0611-x

https://doi.org/10.1371/journal.pone.0328645

https://doi.org/10.1371/journal.pone.0317177

We thank the reviewer for this observation. The objective of our review is not to assess the effectiveness of interventions in outbreaks, but rather to map how interventions have been adapted for urban poor LMIC settings. This is already reflected in our research questions (lines 98–103), but we agree the aim could be stated more clearly. We will revise the objective to emphasize that our review will (1) describe the types of adaptations reported and their relation to context, and (2) examine the methods and outcomes used to study these adaptations.

C11: Apart from the aim of the review, what is its overarching aim in terms of public/global health? What specific alert different from that sent by recent reviews on a similar topic do you wish to send to the Global Health community with the upcoming scoping review

We appreciate this question. Our overarching aim is to strengthen the evidence base on delivering infectious disease interventions equitably to marginalized populations. Whereas *Epling et al.* examined racial, ethnic, and religious marginalization, globally this protocol

focuses on urban poor settings in LMICs, where marginalization is shaped by socioeconomic and environmental conditions. By mapping adaptations in this distinct context, our review complements earlier work and extends understanding of how delivery strategies must adapt to different populations.

We have summarized and added these lines into our manuscript.

C12: Line 121-126: I suggest moving this statement to lines 113 to 117 where you highlight the aim of the upcoming scoping review, along with my comment above.

We agree and have moved the objectives into a dedicated subsection to improve clarity and alignment with PLOS ONE formatting.

C13: Line 125-126: I understand that PLOS One recommends the authors upload the PRISMA-P checklist when submitting a scoping review protocol. However, I would like to inform the authors that the PRISMA-P checklist provided as S1 is for systematic review and meta-analysis protocols whereas this is a scoping review protocol. Given the use of the JBI and PRISMA-Scr guidelines for the upcoming scoping review, I suggest also using either of both guidelines for reporting this scoping review protocol.

Thank you for catching this. We have replaced the checklist with the PRISMA-ScR version.

C14: Line 126: I understand a scoping review protocol article may serve as registration record of a scoping review (https://www.icmje.org/recommendations/). However, did the authors register this protocol article or consider registering it? See https://doi.org/10.1371/journal.pone.0328645

https://doi.org/10.1371/journal.pone.0317177

We thank the reviewer for this suggestion. In addition to publishing in PLOS ONE, we have registered the protocol on the Open Science Framework (OSF) to ensure transparency and accessibility, and have added the registration link to the Methods section.

C15: Line 128: I suggest deleting this sub-title.

We agree and have removed this sub-title to simplify the structure and improve flow.

C16: Line 129-130: This statement belongs to the first sub-section (Protocol design) of the Materials and methods section above.

We agree. We have moved this statement into the *Protocol Design* subsection to improve clarity and structure.

C17: Line 131: This sub-section should be termed “Eligibility criteria” and clearly describe inclusion and exclusion criteria in separate paragraphs.

We agree, and we have now included a section on eligibility criteria with most of the information presented in table format per your comment below.

C18: Line 135: Consider this sub-section after the Eligibility criteria sub-section.

We agree and have moved this subsection so that it follows directly after the *Eligibility criteria*. This reordering will improve the logical flow of the Methods section.

C18: Line 136: Consider replacing this statement with something like «onwards».

We have replaced the statement with onwards.

C19: Line 140: I insist is it necessary to mention the word «poor», stereotypic term which can contribute to the maintenance of social class inequalities?

See https://doi.org/10.1016/j.copsyc.2017.07.033

https://doi.org/10.1093/hrlr/ngab016

Please see response above, we have changed to use “low-income urban communities”

C20: Line 140-142: You need to provide refs here.

We have provided 3 references here

C21: Line 141-142: I understand there is no universal definition for spatial concepts or a definition specific to the so-called LMICs. However, you may consider proposing a new definition of spatial concepts based on knowledge from another group of countries such as European countries, especi ally since references 17 and 28 used to support the use of these terms (see line 142) do not seem to support the use of these search terms to refer to the term «urban». Accordingly, you could rather consider this literature, especially since there are also so-called LMICs in Europe: https://ec.europa.eu/regional_policy/sources/work/2014_01_new_urban.pdf or that from your country.

We thank the reviewer for this comment and their opinion. However, we decide to cite other articles that have used these terms in recent reviews.

C22: Line 143-151: Following the comment made just above, is this citation gaming? https://doi.org/10.1177/05390184221142218

We are unable to respond to this comment, because the lines cited do not have any citations.

C23: Line 143-151: Can you revise the preliminary search strategies presented in the S2 file and frame this sub-section as did these authors? https://doi.org/10.1371/journal.pone.0328645

We have revised our search strategies to conform to the PRISMA-Search guidelines, which specify to "Include the search strategies for each database and information source, copied and pasted exactly as run". We have also framed the sub-section as much as we can to the style of the article shared.

Rethlefsen, M.L., Kirtley, S., Waffenschmidt, S. et al. PRISMA-S: an extension to the PRISMA Statement for Reporting Literature Searches in Systematic Reviews. Syst Rev 10, 39 (2021). https://doi.org/10.1186/s13643-020-01542-z

C24: Conform to search strategy guidelines: http://dx.doi.org/10.1016/j.jclinepi.2016.01.021

DOI: dx.doi.org/10.5195/jmla.2018.283

Given that the key search concepts should emanate from the aim of the review, would you not consider these four terms as the key search concepts: ID, intervention, urban and LMICs and then develop each of those terms in the search strategies? Obviously, the LMICs mentioned in search strategies should be those of the LMIC classification chosen as highlighted when commenting on Table 1?

Thank you, we are unsure how to interpret this comment as it pertains to the provided DOIs: the first article is a guideline about peer reviewing search strategies which is recommended and not required for scoping reviews. The second article provides recommendations for searching but no requirements or guidelines. However, following your comment 23 above, we have updated the presentation of our search tables to conform to the PRISMA-S extension guidelines (see C23 above, and our S2 File).

Our PCC Framework also corresponds exactly with our search strategy: low-income urban communities (Search 2), infectious disease interventions (Search 1), and LMICs (Search 3).

C25: Line 152-164: As commented above, the sub-title should be revised to "Eligibility criteria" and the sub-section should be moved to line 128

We have made these changes as requested (See C17)

C26: Line 153-164: You get to choose whether you detail the inclusion and exclusion criteria in the text or in a table, not in both formats. Given that the table presentation appears to be more complete than the text, I would choose the table format.

Thank you for the guidance. We have summarized the inclusion and exclusion criteria description in the text to the barest minimum, and made reference to the table which is more detailed.

C27: Table 1: Have you provided all the socioeconomic and demographic characteristics of the population group to be included in the scoping review? I mean age limit, sex and gender, race, ethnicity, nationality, geographic origin, economic status

Beyond specifying that studies must focus on populations living in **urban poor settings in LMICs**, we have not imposed additional restrictions by age, sex, gender, race, ethnicity, or nationality. This is consistent with the exploratory nature of a scoping review and will allow

us to capture the widest possible range of evidence. Where such characteristics are reported in the included studies, we will extract them as part of the data charting process (see *Data Extraction* section and Box 1).

C28: Table 1 (on concept): Did you wish to write “Concept” here?

We thank the reviewer for catching this error. Yes, this was a mistake, and we will correct the heading in Table 1 to read “Concept.”

C29: Could you provide more details here about the type of (mass/population-based/public health as opposed to individual interventions which seem to be out of the scope of the upcoming review) interventions classified as preventive interventions, diagnostic interventions, and curative interventions? This means you would need three arrows for the variable “Intervention”. For each type of intervention, you would then study the intervention modalities, how they were evaluated and how they were adapted to the context and the culture.

Thank you for this helpful comment. Our scope is population-level interventions that require interpersonal delivery. We agree it will be clearer if we explicitly subdivide “Intervention” into preventive, diagnostic, and curative categories. We will also extract (i) delivery modality, (ii) adaptation to context, and (iii) study methods.

C30: Table 1 (on evaluation): Do you wish to only assess how interventions were adapted and evaluated without studying the interventions themselves? Because it does not seem that you wish to do so when going through this table.

We apologize for the confusion. We have deleted this row.

C31: Table 1: (on outcome): Outcome of intervention is a variable that should be distinct from the intervention variable and so, be addressed in another arrow of the concept part of the PCC concept.

We apologize for the confusion regarding the formatting of the table. To clarify, **intervention, information on adaptation, and reported outcomes are all distinct elements within the “Concept” domain**. In the revised Table 1, we present them as separate rows under the broader PCC category of “Concept”.

C32: Could you subdivide the outcome of intervention variable based on timeline (short-term, mid-term and long-term) and presence of absence of post-ID survival (survival outcomes versus hard outcomes [including quality of life, psychosocial well-being…)?

Please allow us to clarify, **Table 1 presents our eligibility criteria**, not our data extraction framework. For inclusion, studies must report at least one outcome, since our focus is on identifying interventions that have been delivered and adapted in practice. We will revise Table 1 to make this clearer.

C33: Table 1: on context: How will you ensure the readers believe that the countries included in the review are indeed LMICs? In other words, LMICs according to the World Bank or other classifiers? See BMJ Glob Health. 2022 Jun;7(6):e009704. doi: 10.1136/bmjgh-2022-009704. High-income countries based on which classification?

When finalizing the eligibility criteria and search strategy, we referred to The World Bank Classifications to determine which LMICs should be included. (please see ref 23). We have revised the text under objectives to reflect this.

World Bank. World Bank Country and Lending Groups. [cited March 25, 2025]. In: Knowledge Base > Country Classification [Internet]. Available from: https://datahelpdesk.worldbank.org/knowledgebase/articles/906519-world-bank-country-and-lending-groups

C34: Table 1: (under context): Do you make a difference between the arrow on countries and this one or did you wish to write "Study setting" rather than "Residence"?

We thank the reviewer for this comment. We have now revised this as Study Settings.

C35: Still regarding the setting, did you talk about the locality in a given country? Urban versus rural vs semi-urban? Bayingana et al. Lancet Glob Health 2025; 13: e1627–35. How will you define an urb an area? See for example how they defined an urb an are a in a so-called LMIC: https://doi.org/10.1016/j.jrurstud.2020.08.014.

We agree this is an important point. As noted in the Introduction (and in the reference you cite above), there is no universal definition of "urban." In our Methods (search strategy) we

already specify that a wide range of terms were used (e.g., “slum,” “informal settlement,” “street dwellers”). To make this clearer, we have revised the Context description in Table 1/Eligibility criteria to state explicitly that studies were included if authors described the setting as urban using any of these terms.

C36: Table 1: Type of study: Did you wish to say: “Study design” and “Type of publication”? Consider reframing this box to make it readable. For instance, can a mixed-methods or a qualitative study be a quasi-experimental study? What is RCT? What is the “Case studies” study design? The authors also need to reflect on the study designs most likely to address the preventive, diagnostic and curative interventions they wish to assess when framing the text of this box.

Thank you for the comments, we agree. We meant Study design and Type of publication. We have revised the text to read: Studies that are peer-reviewed and published. Can be qualitative, quantitative or mixed methods, and also descriptive or analytic.

C37: Type of publication and study design should be addressed in different arrows of the context part of the PCC concept.

Please see comments C36, AND our revised table on how we have framed it

C38: Table 1 (on type of study): Why do you exclude these types of publication records/ is it not possible to include information from reviews, opinions, textbooks (e.g., chapters) and grey literature in a scoping review which ethymologically aims to map the body of evidence on a given subject? https://doi.org/10.1186/s12874-018-0611-x

We appreciate the reviewer’s question and agree that scoping reviews can, in principle, include a broad range of evidence sources, including reviews, opinion pieces, textbooks, and grey literature. However, the aim of our review is more narrowly defined: to map how **primary research studies** have described the implementation and adaptation of infectious disease interventions in low-income urban communities. We also note that grey literature and non-empirical sources vary widely in quality and reporting detail, and a full appraisal of these materials is beyond the scope and resources of this review. We therefore restrict inclusion to original research articles that report empirical findings on interventions in these settings.

That said, we will consult relevant reviews and grey literature to ensure our search strategy is comprehensive and to contextualize findings, but they will not be included in the data charting or synthesis.

C39: Table 1 (on study subject): Consider deleting this arrow because the studied population is mentioned in the first arrow of this table and that is therefore the place where to specify that the review will focus on human subjects rather than animal subjects.

We agree, and have deleted the row

C39: Table 1: (on year of publication): Consider deleting this arrow. The information provided here belongs to “Information sources and search strategy” sub-section.

We agree, and have deleted the row

C40: Table 1: (language): Did you wish to specify “Publication language”?

Yes, thank you for catching this error. We have updated the manuscript.

C41: Line 168 (article screening): I am glad you will do this. Given that all authors are affiliated with institutions located in the USA where English language is the only official language, how are you going to ensure the accuracy of the information retrieved from publications in languages other than English? Consider specifying.

Our research team includes Portuguese, Arabic, French, and Spanish speakers and we have a wide network of colleagues that we can reach out to with other languages should they arise.

We recognize that positionality - including our own location and identities - can introduce risks of Anglocentric or Western lens bias, especially in screening and interpreting studies. It is therefore worth noting that our first author (MHO) is Nigerian living in the US, our last and corresponding author (MCB) was brought up in Peru and worked embedded in Ministries of Health in the S Pacific and Latin America, and a third author (IF) has lived experience in East Africa. This brings diversity of perspective to our study.

C42: Line 168: Consider renaming this sub-section «Study selection». Cn you make the text of this sub-section more understandable in a simpler language as in already published scoping review protocols? See https://doi.org/10.1371/journal.pone.0328645

https://doi.org/10.1371/journal.pone.0317177

C43: Line 171 (about pilot screenings): This sentence is difficult to understand for me

We have revised the text for better clarity

C44: Line 172 (about authors): Can you provide the full-spellings of these abbreviations

Those abbreviations are the author initials. However, we have removed them.

C45: Line 180: Why a PRISMA flow diagram? Will the review be a systematic or scoping one?

This is a scoping review, which also uses a PRISMA diagram - per JBI guidelines.

C46: Line 186-189: Could you better specify the variables (variables about study characteristics, variables about participant characteristics, and main review variables including the intervention variable [intervention, adaptation and evaluation of the intervention] and the outcome of intervention variable) whose data will be considered when extracting data from primary studies?

These are described in box 1. Specific nature of what is collected will depend on what is included in the published articles being reviewed.

C47: Line 191-192: Can you explain in more details how the ERIC and PRISM recommendations informed the conception of the data extraction process for the intervention variable and the outcome of intervention variable for this protocol article? I ask this question because apart from the fact that the upcoming review should be different from recent reviews on a similar topic, ERIC recommendations were made by experts from USA and Canada, but not by experts located in so-called LMICs. I do not know whether LMIC experts were involved in the recommendations recorded in ref 30 came from because I am un able to access its full-text. However, it seems to me that those

recommendations would be useful mainly to synthesize data on the implementation of interventions and the evaluation of those interventions. It is difficult for me to visualize how ref 29 will be helpful here when going through it because it seems to be helpful for assessing the feasibility and relevance of public health interventions, but I missed in the text above information about whether you are willing to assess whether those interventions were feasible before their implementation and relevant, or at least it was not so clearly stated in the introduction and preceding sub-sections of this section of the manuscript.

We have clarified above how the topic of this review is different to what has been published.

As the aim of our paper is to describe *how* interventions were adapted, it is important for us to capture in detail the intervention descriptions in the articles that we review. In this protocol, we have drawn on both the **ERIC (Expert Recommendations for Implementing Change)** and **PRISM (Practical, Robust Implementation and Sustainability Model)** frameworks to guide the design of our data extraction.

We used ERIC as a reference to ensure our extraction captures not only *what the intervention was* but also *how it was implemented and adapted* to local context. For example, in Box 1 we include extraction items such as how communities were approached, which organizations led delivery, what coordination mechanisms were used, and what facilitators/barriers were described.

PRISM similarly has broad uptake in implementation science and guided our attention to *contextual and outcome variables*. It emphasizes how interventions interact with organizational, community, and system-level factors, and how these shape sustainability. Accordingly, our extraction of outcomes goes beyond effectiveness to include implementation outcomes (coverage, uptake, fidelity), sustainability outcomes (duration, maintenance of activities), and experiential outcomes (acceptability, perceptions).

C48: Box 1: I suggest renaming this title: «Information to be extracted for the interventions and outcome of interventions variables»

Thank you for this. We have revised the title to - Information to be extracted about the interventions.

C49: Box 1: Drawing on the box title proposed above, I suggest revising this box so that the information about the interventions variable (type implemented [preventive, diagnostic and curative], implemented interventions for each type, how each intervention was adapted, and how each intervention was evaluated) and about the outcome of interventions variable are presented separately and clearly.

We have revised the box accordingly to include the intervention type, delivery modalities, and to note that outcomes measured will be recorded.

C50: If this box was built based on information from reference 30, did you get permission to modify their figure/table? Even if you did not modify one of its figure/table, you would better specify in the footnote of this box that you built it based on reference 30.

It was informed by ERIC and PRISMA frameworks and then applied to this study, it is not a modification of a table or figure in those studies.

C51: Box 1 (effectiveness): To my knowledge, these are not bits of information about outcomes of interventions, but rather about the cost-effectiveness of those interventions. I am glad you would potentially like to include information about the cost-effectiveness of those interventions because this information would

We have revised the sub-head to read outcomes and measures

C52: Line 200-205 (analysis of results): Consider thoroughly revising this sub-section, specifying how the ERIC and PRISM guidelines would eventually help synthesize the upcoming scoping review. How will data be presented? Will you stratify any data? If yes, which ones and by which variable (e.g., world region)? In brief, see how other authors framed this sub-section: https://doi.org/10.1371/journal.pone.0322753

ERIC and PRISM were used to inform the design of the extraction framework only. We have thoroughly revised this section. Please see below

We have added a sub-section to the methods section describing in detail how we will analyze the data,.

C53: Line 207: Consider removing this sub-title.

We have removed the title and the section completely

C54: Line 208-212: This text belongs to the Discussion section which should be inserted after the Materials and Methods section, and should be further elaborated, describing the strengths and pitfalls of the methodology conceived for the upcoming scoping review. The authors would like to go through the previously provided scoping review protocol articles for inspiration on how to frame the discussion section.

We have removed the title and the section completely

C55: Line 214-221: Is this realistic? When are the implementation of the se arch strategy and the elimination of duplicates due to take place? Is this timeframe realistic?

We believe our timeframe is realistic. We were able to start the screening process based on feedback received in this review and are on track.

C56: Line 221: I suggest adding the sub-sections protocol deviation, data sharing and Ethical considerations, and Patient and public involvement at the end of the Materials and Methods section. See how the others did that:

https://doi.org/10.1371/journal.pone.0322753

https://doi.org/10.1371/journal.pone.0328645

These sections has been added now

C57: Line 224: Before this statement and just after the Discussion section, consider adding a «Dissemination» statement as did these authors:
https://doi.org/10.1371/journal.pone.0328645

This section has been added

C58: References: Strengthening and update will increase the relevance of this protocol.

We have updated the references.

Reviewer 1:

C1: Abstract needs minor improvements; "Materials and methods" section is slightly dense; simplifying sentences (e.g., breaking long lists of steps) would improve readability.

Thanks for these comments. We have significantly revised the abstract and methods

C2: The conclusion could be more specific by emphasizing the expected contribution of the scoping review (e.g., identifying adaptation strategies, informing future program design).

Thanks for these comments. We have now revised the conclusion as suggested

C3: Consider consistency in terminology: "urban poor setting" could be pluralized to "urban poor settings" to match the rest of the manuscript.

Following revisions proposed by the editor, we have replaced the term "urban poor" with "low income urban communities" through the text

C4: Some sentences are long and could be simplified for clarity. For example, the sentence beginning line 60 "The rapid pace and scale of urbanization…" could be broken into two.

Thank you – we have further edited the text.

C5: Minor typographical issues: "leisMHOaniasis" line 80 appears to be a typo.

Thank you for catching this - we have revised it.

C6: The aim and objectives are currently somewhat broad and overlapping with background information, which makes them less clear. The aim uses vague language ("to understand what has been published") and would benefit from being more action-oriented, for example, "to map and synthesize evidence on adaptations of infectious disease interventions in urban poor settings in LMICs." The objectives should flow directly from the aim and clearly reflect the research questions, specifying the population, setting, and type of interventions studied. Reframing the objectives to explicitly address (1) intervention adaptations in

relation to context and (2) study designs, methods, and outcomes would improve clarity, specificity, and alignment with the research questions

Thank you for these comments. We have carefully reworded the aim, and objective of the review. Please see our new section on "Aims and objective of the scoping review”. This has helped clarify our own thinking.

C7: Some sentences are long and could be simplified for clarity, particularly in the “Information sources and search strategy” and “Data Extraction” sections. Breaking these into shorter sentences would improve readability.

Thank you – we have further edited the text.

C8: The use of the PCC (Population, Concept, Context) framework is suitable, but the description could be slightly more concise. For example, the “concept” could clearly state it focuses on interpersonal delivery adaptations rather than listing examples at this stage.

We have revised this section to ensure clarity. Please see our section on “Protocol design”.

C9: The rationale for selecting 2000 as the start year is not explicitly stated; adding a brief justification would be helpful.

We selected 2000 as the start year to ensure the review captures evidence from a time frame that is both **relevant to current global health delivery models** and **feasible for review**. Many large-scale infectious disease control initiatives in LMICs—including global immunization campaigns, scale-up of HIV/TB programs, and mass drug administration for NTDs—expanded significantly after 2000, often with increased attention to delivery in urban contexts.

We have now added this line under our inclusion criteria.

C10: Consider providing more detail on how search results will be managed across multiple languages, especially for non-English studies.

Thank you for the opportunity to clarify. As noted, we will not exclude studies based on language at this stage. If we have language capacity on our team, studies will be read in the

original language. Where this is not possible, we will use translation software (e.g., Google Translate) to produce an English version and ask someone proficient in the language to compare the translation with the original to confirm accuracy. If this is not possible, we will document the exclusion of the study and report it transparently in the review.

C11: The table is detailed and clear. However, there is a minor inconsistency: in the text, the authors state that grey literature will be included, page 9, line 160, but in the table, grey literature is listed under exclusion. Clarification is needed.

Thank you for this observation. We have corrected line 160, as grey literature will not be included.

C12: The criteria could also briefly explain how mixed interventions or multi-component studies will be handled.

In mixed interventions or multi-component study information on each intervention and component will be collected.We have now added this text under our Analysis section

C13: Box 1 is detailed but long; consider summarizing key elements in the main text and moving detailed subpoints to an appendix.

We have revised this box also following comments from the editor to add more detail and would prefer to retain it within the main file. We believe pulling out the detail of interventions and how they were implemented is critical in implementation science.

C14: Clarify whether any software or tools will be used to manage qualitative synthesis (e.g., NVivo, MAXQDA) or if this will be entirely manual.

Thanks for this comment. Qualitative synthesis will be managed manually and supported by AI. We have added more detail on how this will be done to the sub-section on analysis under results.

C15; The timeline is realistic, but adding anticipated challenges or mitigation strategies (e.g., delays in full-text retrieval or translation) could strengthen planning transparency.

We appreciate this thought. However we have been able to start the screening, implementing feedback received in this review, and we believe we are on track. If our timings change we will report.

C16; Some citations are repeated or appear as placeholders (e.g., “[17, 17,28]”); these should be checked for accuracy, line 142.

We have also revised this. Thanks for catching this error.

C17: One minor point: the funding statement contains a typographical error (“No was received” should likely read “No funding was received”). Otherwise, this section is clear and adequate.

Thanks for catching this error. We have now revised it

C18: Minor language, typographical, and consistency issues should be corrected.

We have carefully revised the manuscript and corrected typographical errors.

C19: Emphasizing action-oriented language in the objective will strengthen the manuscript’s clarity and alignment with scoping review methodology.

Thank you, we have revised this and think it is now stronger.

C20: Please check your references and be consistent

We have carefully revised the manuscript and addressed this.

C21: Line 97: The aim and objectives are currently written in a way that is somewhat wordy and overlaps with background information. Ideally, the aim should be a clear, concise statement of the overall purpose of the review, separate from the context

We have carefully revised the manuscript and addressed this.

C22: Line 97; The aim uses the phrase “to understand what has been published,” which is vague and passive. For a scoping review, it is better to use action-oriented language such as “to map,” “to synthesize,” or “to describe.”

We have carefully revised the manuscript and addressed this. Please see our comments above on this.

C23: Line 113: The objectives should flow naturally from the aim and clearly reflect the research questions. Currently, there is a slight disconnect: the aim emphasizes understanding what has been published, whereas the objectives focus on intervention adaptations, contextual factors, methods, and outcomes. Please align the objectives to your research question

We have carefully revised the manuscript and addressed this. Please see our comments above on this.

C24: Line 114: The objectives could be more specific regarding the population (urban poor), setting (LMICs), and type of interventions (interpersonal delivery-focused interventions). This specificity will make the aim and objectives sharper and more actionable. Be clear with your scope

Thank you. We have carefully revised the manuscript and addressed this.

C25; Line 114: Why re you writing research questions mixed with research objectives? Reframe the aim to a concise, action-oriented statement. Reframe the objectives to explicitly reflect the research questions.

We have carefully revised the manuscript and addressed this.

C26: Line 166. Table 1: This PCC table is too broad, make it concise

We have carefully revised the manuscript and addressed this.
