# Supplementary material for: Exploring how infectious diseases control interventions are delivered in low-income urban communities in low- and middle-income countries: A scoping review protocol
Source: PLoS One. 2025 Dec 10;20(12):e0337949. doi: 10.1371/journal.pone.0337949 (PMC12694824; doi:10.1371/journal.pone.0337949)
Supplement: Attachment — Submitted filename: Comments.docx [file pone.0337949.s003.docx]

1. Abstract needs minor improvements; “Materials and methods” section is slightly dense; simplifying sentences (e.g., breaking long lists of steps) would improve readability.
2. The conclusion could be more specific by emphasizing the expected contribution of the scoping review (e.g., identifying adaptation strategies, informing future program design).
3. Consider consistency in terminology: “urban poor setting” could be pluralized to “urban poor settings” to match the rest of the manuscript.
4. Some sentences are long and could be simplified for clarity. For example, the sentence beginning line 60 “The rapid pace and scale of urbanization…” could be broken into two.
5. Minor typographical issues: “leisMHOaniasis” line 80 appears to be a typo.
6. The aim and objectives are currently somewhat broad and overlapping with background information, which makes them less clear. The aim uses vague language (“to understand what has been published”) and would benefit from being more action-oriented, for example, “to map and synthesize evidence on adaptations of infectious disease interventions in urban poor settings in LMICs.” The objectives should flow directly from the aim and clearly reflect the research questions, specifying the population, setting, and type of interventions studied. Reframing the objectives to explicitly address (1) intervention adaptations in relation to context and (2) study designs, methods, and outcomes would improve clarity, specificity, and alignment with the research questions.
7. Some sentences are long and could be simplified for clarity, particularly in the “Information sources and search strategy” and “Data Extraction” sections. Breaking these into shorter sentences would improve readability.
8. The use of the PCC (Population, Concept, Context) framework is suitable, but the description could be slightly more concise. For example, the “concept” could clearly state it focuses on interpersonal delivery adaptations rather than listing examples at this stage.
9. The rationale for selecting 2000 as the start year is not explicitly stated; adding a brief justification would be helpful.
10. Consider providing more detail on how search results will be managed across multiple languages, especially for non-English studies.
11. The table is detailed and clear. However, there is a minor inconsistency: in the text, the authors state that grey literature will be included, page 9, line 160, but in the table, grey literature is listed under exclusion. Clarification is needed.
12. The criteria could also briefly explain how mixed interventions or multi-component studies will be handled.
13. Box 1 is detailed but long; consider summarizing key elements in the main text and moving detailed subpoints to an appendix.
14. Clarify whether any software or tools will be used to manage qualitative synthesis (e.g., NVivo, MAXQDA) or if this will be entirely manual.
15. The timeline is realistic, but adding anticipated challenges or mitigation strategies (e.g., delays in full-text retrieval or translation) could strengthen planning transparency.
16. Some citations are repeated or appear as placeholders (e.g., “[17, 17,28]”); these should be checked for accuracy, line 142.
17. One minor point: the funding statement contains a typographical error (“No was received” should likely read “No funding was received”). Otherwise, this section is clear and adequate.
18. Minor language, typographical, and consistency issues should be corrected.
19. Emphasizing action-oriented language in the objective will strengthen the manuscript’s clarity and alignment with scoping review methodology.
20. Please check your references and be consistent
