# Supplementary material for: Exploring how infectious diseases control interventions are delivered in low-income urban communities in low- and middle-income countries: A scoping review protocol
Source: PLoS One. 2025 Dec 10;20(12):e0337949. doi: 10.1371/journal.pone.0337949 (PMC12694824; doi:10.1371/journal.pone.0337949)
Supplement: Attachment — Submitted filename: PONE-D-25-40772_reviewed by Mickael Essouma.docx [file pone.0337949.s005.docx]

PLOS ONE

Exploring how infectious diseases control interventions are delivered in urban poor

community settings in low- and middle-income countries: a scoping review protocol

--Manuscript Draft--

| Manuscript Number: | PONE-D-25-40772 |
| --- | --- |
| Article Type: | Study Protocol |
| Full Title: | Exploring how infectious diseases control interventions are delivered in urban poor community settings in low- and middle-income countries: a scoping review protocol |
| Short Title: | Exploring how infectious diseases control interventions are delivered in urban poor community settings |
| Corresponding Author: | Hammed Oladeji Mogaji, Ph.D  Marian University  UNITED STATES OF AMERICA |
| Keywords: | Infectious Diseases; Urban poor; Low Middle Income Countries; Interpersonal Interventions |
| Abstract: | Background:  Infectious diseases thrive in densely populated urban poor areas where living conditions, hygiene, and access to health services are sub-optimal. Information on how to adapt infectious diseases control interventions to be effective in these settings is limited. This scoping review aims to identify specific adaptations of infectious disease interventions to urban poor setting and reported outcomes to aid understanding and potential future applications.    Materials and methods:  This scoping review will follow Joanna Briggs Institute (JBI) guidelines and the PRISMA methodology adapted for scoping reviews (PRISMA-ScR). Research questions were created using the Person (i.e., Urban poor), Concept (i.e., interpersonal infectious disease interventions), Context (i.e., low- and middle-income countries) framework. Relevant papers published after 1999 will be retrieved using a pre-defined search strategy incorporating all the PCC topics followed by a comprehensive search of MEDLINE, Embase, Global Health (all via Ovid), and Web of Science. Duplicates will be identified and removed with EndNote 20, and all articles will be uploaded into Covidence for screening in two stages using pre-defined inclusion and exclusion criteria. Quantitative and qualitative data will be extracted into Excel spreadsheets and analyzed following the Expert Recommendations for Implementing Change (ERIC) framework.    Ethics Statement:  This study does not require ethical approval because it will extract secondary data from openly accessible published articles.    Conclusion:  The findings from this review will provide understanding on how infectious diseases control interventions were adapted in urban poor settings, and associated implementation and intervention outcomes |
| Order of Authors: | M C Baker |
|  | I Fatah |
|  | C S Dorris |
|  | Hammed Oladeji Mogaji, Ph.D |
| Additional Information: |  |
| Question | Response |
| Financial Disclosure | The author(s) received no specific funding for this work. |

| Enter a financial disclosure statement that describes the sources of funding for the work included in this submission. Review the [submission guidelines](http://journals.plos.org/plosone/s/submission-guidelines#loc-financial-disclosure-statement) for detailed requirements. View published research articles from [PLOS ONE](https://journals.plos.org/plosone/) for specific examples.  This statement is required for submission and will appear in the published article if the submission is accepted. Please make sure it is accurate. |  |
| --- | --- |
| Funded studies Enter a statement with the following details: - Initials of the authors who received each award - Grant numbers awarded to each author - The full name of each funder - URL of each funder website - Did the sponsors or funders play any role in the study design, data collection and analysis, decision to publish, or preparation of the manuscript? |  |
| Did you receive funding for this work? |  |
| Competing Interests  Use the instructions below to enter a competing interest statement for this submission. On behalf of all authors, disclose any [competing interests](http://journals.plos.org/plosone/s/competing-interests) that could be perceived to bias this work—acknowledging all financial support and any other relevant financial or nonfinancial competing interests.  This statement is required for submission and will appear in the published article if the submission is accepted. Please make sure it is accurate and that any funding sources listed in your Funding Information later in the submission form are also declared in your Financial Disclosure statement.  View published research articles from | The authors have declared that no competing interests exist. |

| [PLOS ONE](http://journals.plos.org/plosone/) for specific examples. |  |
| --- | --- |
| NO authors have competing interests Enter: The authors have declared that no competing interests exist. Authors with competing interests Enter competing interest details beginning with this statement: I have read the journal's policy and the authors of this manuscript have the following competing interests: [insert competing interests here] |  |
| * typeset |  |
| Ethics Statement  Enter an ethics statement for this submission. This statement is required if the study involved:   - Human participants - Human specimens or tissue - Vertebrate animals or cephalopods - Vertebrate embryos or tissues - Field research   Write "N/A" if the submission does not require an ethics statement.  General guidance is provided below. Consult the [submission guidelines](http://journals.plos.org/plosone/s/submission-guidelines#loc-guidelines-for-specific-study-types) for detailed instructions. Make sure that all information entered here is included in the Methods section of the manuscript. | Ethics approval will not be required. Patients nor the public will not be involved at any stage of this scoping review. |

Format for specific study types

Human Subject Research (involving human

participants and/or tissue)

Give the name of the institutional review

board or ethics committee that approved the

study

•

Include the approval number and/or a

statement indicating approval of this

research

•

Indicate the form of consent obtained

(

written/oral) or the reason that consent was

not obtained (e.g. the data were analyzed

anonymously)

•

Animal Research (involving vertebrate

animals, embryos or tissues)

Provide the name of the Institutional Animal

Care and Use Committee (IACUC) or other

relevant ethics board that reviewed the

study protocol, and indicate whether they

approved this research or granted a formal

waiver of ethical approval

•

Include an approval number if one was

obtained

•

If the study involved

non-human primates

,

add

additional details

about animal welfare

and steps taken to ameliorate suffering

•

If anesthesia, euthanasia, or any kind of

animal sacrifice is part of the study, include

briefly which substances and/or methods

were applied

•

Field Research

Include the following details if this study

involves the collection of plant, animal, or

other materials from a natural setting:

Field permit number

•

Name of the institution or relevant body that

granted permission

•

Data Availability

Authors are required to make the data

underlying their research findings fully

available, without restriction.

If the manuscript reports pilot or

preliminary results, the data underlying

those results must be made available at

the time of publication. A Data Availability

N/A - No results are reported

| Statement describing where the data can be found is required at submission. Your answers to this question constitute the Data Availability Statement and will be published in the article, if accepted. When legal or ethical requirements prohibit public data sharing, authors must indicate how researchers may obtain access to the data. See the [PLOS Data Policy](https://journals.plos.org/plosone/s/data-availability) and [FAQ](https://journals.plos.org/plosone/s/data-availability#loc-data-policy-faqs) for detailed information. |  |
| --- | --- |
| Important: Stating ‘data available on request from the author’ is not sufficient. If your data are only available upon request, select ‘No’ for the first question and explain your situation in the text box. |  |
| For protocols without pilot or preliminary data, authors are strongly encouraged to state how they plan to share research data from their study when it is completed or published.  If your study includes pilot data, will they  be made fully available without restriction? |  |
| Describe where the data may be found in full sentences. If you are copying our sample text, replace any instances of XXX with the appropriate details.   - If no pilot data are reported in the manuscript, please enter the following: No datasets were generated or analysed during the current study. All relevant data from this study will be made available upon study completion. - If the manuscript reports pilot data held in a public repository, include URLs, accession numbers or DOIs. If this information will only be available after acceptance, indicate this by ticking the box below. For example: All XXX files   are available from the XXX database (accession number(s) XXX, XXX.).   - If the manuscript reports pilot data contained within the manuscript and/or | All the data resulting from this review will be extracted from published articles that are already accessible online. However, the final extraction sheet will be provided as a supplementary file. |

| Supporting Information files, enter the following:  All relevant data are within the manuscript and its Supporting Information files.   - If neither of these applies but you are able to provide details of access elsewhere, with or without limitations, please do so. For example:   Data cannot be shared publicly because of [XXX]. Data are available from the XXX Institutional Data Access / Ethics Committee (contact via XXX) for researchers who meet the criteria for access to confidential data.  The data underlying the results presented in the study are available from (include the name of the third party and contact information or URL).   - This text is appropriate if the data are owned by a third party and authors do not have permission to share the data.   For study protocols with data management or sharing plans, authors are encouraged to briefly describe in their Data Availability Statement how data they generate will be made accessible when the study is completed. For example:   - Deidentified research data will be made publicly available when the study is completed and published.   * typeset |  |
| --- | --- |
| Additional data availability information: |  |

Manuscript	[Click here to access/download;Manuscript;manuscript_.docx](https://www2.cloud.editorialmanager.com/pone/download.aspx?id=41399657&guid=fba9126e-2272-4b40-bcaf-19f0dbaa7822&scheme=1)

1 **Exploring how infectious diseases control interventions are delivered in urban**
2 **poor community settings in low- and middle-income countries: a scoping review** 3	**protocol**

4

5	Baker, M.C^1^, Fatah, I^1^, Dorris, C.S^2^, and Mogaji, H.O.^1,3*^

6

7 ^1^Department of Global Health, Georgetown University, Washington, United States of America;
8 ^2^Dahlgren Memorial Library, Georgetown University, Washington, United States of America;
9^3^Department of Behavioral and Applied Sciences, Marian University, Indianapolis, Indiana, 10 United States of America.

11

# 12 Corresponding author*: Email: mogajihammed@gmail.com ; Address: Department of

13 Behavioral and Applied Sciences, Marian University, Cold Springs Road, 46222, Indianapolis, 14 Indiana, United States of America

15

16 Baker, M.C: mcb93@georgetown.edu
17 Fatah, I: iaf4@georgetown.edu
18 Dorris, C.S: csd24@georgetown.edu
19 Mogaji, H.O: mogajihammed@gmail.com

20

# Abstract

**Background:**

Infectious diseases thrive in densely populated urban poor areas where living conditions, hygiene, and access to health services are sub-optimal. Information on how to adapt infectious diseases control interventions to be effective in these settings is limited. This scoping review aims to identify specific adaptations of infectious disease interventions to urban poor setting and reported outcomes to aid understanding and potential future applications.

**Materials and methods:**

This scoping review will follow Joanna Briggs Institute (JBI) guidelines and the PRISMA methodology adapted for scoping reviews (PRISMA-ScR). Research questions were created using the Person (i.e., Urban poor), Concept (i.e., interpersonal infectious disease interventions), Context (i.e., low- and middle-income countries) framework. Relevant papers published after 1999 will be retrieved using a pre-defined search strategy incorporating all the PCC topics followed by a comprehensive search of MEDLINE, Embase, Global Health (all via Ovid), and Web of Science.

Duplicates will be identified and removed with EndNote 20, and all articles will be uploaded into Covidence for screening in two stages using pre-defined inclusion and exclusion criteria. Quantitative and qualitative data will be extracted into Excel spreadsheets and analyzed following the Expert Recommendations for Implementing Change (ERIC) framework.

**Ethics Statement:**

This study does not require ethical approval because it will extract secondary data from openly accessible published articles.

**Conclusion:**

The findings from this review will provide understanding on how infectious diseases control interventions were adapted in urban poor settings, and associated implementation and intervention outcomes

**Keywords**:

Infectious diseases, Urban poor, Low Middle-Income Countries, Interpersonal Interventions **Introduction**

Urbanization has accelerated rapidly in this century, with approximately 57% of the world’s population now living in cities [1], and the global urban population projected to reach 6.25 billion by 2050 [2]. This growth is driven by multiple factors, including government policies on public housing, land use, property ownership and potential for economic opportunities [3]. Cities that contribute to the largest share of national GDPs are where private sector jobs and consumers are increasingly concentrated [4]. To date, the largest expansion of urban populations has been, and is projected to continue to be, focused on low and middle-income countries (LMICs) [5]. The rapid pace and scale of urbanization has resulted in significant pressure on available infrastructure and resources, with expansion into areas previously considered uninhabitable [4]. For instance, more than 75,000 km^2^ of new urban land, equivalent to about 50 times the area of Greater London, has been developed in areas prone to severe flooding for human habitation [4]. These areas, often home to informal settlements, are characterized by inadequate housing conditions, poor health and safety, and fewer work opportunities [6]. Over a billion people currently reside in informal settlements in urban areas, with numbers projected to rise by more than 300% by 2030 [7].

The impact of urbanization on the spread of infectious diseases is highly contextualized. On one hand, urbanization can reduce mortality and morbidity caused by infectious agents in areas where living conditions, hygiene, and access to health services are optimal [8]. On the other hand, rapid urbanization has also resulted in the growth of informal settlements [9], where infectious diseases thrive. Factors associated with the spread of infectious diseases in urban, especially urban poor settings, have been summarized by Alirol et al [8] and Neiderud et al [10]. High population density allows air-borne infections like influenza, measles, and *Mycobacterium tuberculosis* to rapidly spread [11]. Inadequate water and sanitation services contribute to diarrhoeal diseases caused by cholera and *E. coli* [12,13], to the transmission of soil-transmitted helminthiasis and other intestinal parasites [14], and to the proliferation of rodents such as rats which can carry *Leptospira interrogans* [15]*.* Accumulated waste can provide breeding sites for vectors like sand flies that carry parasites causing leisMHOaniasis and of *Aedes* mosquitoes that are vectors for dengue, yellow fever, and chikungunya [16].

While many interventions have proven to be effective in preventing infectious diseases (e.g. WASH, health education, community-based screening, socio-economic support, communitybased vector control, behavior change, social mobilization, and electronic(e-) and mobile(m-) health) [17], implementation failure in urban settings has also been widely documented. For example, immunization programs have reported challenges in reaching urban dwellers due to working with large populations that are transient in nature (including seasonal workers), with inconsistent employment patterns, and multicultural beliefs and practices [18]. Similarly, mass drug administrations (MDA) for controlling neglected tropical diseases (NTDs) in Africa and the Americas, also report lower coverage in urban settings due to the heterogeneity that exists within urban environments [19,20]. The success of immunization, contraception, and antenatal care services was also reported to be sub-optimal in urban slum areas of Chandigarh in the North of India. [18, 21]. Till date, information on how to adapt interventions to be effective in urban settings is limited [19,20].

This scoping review aims to enhance understanding of how infectious disease interventions can be adapted to urban poor settings by identifying specific adaptations, implementation and intervention outcomes, and examining research methods applicable to future studies. Given our focus on the delivery context, we will concentrate on a subset of interventions characterized by one-on-one interactions between a distributor and a recipient within the community. This delivery mode— often referred to as ‘campaigns’—is commonly used for vaccinations, mass drug administration (MDA), screening and test-and-treat initiatives, and distribution of products such as bed nets, condoms, and facemasks. In contrast, we will exclude interventions that do not rely on interpersonal interaction, such as environmental modifications (e.g. sewage and trash collection as a potential strategy to reduce rodents or spraying for vector control) and mass communication or health education for behavior change. This focus aligns with the methodological approach taken in a previous scoping review examining adaptations for reaching ethnically, racially, and religiously marginalized populations [22]

# Objective of the Scoping review

The main objective of this review is to understand what has been published on adapting the delivery of infectious disease interventions in urban poor settings in LMICs. We seek to explore two primary research questions: (1) What adaptations to the interventions were tested and how did these relate to the context?, and (2) How has this topic been studied, including methods used and outcomes measured?

**Materials and Methods**

# Protocol Design

Scoping reviews use standardized methods, taking a systematic approach, and are the methodology of choice when the aim is to get a broad overview of a potentially diverse body of literature [24]. Hence, we will conduct a scoping review using the Joanna Briggs Institute (JBI) guidelines [24] and utilize the PRISMA extension for scoping reviews (PRISMA-ScR) guidelines for reporting

[25]. This protocol has been written following the Preferred Reporting Items for Systematic Review and Meta-Analysis Protocols (PRISMA-P) (See S1 File).

# Methodological Framework

This scoping review will use the Person, Concept, Context (PCC) framework to guide the formation of research questions and search strategies [26]. Our population will include those living in urban poor communities. The concept will be adaptations of infectious disease interventions characterized by one-on-one interactions between a distributor and a recipient within the community. The context will be low-middle-income countries.

# Information sources and search strategy

Relevant articles published from the year 2000 until [the date of search] will be retrieved from MEDLINE, Embase, Global Health (all via Ovid), and Web of Science with the goal of identifying articles from both medical and social sciences fields. The search strategy will incorporate both database-specific subject headings and keywords that represent all three PCC topics: urban poor, infectious disease interventions, and LMICs. As there is no universally accepted global definition of spatial concepts, we will use search terms like “urban,” “peri-urban,” “city,” “municipality,”

“urbanization,” and “township.” used in other literature reviews on this subject. [17,28]. Terms related to urban poor populations including slums, informal settlements, street dwellers, will also be used to select for the population of interest. As our concept focuses on adapting the type of infectious disease interventions that require interpersonal interaction between those delivering a health product and those receiving it, in a community setting, we will develop search terms related to vaccine, mass drug administration, and mass screening or testing for infectious diseases. We will develop the search strategy by running initial search terms in Ovid MEDLINE and test the results to obtain an optimal balance between allowing for a broad scope and feasibility. We will then translate the search terms for the remaining databases. [see S2 File]

# Inclusion and Exclusion Criteria

We will include peer-reviewed studies published from the year 2000 onwards that focus on individuals residing in urban poor areas of low- and middle-income countries (LMICs). Eligible studies must involve infectious diseases control interventions requiring interpersonal contact in a community setting, such as immunization, mass drug administration, screening, contact tracing, or distribution of health commodities. Studies must report on efforts made to adapt interventions to increase access to our defined population (providing at least a minimum description of the intervention), include some form of evaluation of the intervention and report on at least one outcome. Only community-based studies involving human subjects will be included. Studies other than literature reviews, editorials, opinion pieces, grey literature will be included. All languages will be included as the authors spoke several languages among them and other languages could be rapidly translated using online software. Articles will be excluded if they do not meet the inclusion criteria. All inclusion and exclusion criteria are summarized in Table 1.

165

166	**Table 1:** Inclusion and exclusion criteria

| **Framework** | **Items** | **Inclusion** | **Exclusion** |
| --- | --- | --- | --- |
| Population | Individuals | Studies concerning individuals residing in urban poor areas | Studies that are not specific to individuals residing in urban poor areas |
| Context | Type of intervention | Studies on Interventions that require interpersonal contact in a community setting, specifically: immunizations, mass drug administration/ deworming, screening, contact tracing, distribution of commodities (e.g. condoms, facemasks, bed nets). | Studies on Interventions that do not rely on one-one interaction - either  reaching many people simultaneously (e.g. mass communication health education campaigns targeting behavior change) or interventions that rely on environmental modifications (e.g. spraying for vector control or water and sanitation projects). |
|  | Information on adaptation | Studies that describe how the intervention was adapted to urban poor setting, even if very limited. | Studies that did not describe how the intervention was adapted to urban poor setting |
|  | Information on Evaluation | Evaluation studies about the intervention in urban poor setting (this can be tested as part of or before the research study) | Formative studies designed to collect information to inform the design of adapted interventions, prior to any adaptations having been made. |
|  | Reported outcome | Studies that report at least one implementation or intervention outcome (e.g. coverage, cost, prevalence, knowledge/  attitudes, etc | Studies that did not report any implementation or intervention outcome e.g. coverage, cost, prevalence, knowledge/ attitudes, etc |
| Context | Countries | Studies implemented in Low- and Middle-Income Countries (LMICs) | Studies implemented in High Income Countries (HICs) |
|  | Residence | Studies implemented at the community level | Studies that are hospital-based |
|  | Type of study | Peer-reviewed Research studies (e.g. retrospective case studies, quasi-experimental e.g. before and after studies; RCTs) Can be qualitative or quantitative or mixed methods. | Literature reviews/textbooks/ dissertations/ editorials/ opinion pieces/ grey literature |
|  | Study subject | Studies about Human species | Studies about non-Human species |
|  | Year of publication | Studies published from 2000 till date | Studies published prior 2000 |
|  | Language | Studies reported in all languages |  |

167

# 168 Article screening process

169 All retrieved articles will be uploaded into Covidence for screening after duplicates have been removed. Duplicates will be identified and removed with EndNote 20 (Clarivate Analytics, Philadelphia, PA). To ensure clarity of the inclusion and exclusion criteria we will perform two

172 pilot screenings of 200 and 50 randomly selected articles with BMC, FI, and HOM. These articles 173 will each be screened independently by two reviewers and discrepancies discussed. We will 174 proceed with full title and abstract review once conflict is below 5% of every 100 articles screened.

175 In the first stage of full screening, we will first review 100 of titles and abstracts against inclusion 176 and exclusion criteria. To ensure calibration between the reviewers and to increase quality, all

articles will be double reviewed at this stage. Conflicts that arise at this stage of screening will be resolved initially through discussions between screeners, with remaining conflicts discussed with BMC and MHO. Sources included in the title and abstract stage of screening will undergo full-text review, with the same eligibility criteria used to determine relevance. A PRISMA flow diagram will be created to summarize the results of the search and reasons for exclusion. Subsequently, reviewers will continue to meet every 1-2 weeks to discuss the studies, including any article which there is uncertainty on whether to include it.

# Data Extraction

Quantitative and qualitative data will be extracted by researchers using a data extraction form in

Microsoft excel, including data on the year and language of the publication, the study design (including research question, type of data collected, and outcomes measured) and on the implementation of the intervention (including the context, adaptations made, and outcomes). The extraction framework related to the intervention (see Box 1) was developed with reference to concepts identified in Expert Recommendations for Implementing Change (ERIC) framework [29] and the Practical, Robust Implementation and Sustainability Model (PRISM) [30]. The former framework has been used in a similar literature review focused on different population groups [22], and the latter is used widely in implementation research [30]. The data will initially be abstracted independently and then reviewed by BMC and MHO.

**Box 1:** Intervention specific elements we will include in extraction framework

**Intervention:**

- What the intervention is e.g. bednet distribution, health education campaign, screening -	Why the intervention was needed
- Intervention goal, including disease(s) targetted **Context:**
- Where implemented: Countries, sub-country, number of sites
- Community setting environment: socio- economic, geography, political
- Population receiving intervention: who, perceptions on the intervention and on organizations delivering the intervention
- Why this setting was selected

**Implementation and adaptation made to the context:**

- How did decisions made to do things a specific way relate to the context?
- How the community was initially approached and how they were engaged during each stage of the study: design, implementation, and review
- What organization(s) led delivery of the intervention, who within these organizations, and what were their perceptions on intervention and the community?
- Who were other stakeholders, what was their role, and why were they engaged? -	What processes were put in place to coordinate between all stakeholders?
- Adoption / uptake of intervention e.g. number of workshops, amount of product delivered, etc
- What worked well and why (include facilitators)
- What did not work and why (include barriers and if/how overcome) -	Cost and funding **Outcomes:**
- Effectiveness: e.g. reduced costs, reduced disease, increased knowledge, coverage
- Sustainability: how long was the interventions delivered for and what mention was there of maintaining activities/ outcomes long term.

# Analysis of results

Extracted data will be analyzed by counting frequency and qualitatively by reading through the summary data extracted, writing synthesized narratives on each element, and looking for patterns such as common themes and context specific differences. The research team will meet regularly during the analysis phase to share draft findings and ask probing questions that will prompt deeper analysis.

# Expected outcome of the study

This study aims to improve understanding of how infectious disease interventions are adapted for urban poor settings. It is expected to identify effective adaptation strategies, key implementation and intervention outcomes, and the facilitators and barriers influencing success. The findings will also generate practical recommendations to inform future research and guide program design and delivery in similar contexts.

# Timeline of the scoping review

The scoping review will be conducted over a six-month period, from August 2025 to January 2026. The development of the search strategy is expected to take place in August **2025**, followed by the initial screening of titles and abstracts within the same month. Full-text screening will occur in September, followed by data extraction in October. Data analysis and interpretation will take place in November. The results from the scoping review will be written as a manuscript in December, and abstracts prepared for submission to conferences. Fully revised manuscript will be submitted to an openaccess journal for publication consideration.

# Supplementary Information

S1 File: Completed PRISMA-P 2015 checklist

S2 File: PRISMA numbers from Search Strategy

**Declarations**

# Authors’ contributions

BMC conceptualized the study; BMC, MHO, FI prepared the protocol; BMC, MHO, DCS designed the search strategy; DCS constructed the search framework; MHO prepared the first version for the manuscript. All authors revised and approved the manuscript. BMC is the guarantor of the review

# Ethics approval and consent to participate

Ethics approval will not be required. Patients nor the public will not be involved at any stage of this scoping review.

**Consent for publication**

Not applicable

# Availability of data and materials

All the data resulting from this review will be extracted from published articles that are already accessible online. However, the final extraction sheet will be provided as a supplementary file.

**Funding**

No funding was received for this study.

# References

1. World	Bank	Group. World	Urbanization	Prospects,	United	Nations. <https://data.worldbank.org/indicator/SP.URB.TOTL.IN.ZS>

2. United Nations Department of Economic and Social Affairs. 2014 revision of the World Urbanization Prospects. [https://www.un.org/en/development/desa/publications/2014revision-world-urbanization-prospects.html](https://www.un.org/en/development/desa/publications/2014-revision-world-urbanization-prospects.html)

3. Yu D, Li X, Yu J, Shi X, Liu P, Tian P. Whether Urbanization Has Intensified the Spread of Infectious Diseases—Renewed Question by the COVID-19 Pandemic. *Front Public Health*. 2021;9. doi: 10.3389/fpubh.2021.699710.

4. World	Bank	Group.	Urban	Development. <https://www.worldbank.org/en/topic/urbandevelopment/overview>

5. Gunaratna, KL, Eduardo Cesar LM, Yansui L, Liana R, Akiko O, Obudho R, Berma M,

Sumanm KK, Jan Q, Harrison P, Maharaj B, Knowles C, Sandro G, Seto KC, Ndilila FM. IAP Statement on Implications of Urbanization in Low- and- Middle- Income Countries. The Inter Academy Partnership, 2022.

6. United Nations. Rapid urbanization and population growth are outpacing the construction of adequate and affordable housing. <https://unstats.un.org/sdgs/report/2019/goal-11/>

7. Cociña, C and Landesman, T. Better cities are possible: transforming informal settlements on a warming planet. 2025 IIED, London.

8. Alirol E, Getaz L, Stoll B, Chappuis F, Loutan L. Urbanisation and infectious diseases in a globalised world. *Lancet Infect Dis*. 2011;11: 131–141. doi: 10.1016/S1473-

3099(10)70223-1.

9. Alene, ET. Determinant factors for the expansion of informal settlement in Gondar city, Northwest Ethiopia. *J. Urban Manag.* 2022; 11:321-337

10. Neiderud C. How urbanization affects the epidemiology of emerging infectious diseases. *Infect Ecol Epidemiol*. 2015;5: 27060. doi: 10.3402/iee.v5.27060.

11. Fu H, Zhu C. The impact of population influx on infectious diseases - from the mediating effect of polluted air transmission. *Front Public Health.* 2024 Jul 30;12:1344306. doi:

10.3389/fpubh.2024.1344306. Erratum in: Front Public Health. 2024 Sep 03;12:1480516.

doi: 10.3389/fpubh.2024.1480516. PMID: 39139663; PMCID: PMC11319163.

12. Saba D, Ercan A, Senkaya I, Gebitekin C, Ozkan H. The impact of water, sanitation and hygiene on key health and social outcomes: review of evidence, 2016. UN Children's Fund, New York.

13. Liu Q, Deng J, Yan W, Qin C, Du M, Wang Y, et al. Burden and trends of infectious disease mortality attributed to air pollution, unsafe water, sanitation, and hygiene, and nonoptimal temperature globally and in different socio-demographic index regions. *Glob Health Res Policy*. 2024;9: 23. doi: 10.1186/s41256-024-00366-x.

14. Mogaji HO, Olamiju FO, Oyinlola F, Achu I, Adekunle ON, Udofia LE, Edelduok EG,

Yaro CA, Oladipupo OO, Kehinde AY, Oyediran F, Aderogba M, Makau-Barasa LK, Ekpo UF. Prevalence, intensity and risk factors of soil-transmitted helminthiasis after five effective rounds of preventive chemotherapy across three implementation units in Ondo

State,	Nigeria.	*PLoS	Negl	Trop	Dis*.	2025	Jan	6;19(1):e0012533.	doi:

10.1371/journal.pntd.0012533.

15. Khalil H, Santana R, de Oliveira D, Palma F, Lustosa R, Eyre MT, et al. Poverty, sanitation, and Leptospira transmission pathways in residents from four Brazilian slums. *PLoS Negl Trop Dis.* 2021;15: e0009256. doi: 10.1371/journal.pntd.0009256.

16. Krystosik A, Njoroge G, Odhiambo L, Forsyth JE, Mutuku F, LaBeaud AD. Solid Wastes Provide Breeding Sites, Burrows, and Food for Biological Disease Vectors, and Urban Zoonotic Reservoirs: A Call to Action for Solutions-Based Research. Front Public Health. 2020 Jan 17;7:405. doi: 10.3389/fpubh.2019.00405.

17. Shafique S, Bhattacharyya DS, Nowrin I, Sultana F, Islam MR, Dutta GK, et al. Effective community-based interventions to prevent and control infectious diseases in urban informal settlements in low- and middle-income countries: a systematic review. *Syst Rev*. 2024;13. doi: 10.1186/s13643-024-02651-9.

18. Zimba B, Mpinganjira S, Msosa T, Bickton FM. The urban-poor vaccination: Challenges and strategies in low-and-middle income countries. *Human Vaccines &amp; Immunotherapeutics*. 2024;20. doi: 10.1080/21645515.2023.2295977.

19. Adams AM, Vuckovic M, Birch E, Brant TA, Bialek S, Yoon D, et al. Eliminating Neglected Tropical Diseases in Urban Areas: A Review of Challenges, Strategies and

Research Directions for Successful Mass Drug Administration. *Trop Med Infect Dis.* 2018;3: 122. doi: 10.3390/tropicalmed3040122.

20. Olamiju FO, Nebe O, Mogaji H, Abdus-Salam I, Jenrola L, Marcus AJ, et al. The first mass drug administration campaign for schistosomiasis control in Lagos, Nigeria: lessons for future control programs. *Front in Trop Dis*. 2023;4. doi: 10.3389/fitd.2023.1094320.

21. Gupta M, Thakur JS, Kumar R. Reproductive and child health inequities in Chandigarh Union Territory of India. *J Urban Health*. 2008;85: 291–299. doi: 10.1007/s11524-0079250-y.

22. Epling SWM, Bjork AM, Martinez Cruz L, Baker MC. Approaches to increase access to community-based infectious disease control for ethnically, racially, and religiously marginalised populations: a scoping review. *Lancet Infect Dis*. 2025;25: e269–e279. doi:

10.1016/S1473-3099(24)00744-8.

23. The World Bank. World Bank Country and Lending Groups. World Bank Data Help Desk. Available: [https://datahelpdesk.worldbank.org/knowledgebase/articles/906519-worldbank-country-and-lending-groups.](https://datahelpdesk.worldbank.org/knowledgebase/articles/906519-world-bank-country-and-lending-groups)

24. Peters MDJ, Godfrey C, McInerney P, Khalil H, Larsen P, Marnie C, et al. Best practice guidance and reporting items for the development of scoping review protocols. *JBI Evid Synth*. 2022;20: 953–968. doi: 10.11124/JBIES-21-00242.

25. Tricco AC, Lillie E, Zarin W, O'Brien KK, Colquhoun H, Levac D, et al. PRISMA Extension for Scoping Reviews (PRISMA-ScR): Checklist and Explanation. *Ann Intern Med.* 2018;169: 467–473. doi: 10.7326/M18-0850.

26. Pollock D, Peters MDJ, Khalil H, McInerney P, Alexander L, Tricco AC, Evans C, de Moraes ÉB, Godfrey CM, Pieper D, Saran A, Stern C, Munn Z. Recommendations for the extraction, analysis, and presentation of results in scoping reviews. *JBI Evid Synth*. 2023 Mar 1;21(3):520-532. doi: 10.11124/JBIES-22-00123. PMID: 36081365.

27. Turley R, Saith R, Bhan N, Rehfuess E, Carter B. Slum upgrading strategies involving physical environment and infrastructure interventions and their effects on health and socioeconomic outcomes. *Cochrane Database Syst Rev*. 2013;(1):CD010067. doi: CD010067. doi: 10.1002/14651858.CD010067.pub2.

28. Henson RM, Ortigoza A, Martinez-Folgar K, Baeza F, Caiaffa W, Vives Vergara A, et al. Evaluating the health effects of place-based slum upgrading physical environment interventions: A systematic review (2012–2018). *Soc Sci Med*. 2020;261: 113102. doi:

10.1016/j.socscimed.2020.113102.

29. Waltz TJ, Powell BJ, Matthieu MM, Damschroder LJ, Chinman MJ, Smith JL, et al. Use of concept mapping to characterize relationships among implementation strategies and assess their feasibility and importance: results from the Expert Recommendations for Implementing Change (ERIC) study. *Implement Sci*. 2015;10: 109. doi: 10.1186/s13012015-0295-0.

377

378 30. Feldstein AC, Glasgow RE. A practical, robust implementation and sustainability model 379 (PRISM) for integrating research findings into practice. *Jt Comm J Qual Patient Saf.* 380 2008;34: 228–243. doi: 10.1016/s1553-7250(08)34030-6.

381

[Click here to access/download](https://www2.cloud.editorialmanager.com/pone/download.aspx?id=41399658&guid=3b52c3aa-9370-48d5-ae46-71ce29083146&scheme=1)

[Supporting Information S1 File.docx](https://www2.cloud.editorialmanager.com/pone/download.aspx?id=41399658&guid=3b52c3aa-9370-48d5-ae46-71ce29083146&scheme=1)

[Click here to access/download](https://www2.cloud.editorialmanager.com/pone/download.aspx?id=41399659&guid=d55c47b8-5da7-47d1-819d-b3f825c2fb0c&scheme=1)

[Supporting Information S2 File.docx](https://www2.cloud.editorialmanager.com/pone/download.aspx?id=41399659&guid=d55c47b8-5da7-47d1-819d-b3f825c2fb0c&scheme=1)
