# Supplementary material for: Exploring how infectious diseases control interventions are delivered in low-income urban communities in low- and middle-income countries: A scoping review protocol
Source: PLoS One. 2025 Dec 10;20(12):e0337949. doi: 10.1371/journal.pone.0337949 (PMC12694824; doi:10.1371/journal.pone.0337949)
Supplement: Attachment — Submitted filename: Response to Editor and Reviewers_Oct 6 2025.docx [file pone.0337949.s008.docx]

**Response to Editor and Reviewers’ comments**

1. Please ensure that your manuscript meets PLOS ONE's style requirements, including those for file naming. The PLOS ONE style templates can be found at <https://journals.plos.org/plosone/s/file?id=wjVg/PLOSOne_formatting_sample_main_body.pdf> and <https://journals.plos.org/plosone/s/file?id=ba62/PLOSOne_formatting_sample_title_authors_affiliations.pdf>

Thanks for this comment, we have now revised the text to fit PLOS ONE’s style

2. Your ethics statement should only appear in the Methods section of your manuscript. If your ethics statement is written in any section besides the Methods, please move it to the Methods section and delete it from any other section. Please ensure that your ethics statement is included in your manuscript, as the ethics statement entered into the online submission form will not be published alongside your manuscript.

Thanks for this comment, we have now revised the text and moved the ethics statement into the methods section

3. Please provide a complete Data Availability Statement in the submission form, ensuring you include all necessary access information or a reason for why you are unable to make your data freely accessible. If your research concerns only data provided within your submission, please write "All data are in the manuscript and/or supporting information files" as your Data Availability Statement.

Thanks for this comment, we have now revised the text

4. Please amend your authorship list in your manuscript file to include author Hammed Oladeji Mogaji

We have revised the submission data (via Edit submission) to reflect Mogaji, H.O. which is the correct author format

5. Please amend the manuscript submission data (via Edit Submission) to include author Mogaji, H.O

 We have revised the submission data (via Edit submission) to reflect Mogaji, H.O. which is the correct author format

6. [Please include captions for your Supporting Information files at the end of your manuscript, and update any in-text citations to match accordingly. Please see our Supporting Information guidelines for more information: http://journals.plos.org/plosone/s/supporting-information.](http://track.editorialmanager.com/CL0/http:%2F%2Fjournals.plos.org%2Fplosone%2Fs%2Fsupporting-information./1/010f0198d4e45b80-b1f60f12-36de-4051-b0d0-372c7804b820-000000/oTy8guNfdTMDv_HzjZb6SpEZixBVJ74GH3a0MMwPkYE=225)

We have revised the text accordingly.

7. If the reviewer comments include a recommendation to cite specific previously published works, please review and evaluate these publications to determine whether they are relevant and should be cited. There is no requirement to cite these works unless the editor has indicated otherwise.

We have revised the manuscript and made relevant citations following the reviewers suggestions.

**Additional Editor Comments :**

C1: In addition to the reviewer's comments, I advise the authors to thoroughly revise their manuscript showing creativity so that the aim of their upcoming scoping review and the methodology used to debelop it differ substantially from two recent major reviews in the same research domain:

1. Epling et al. Lancet Infect Dis. 2025 May;25(5):e269-e279. doi: 10.1016/S1473-3099(24)00744-8.
2. Shafique et l. Syst Rev. 2024 Oct 4;13(1):253. doi: 10.1186/s13643-024-02651-9.

More details are available in the word document PONE-D-25-40772_reviewed by Mickael Essouma attached to this decision letter.

Response to Reviewer

We appreciate the reviewer’s suggestion to clarify how our scoping review differs from recent reviews in this area, particularly Epling et al. (Lancet Infect Dis, 2025) – authored by M Baker, also an author of this review - and Shafique et al. (Syst Rev, 2024).

This study focuses on populations living in low income urban settings in LMICs while the Epling et al. study focused on populations marginalized by ethnicity, race, or religion - globally. These are intended to be part of a series of papers exploring adaptations of interventions for marginalized populations – with each paper focusing on a different marginalized population, while looking across diseases.

Shafique et al. synthesized community-based interventions for infectious disease control in urban informal settlements in LMICs, identifying a range of interventions. However, their review did not examine how these interventions were *adapted* to fit urban poor settings. Our study addresses this gap by focusing specifically on adaptation processes and mechanisms of delivery in urban low income settings in LMICs.

This paper is therefore distinct in two ways:
(1) it hones in on *population living in low income urban settings, in* *LMICs*—a group both underserved in research and highly relevant to current global health priorities;
(2) it explicitly examines *adaptation of interventions* to these settings, rather than simply listing interventions; and
It therefore builds on but does not duplicate the earlier reviews, contributing to a planned series of complementary papers that map how delivery strategies vary across different marginalized populations and contexts.

We will update both the Introduction and Discussion to make these distinctions clearer, citing the two prior reviews directly and specifying how this study extends, rather than duplicates, their contributions.

**C2: Editors comments on Title within the text**

- Do you wish to focus on ID outbreaks or on all types of IDs? This question stems from the fact that IDs are ubiquitous whereas the concern of IDs with regard to rapid urbanization in LMICs is most often raised in the context of ID outbreaks because ID outbreaks are more likely to occur in urban localities of so-called LMICs than in urban localities of purported developed countries.

Focusing on ID outbreaks could also help the authors make a review differing from this 2025 scoping review (PMID: 39922209, DOI: [10.1016/S1473-3099(24)00744-8](https://doi.org/10.1016/s1473-3099(24)00744-8)) and from this 2024 systematic review (SR; <https://doi.org/10.1186/s13643-024-02651-9>) especially since a SR is seen as a more useful form of evidence synthesis than a scoping review and the lag time required to update that SR may not have been reached yet (<https://doi.org/10.1136/bmj.i3507>). Also see <https://doi.org/10.1038/s41579-021-00639-z>. If you focus on ID outbreaks, then this should be specified throughout the manuscript (from the title to the end of the manuscript), not on IDs in general.

We thank the editor for this suggestion. To clarify, our review is **not restricted to outbreaks** but instead examines all infectious disease interventions delivered interpersonally, at scale, in urban poor settings in LMICs. This scope is already described in the *Introduction* (lines beginning: *“This scoping review aims to enhance understanding of how infectious disease interventions can be adapted to urban poor settings…”*) and detailed in the *Eligibility criteria* and *Table 1 – Type of intervention*.

The scope of interventions we include is consistent with that used in our earlier *Epling et al.* review, which examined delivery to racially, ethnically, and religiously marginalized populations.

2. Could you also specify, at least throughout the text (if not here as well), whether the interventions you wish to assess are mass/population-based public health interventions as it seems, in opposition to individual-level interventions? Based on Table 1, it seems you also wish to assess the outcomes of those interventions. However, this information does not appear in this title and in the introduction as expected.

Our focus is on mass population-level interventions – that include interpersonal delivery. We have revised lines 101 for better clarity to read: *“Given our focus on the delivery context, we will concentrate on a subset of population-based interventions characterized by one-on-one interactions…”* ) and reiterated in *Table 1 – Inclusion criteria* under “Type of intervention.”

As noted in *Table 1 – Reported outcome* and in the *Objective* section, we intend to extract information on outcomes reported (e.g., coverage, cost, prevalence, knowledge/attitudes). However, we will not be assessing interventions based on these results, as this falls outside the scope of a scoping review. A systematic review would be required for that level of synthesis, and at this stage we do not believe such evidence is available. This information will be used to report out on the types of outcomes measured – to inform the design of future studies.

3. As mentioned below, the term “poor” could be considered to be discriminative and I would remove it, especially since urban areas in LMICs most often have limited all-type resources. See BMJ Glob Health. 2022 Jun;7(6):e009704. doi: 10.1136/bmjgh-2022-009704.

We appreciate this important point and acknowledge the concern about terminology. To address it, we will replace “urban poor” with **“low-income urban communities”** throughout the manuscript. This phrasing avoids defining people by what they lack while remaining consistent with our scope. As described in our *Methods* (search strategy), our review includes related descriptors used by study authors, such as “slums,” “informal settlements,” and “street dwellers,” to ensure comprehensiveness.

**Editors comment on abstract**

1. Conform to PLOS One author guidelines when formatting the revised manuscript. Accordingly, consider removing the Ethics statement in the abstract as well as sub-sections. Briefly, the abstract should look like in this article: <https://doi.org/10.1371/journal.pone.0328645>.

We agree and will reformat the abstract to align with PLOS ONE guidelines. Specifically, we will remove the subheadings and the ethics statement, and restructure the abstract to match the journal’s style. Please see the revised abstract

**Editor comments on Keywords**

1. Why not just write «Urban» throughout the manuscript instead of «Urban poor»? Is it not possible to have a non-poor urban area in LMICs? See BMJ Glob Health. 2022 Jun;7(6):e009704. doi: 10.1136/bmjgh-2022-009704.

We appreciate this important point and acknowledge the concern about terminology. To address it, we will replace “urban poor” with **“low-income urban communities”** throughout the manuscript.

**More specific comments from Editor**

C1: Introduction: The introduction is currently unnecessarily very long. The greatest length of this manuscript should be reserved to the Materials and Methods section below.

We agree that the Introduction could be more concise and have revised it accordingly.

C2: Line 56-57: Could you complete this statement? Where do people moving to urban areas in LMICs come from? Or where did they come from? Rural areas of LMICs or from urban areas of high-income countries? What in their previous living areas drove them to urban areas of LMICs? Why did they not move to urban r rural areas of high-income countries?

We appreciate this thoughtful set of questions. The drivers of urban migration in LMICs vary widely by country and context. As this is the study protocol we have not yet extracted the information on setting. We therefore do not propose changes to the current text in the introduction, however we will ensure that our data extraction framework (see *Box 1* and *Data Extraction* section) is designed to capture contextual details described in the included studies, including migration patterns and community-level characteristics. This approach will allow us to better reflect the nuances of where populations come from and why, based on empirical evidence.

C3: Line 62: I would complete this sentence with a statement like: «owing to limited hygiene and therefore highly prone to the development of infectious outbreaks.» See <https://doi.org/10.1038/s41579-021-00639-z>

Drawing on the cover letter, the authors would also like to complete this statement with another one showing data about how rapid urbanization, increase in population density and the lack of health care infrastructures and resources drive the widespread of infectious diseases (IDs) in urban areas in LMICs.

We appreciate the suggestion. However, our review is not limited to outbreaks but is designed to capture the full range of infectious disease interventions delivered interpersonally in urban poor LMIC settings as explained above. The current text already highlights how rapid urbanization, high population density, and weak infrastructure create environments where infectious diseases thrive (see Introduction, second paragraph). To keep the Introduction concise (in line with recommendations made earlier) we prefer not to expand further.

C4: Line 63: Is this an urban area of a LMIC? If need be to take such an example alongside the above-suggested revisions, I would take the example of a prototypic highly populated urban area of a LMIC with increased ID burden (e.g., an urban area of Nigeria).

We appreciate this suggestion. We have removed these lines completely to ensure our introduction is concise as earlier suggested.

C5: Line66-67: Alongside the comment I made in line 62, this sentence would better be moved when presenting data about how rapid urbanization, increase in population density and the lack of health care infrastructures and resources drive the widespread of infectious diseases (IDs) in urban areas in LMICs. Once again, I suggest revising and completing this sentence to focus on urban areas of LMICs and on consequences of this fact with regard to the spread and control of infectious diseases.

This article may be helpful in this regard; <https://doi.org/10.1038/s41579-021-00639-z>

We thank the editor for this suggestion. As noted in our response to C3, our review is not restricted to outbreaks but covers a broader set of infectious disease interventions in urban poor LMIC settings. The current placement of this sentence in the Introduction is intended to show how rapid urbanization and weak infrastructure create conditions where infectious diseases thrive, thereby motivating the need for adapted interventions. While we recognize the value of framing in terms of outbreak vulnerability, shifting the focus in this way would change the scope of the review. We therefore propose to retain the current structure, while ensuring that our extraction framework captures how interventions were adapted in response to the full range of challenges faced in urban poor settings.

C6: It would also be great to end this comment with a few details about why the

I think this comment was cut off and we are unsure how to reply.

C7: While commenting on how urbanization in LMICs drives the spread of ID outbreaks, it would be great to mention whether and eventually why the problematic of ID control in rural areas of LMICs would be less relevant than that of ID control in urban areas of LMICs.

We thank the editor for raising this point. Most infectious disease control programs operate in rural areas, usually within existing primary health care units. However, our focus on urban poor settings does not imply that rural infectious disease control is less relevant. Rather, the delivery of interventions differs substantially between urban and rural contexts. Urban areas pose distinct challenges—including density, heterogeneity, and mobility—that require different forms of adaptation than those typically needed in rural settings. To keep the review focused and feasible, we have chosen to examine urban poor contexts, while recognizing that rural delivery remains equally important but outside the scope of this protocol.

C8: Line 69: If the authors implement the revisions suggested above, they would have highlighted how rapid urbanization with increase in population density and lack of health care infrastructures and other resources in urban areas of LMICs drive and sustain the widespread of ID outbreaks in urban areas of LMICs at this level of the introduction. Accordingly, from line 69 to line 109, they would better focus on the current state of knowledge about interventions to control IDs in urban areas of LMICs in one paragraph, and on gaps in knowledge about interventions to control ID outbreaks in urban areas of LMICs in another paragraph. When presenting the current state of knowledge and gaps of knowledge, the authors would like to address issues such as whether the interventions are tailored to the context and culture of the researched populations and how much they contributed to the development and implementation of those interventions, including the role of community leaders and local researchers. While doing this, the authors

1. DOI: [10.1016/S1473-3099(24)00744-8](https://doi.org/10.1016/s1473-3099(24)00744-8)
2. <https://doi.org/10.1186/s13643-024-02651-9>

These articles would greatly help the authors frame the needed text:

<https://doi.org/10.1186/s13643-024-02651-9>

<https://doi.org/10.1136/bmjgh-2019-001855>

Bayingana et al. Lancet Glob Health 2025; 13: e1627–35.

<https://doi.org/10.1038/s41579-021-00639-z>

<https://doi.org/10.1371/journal.pone.0328645>.

<https://doi.org/10.1371/journal.pone.0317177>

We thank the editor for this helpful suggestion. We agree that the Introduction could be strengthened by distinguishing more clearly between (1) what is already known about infectious disease interventions in urban LMIC contexts, and (2) the remaining gaps in knowledge. We have thus revised our third paragraph along those lines.

C9: Line 97: This is rather a scoping review protocol.

We agree with this clarification. The manuscript is indeed a protocol for a scoping review, not a completed review. This is already indicated in the title (*“…a scoping review protocol”*) and reiterated in the *Protocol Design* section (line 120 onwards).

 In the **Abstract, Materials and Methods** we wrote:
*“This scoping review will follow Joanna Briggs Institute (JBI) guidelines and the PRISMA methodology adapted for scoping reviews (PRISMA-ScR).”*

 In the **Protocol Design section** we wrote:
*“…we will conduct a scoping review using the Joanna Briggs Institute (JBI) guidelines [24] and utilize the PRISMA extension for scoping reviews (PRISMA-ScR) guidelines for reporting [25].”*

C10: Line 111-117 (on objectives): Consider revising this text using a simple language (simple sentences). It seems to me that you would like to assess whether interventions used to control the spread of ID (outbreaks) so far in urban areas of LMICs were contextually and culturally appropriate or relevant. If this is the case, it should be clearly stated. You should also consider the rationale for conducting any scoping review (map the body of evidence on a given subject) when framing the aim of this scoping review protocol (not yet the scoping review). See <https://doi.org/10.1186/s12874-018-0611-x>

<https://doi.org/10.1371/journal.pone.0328645>

<https://doi.org/10.1371/journal.pone.0317177>

We thank the reviewer for this observation. The objective of our review is not to assess the effectiveness of interventions in outbreaks, but rather to map how interventions have been adapted for urban poor LMIC settings. This is already reflected in our research questions (lines 98–103), but we agree the aim could be stated more clearly. We will revise the objective to emphasize that our review will (1) describe the types of adaptations reported and their relation to context, and (2) examine the methods and outcomes used to study these adaptations.

C11: Apart from the aim of the review, what is its overarching aim in terms of public/global health? What specific alert different from that sent by recent reviews on a similar topic do you wish to send to the Global Health community with the upcoming scoping review

We appreciate this question. Our overarching aim is to strengthen the evidence base on delivering infectious disease interventions equitably to marginalized populations. Whereas *Epling et al.* examined racial, ethnic, and religious marginalization, globally this protocol focuses on urban poor settings in LMICs, where marginalization is shaped by socioeconomic and environmental conditions. By mapping adaptations in this distinct context, our review complements earlier work and extends understanding of how delivery strategies must adapt to different populations.

We have summarized and added these lines into our manuscript.

C12: Line 121-126: I suggest moving this statement to lines 113 to 117 where you highlight the aim of the upcoming scoping review, along with my comment above.

We agree and have moved the objectives into a dedicated subsection to improve clarity and alignment with PLOS ONE formatting.

C13: Line 125-126: I understand that PLOS One recommends the authors upload the PRISMA-P checklist when submitting a scoping review protocol. However, I would like to inform the authors that the PRISMA-P checklist provided as S1 is for systematic review and meta-analysis protocols whereas this is a scoping review protocol. Given the use of the JBI and PRISMA-Scr guidelines for the upcoming scoping review, I suggest also using either of both guidelines for reporting this scoping review protocol.

Thank you for catching this. We have replaced the checklist with the PRISMA-ScR version.

C14: Line 126: I understand a scoping review protocol article may serve as registration record of a scoping review (https://www.icmje.org/recommendations/). However, did the authors register this protocol article or consider registering it? See <https://doi.org/10.1371/journal.pone.0328645>

<https://doi.org/10.1371/journal.pone.0317177>

We thank the reviewer for this suggestion. In addition to publishing in PLOS ONE, we have registered the protocol on the Open Science Framework (OSF) to ensure transparency and accessibility, and have added the registration link to the Methods section.

C15: Line 128: I suggest deleting this sub-title.

We agree and have removed this sub-title to simplify the structure and improve flow.

C16: Line 129-130: This statement belongs to the first sub-section (Protocol design) of the Materials and methods section above.

We agree. We have moved this statement into the *Protocol Design* subsection to improve clarity and structure.

C17: Line 131: This sub-section should be termed “Eligibility criteria” and clearly describe inclusion and exclusion criteria in separate paragraphs.

We agree, and we have now included a section on eligibility criteria with most of the information presented in table format per your comment below.

C18: Line 135: Consider this sub-section after the Eligibility criteria sub-section.

We agree and have moved this subsection so that it follows directly after the *Eligibility criteria*. This reordering will improve the logical flow of the Methods section.

C18: Line 136: Consider replacing this statement with something like «onwards».

We have replaced the statement with onwards.

C19: Line 140: I insist is it necessary to mention the word «poor», stereotypic term which can contribute to the maintenance of social class inequalities?

See <https://doi.org/10.1016/j.copsyc.2017.07.033>

<https://doi.org/10.1093/hrlr/ngab016>

Please see response above, we have changed to use “low-income urban communities”

C20: Line 140-142: You need to provide refs here.

We have provided 3 references here

C21: Line 141-142: I understand there is no universal definition for spatial concepts or a definition specific to the so-called LMICs. However, you may consider proposing a new definition of spatial concepts based on knowledge from another group of countries such as European countries, especi ally since references 17 and 28 used to support the use of these terms (see line 142) do not seem to support the use of these search terms to refer to the term «urban». Accordingly, you could rather consider this literature, especially since there are also so-called LMICs in Europe: <https://ec.europa.eu/regional_policy/sources/work/2014_01_new_urban.pdf> or that from your country.

We thank the reviewer for this comment and their opinion. However, we decide to cite other articles that have used these terms in recent reviews.

C22: Line 143-151: Following the comment made just above, is this citation gaming? <https://doi.org/10.1177/05390184221142218>

We are unable to respond to this comment, because the lines cited do not have any citations.

C23: Line 143-151: Can you revise the preliminary search strategies presented in the S2 file and frame this sub-section as did these authors? <https://doi.org/10.1371/journal.pone.0328645>

We have revised our search strategies to conform to the PRISMA-Search guidelines, which specify to "Include the search strategies for each database and information source, copied and pasted exactly as run". We have also framed the sub-section as much as we can to the style of the article shared.

Rethlefsen, M.L., Kirtley, S., Waffenschmidt, S. et al. PRISMA-S: an extension to the PRISMA Statement for Reporting Literature Searches in Systematic Reviews. Syst Rev 10, 39 (2021). https://doi.org/10.1186/s13643-020-01542-z

C24: Conform to search strategy guidelines: <http://dx.doi.org/10.1016/j.jclinepi.2016.01.021>

DOI: dx.doi.org/10.5195/jmla.2018.283

Given that the key search concepts should emanate from the aim of the review, would you not consider these four terms as the key search concepts: ID, intervention, urban and LMICs and then develop each of those terms in the search strategies? Obviously, the LMICs mentioned in search strategies should be those of the LMIC classification chosen as highlighted when commenting on Table 1?

Thank you, we are unsure how to interpret this comment as it pertains to the provided DOIs: the first article is a guideline about peer reviewing search strategies which is recommended and not required for scoping reviews. The second article provides recommendations for searching but no requirements or guidelines. However, following your comment 23 above, we have updated the presentation of our search tables to conform to the PRISMA-S extension guidelines (see C23 above, and our S2 File).

Our PCC Framework also corresponds exactly with our search strategy: low-income urban communities (Search 2), infectious disease interventions (Search 1), and LMICs (Search 3).

C25: Line 152-164: As commented above, the sub-title should be revised to “Eligibility criteria” and the sub-section should be moved to line 128

We have made these changes as requested (See C17)

C26: Line 153-164: You get to choose whether you detail the inclusion and exclusion criteria in the text or in a table, not in both formats. Given that the table presentation appears to be more complete than the text, I would choose the table format.

Thank you for the guidance. We have summarized the inclusion and exclusion criteria description in the text to the barest minimum, and made reference to the table which is more detailed.

C27: Table 1: Have you provided all the socioeconomic and demographic characteristics of the population group to be included in the scoping review? I mean age limit, sex and gender, race, ethnicity, nationality, geographic origin, economic status

Beyond specifying that studies must focus on populations living in **urban poor settings in LMICs**, we have not imposed additional restrictions by age, sex, gender, race, ethnicity, or nationality. This is consistent with the exploratory nature of a scoping review and will allow us to capture the widest possible range of evidence. Where such characteristics are reported in the included studies, we will extract them as part of the data charting process (see *Data Extraction* section and Box 1).

C28: Table 1 (on concept): Did you wish to write “Concept” here?

We thank the reviewer for catching this error. Yes, this was a mistake, and we will correct the heading in Table 1 to read “Concept.”

C29: Could you provide more details here about the type of (mass/population-based/public health as opposed to individual interventions which seem to be out of the scope of the upcoming review) interventions classified as preventive interventions, diagnostic interventions, and curative interventions? This means you would need three arrows for the variable “Intervention”. For each type of intervention, you would then study the intervention modalities, how they were evaluated and how they were adapted to the context and the culture.

Thank you for this helpful comment. Our scope is population-level interventions that require interpersonal delivery. We agree it will be clearer if we explicitly subdivide “Intervention” into preventive, diagnostic, and curative categories. We will also extract (i) delivery modality, (ii) adaptation to context, and (iii) study methods.

C30: Table 1 (on evaluation): Do you wish to only assess how interventions were adapted and evaluated without studying the interventions themselves? Because it does not seem that you wish to do so when going through this table.

We apologize for the confusion. We have deleted this row.

C31: Table 1: (on outcome): Outcome of intervention is a variable that should be distinct from the intervention variable and so, be addressed in another arrow of the concept part of the PCC concept.

We apologize for the confusion regarding the formatting of the table. To clarify, **intervention, information on adaptation, and reported outcomes are all distinct elements within the “Concept” domain**. In the revised Table 1, we present them as separate rows under the broader PCC category of “Concept”.

C32: Could you subdivide the outcome of intervention variable based on timeline (short-term, mid-term and long-term) and presence of absence of post-ID survival (survival outcomes versus hard outcomes [including quality of life, psychosocial well-being…)?

Please allow us to clarify, **Table 1 presents our eligibility criteria**, not our data extraction framework. For inclusion, studies must report at least one outcome, since our focus is on identifying interventions that have been delivered and adapted in practice. We will revise Table 1 to make this clearer.

C33: Table 1: on context: How will you ensure the readers believe that the countries included in the review are indeed LMICs? In other words, LMICs according to the World Bank or other classifiers? See BMJ Glob Health. 2022 Jun;7(6):e009704. doi: 10.1136/bmjgh-2022-009704. High-income countries based on which classification?

When finalizing the eligibility criteria and search strategy, we referred to The World Bank Classifications to determine which LMICs should be included. (please see ref 23). We have revised the text under objectives to reflect this.

World Bank. World Bank Country and Lending Groups. [cited March 25, 2025]. In: Knowledge Base > Country Classification [Internet]. Available from: https://datahelpdesk.worldbank.org/knowledgebase/articles/906519-world-bank-country-and-lending-groups

C34: Table 1: (under context): Do you make a difference between the arrow on countries and this one or did you wish to write “Study setting” rather than “Residence”?

We thank the reviewer for this comment. We have now revised this as Study Settings.

C35: Still regarding the setting, did you talk about the locality in a given country? Urban versus rural vs semi-urban? Bayingana et al. Lancet Glob Health 2025; 13: e1627–35. How will you define an urb an area? See for example how they defined an urb an are a in a so-called LMIC: <https://doi.org/10.1016/j.jrurstud.2020.08.014>.

We agree this is an important point. As noted in the Introduction (and in the reference you cite above), there is no universal definition of “urban.” In our Methods (search strategy) we already specify that a wide range of terms were used (e.g., “slum,” “informal settlement,” “street dwellers”). To make this clearer, we have revised the Context description in Table 1/Eligibility criteria to state explicitly that studies were included if authors described the setting as urban using any of these terms.

C36: Table 1: Type of study: Did you wish to say: “Study design” and “Type of publication”? Consider reframing this box to make it readable. For instance, can a mixed-methods or a qualitative study be a quasi-experimental study? What is RCT? What is the “Case studies” study design? The authors also need to reflect on the study designs most likely to address the preventive, diagnostic and curative interventions they wish to assess when framing the text of this box.

Thank you for the comments, we agree. We meant Study design and Type of publication. We have revised the text to read: Studies that are peer-reviewed and published. Can be qualitative, quantitative or mixed methods, and also descriptive or analytic.

C37: Type of publication and study design should be addressed in different arrows of the context part of the PCC concept.

Please see comments C36, AND our revised table on how we have framed it

C38: Table 1 (on type of study): Why do you exclude these types of publication records/ is it not possible to include information from reviews, opinions, textbooks (e.g., chapters) and grey literature in a scoping review which ethymologically aims to map the body of evidence on a given subject? <https://doi.org/10.1186/s12874-018-0611-x>

We appreciate the reviewer’s question and agree that scoping reviews can, in principle, include a broad range of evidence sources, including reviews, opinion pieces, textbooks, and grey literature. However, the aim of our review is more narrowly defined: to map how **primary research studies** have described the implementation and adaptation of infectious disease interventions in low-income urban communities. We also note that grey literature and non-empirical sources vary widely in quality and reporting detail, and a full appraisal of these materials is beyond the scope and resources of this review. We therefore restrict inclusion to original research articles that report empirical findings on interventions in these settings.

That said, we will consult relevant reviews and grey literature to ensure our search strategy is comprehensive and to contextualize findings, but they will not be included in the data charting or synthesis.

C39: Table 1 (on study subject): Consider deleting this arrow because the studied population is mentioned in the first arrow of this table and that is therefore the place where to specify that the review will focus on human subjects rather than animal subjects.

We agree, and have deleted the row

C39: Table 1: (on year of publication): Consider deleting this arrow. The information provided here belongs to “Information sources and search strategy” sub-section.

We agree, and have deleted the row

C40: Table 1: (language): Did you wish to specify “Publication language”?

Yes, thank you for catching this error. We have updated the manuscript.

C41: Line 168 (article screening): I am glad you will do this. Given that all authors are affiliated with institutions located in the USA where English language is the only official language, how are you going to ensure the accuracy of the information retrieved from publications in languages other than English? Consider specifying.

Our research team includes Portuguese, Arabic, French, and Spanish speakers and we have a wide network of colleagues that we can reach out to with other languages should they arise.

We recognize that positionality - including our own location and identities - can introduce risks of Anglocentric or Western lens bias, especially in screening and interpreting studies. It is therefore worth noting that our first author (MHO) is Nigerian living in the US, our last and corresponding author (MCB) was brought up in Peru and worked embedded in Ministries of Health in the S Pacific and Latin America, and a third author (IF) has lived experience in East Africa. This brings diversity of perspective to our study.

C42: Line 168: Consider renaming this sub-section «Study selection». Cn you make the text of this sub-section more understandable in a simpler language as in already published scoping review protocols? See <https://doi.org/10.1371/journal.pone.0328645>

<https://doi.org/10.1371/journal.pone.0317177>

C43: Line 171 (about pilot screenings): This sentence is difficult to understand for me

We have revised the text for better clarity

C44: Line 172 (about authors): Can you provide the full-spellings of these abbreviations

Those abbreviations are the author initials. However, we have removed them.

C45: Line 180: Why a PRISMA flow diagram? Will the review be a systematic or scoping one?

This is a scoping review, which also uses a PRISMA diagram - per JBI guidelines.

C46: Line 186-189: Could you better specify the variables (variables about study characteristics, variables about participant characteristics, and main review variables including the intervention variable [intervention, adaptation and evaluation of the intervention] and the outcome of intervention variable) whose data will be considered when extracting data from primary studies?

These are described in box 1. Specific nature of what is collected will depend on what is included in the published articles being reviewed.

C47: Line 191-192: Can you explain in more details how the ERIC and PRISM recommendations informed the conception of the data extraction process for the intervention variable and the outcome of intervention variable for this protocol article? I ask this question because apart from the fact that the upcoming review should be different from recent reviews on a similar topic, ERIC recommendations were made by experts from USA and Canada, but not by experts located in so-called LMICs. I do not know whether LMIC experts were involved in the recommendations recorded in ref 30 came from because I am un able to access its full-text. However, it seems to me that those recommendations would be useful mainly to synthesize data on the implementation of interventions and the evaluation of those interventions. It is difficult for me to visualize how ref 29 will be helpful here when going through it because it seems to be helpful for assessing the feasibility and relevance of public health interventions, but I missed in the text above information about whether you are willing to assess whether those interventions were feasible before their implementation and relevant, or at least it was not so clearly stated in the introduction and preceding sub-sections of this section of the manuscript.

We have clarified above how the topic of this review is different to what has been published.

As the aim of our paper is to describe *how* interventions were adapted, it is important for us to capture in detail the intervention descriptions in the articles that we review. In this protocol, we have drawn on both the **ERIC (Expert Recommendations for Implementing Change)** and **PRISM (Practical, Robust Implementation and Sustainability Model)** frameworks to guide the design of our data extraction.

We used ERIC as a reference to ensure our extraction captures not only *what the intervention was* but also *how it was implemented and adapted* to local context. For example, in Box 1 we include extraction items such as how communities were approached, which organizations led delivery, what coordination mechanisms were used, and what facilitators/barriers were described.

PRISM similarly has broad uptake in implementation science and guided our attention to *contextual and outcome variables*. It emphasizes how interventions interact with organizational, community, and system-level factors, and how these shape sustainability. Accordingly, our extraction of outcomes goes beyond effectiveness to include implementation outcomes (coverage, uptake, fidelity), sustainability outcomes (duration, maintenance of activities), and experiential outcomes (acceptability, perceptions).

C48: Box 1: I suggest renaming this title: «Information to be extracted for the interventions and outcome of interventions variables»

Thank you for this. We have revised the title to - Information to be extracted about the interventions.

C49: Box 1: Drawing on the box title proposed above, I suggest revising this box so that the information about the interventions variable (type implemented [preventive, diagnostic and curative], implemented interventions for each type, how each intervention was adapted, and how each intervention was evaluated) and about the outcome of interventions variable are presented separately and clearly.

We have revised the box accordingly to include the intervention type, delivery modalities, and to note that outcomes measured will be recorded.

C50: If this box was built based on information from reference 30, did you get permission to modify their figure/table? Even if you did not modify one of its figure/table, you would better specify in the footnote of this box that you built it based on reference 30.

It was informed by ERIC and PRISMA frameworks and then applied to this study, it is not a modification of a table or figure in those studies.

C51: Box 1 (effectiveness): To my knowledge, these are not bits of information about outcomes of interventions, but rather about the cost-effectiveness of those interventions. I am glad you would potentially like to include information about the cost-effectiveness of those interventions because this information would

We have revised the sub-head to read outcomes and measures

C52: Line 200-205 (analysis of results): Consider thoroughly revising this sub-section, specifying how the ERIC and PRISM guidelines would eventually help synthesize the upcoming scoping review. How will data be presented? Will you stratify any data? If yes, which ones and by which variable (e.g., world region)? In brief, see how other authors framed this sub-section: <https://doi.org/10.1371/journal.pone.0322753>

ERIC and PRISM were used to inform the design of the extraction framework only. We have thoroughly revised this section. Please see below

We have added a sub-section to the methods section describing in detail how we will analyze the data,.

C53: Line 207: Consider removing this sub-title.

We have removed the title and the section completely

C54: Line 208-212: This text belongs to the Discussion section which should be inserted after the Materials and Methods section, and should be further elaborated, describing the strengths and pitfalls of the methodology conceived for the upcoming scoping review. The authors would like to go through the previously provided scoping review protocol articles for inspiration on how to frame the discussion section.

We have removed the title and the section completely

C55: Line 214-221: Is this realistic? When are the implementation of the se arch strategy and the elimination of duplicates due to take place? Is this timeframe realistic?

We believe our timeframe is realistic. We were able to start the screening process based on feedback received in this review and are on track.

C56: Line 221: I suggest adding the sub-sections protocol deviation, data sharing and Ethical considerations, and Patient and public involvement at the end of the Materials and Methods section. See how the others did that:

<https://doi.org/10.1371/journal.pone.0322753>

<https://doi.org/10.1371/journal.pone.0328645>

These sections has been added now

C57: Line 224: Before this statement and just after the Discussion section, consider adding a «Dissemination» statement as did these authors: <https://doi.org/10.1371/journal.pone.0328645>

This section has been added

C58: References: Strengthening and update will increase the relevance of this protocol.

We have updated the references.

**Reviewer 1:**

C1: Abstract needs minor improvements; “Materials and methods” section is slightly dense; simplifying sentences (e.g., breaking long lists of steps) would improve readability.

Thanks for these comments. We have significantly revised the abstract and methods

C2: The conclusion could be more specific by emphasizing the expected contribution of the scoping review (e.g., identifying adaptation strategies, informing future program design).

Thanks for these comments. We have now revised the conclusion as suggested

C3: Consider consistency in terminology: “urban poor setting” could be pluralized to “urban poor settings” to match the rest of the manuscript.

Following revisions proposed by the editor, we have replaced the term “urban poor” with “low income urban communities” through the text

C4: Some sentences are long and could be simplified for clarity. For example, the sentence beginning line 60 “The rapid pace and scale of urbanization…” could be broken into two.

Thank you – we have further edited the text.

C5: Minor typographical issues: “leisMHOaniasis” line 80 appears to be a typo.

Thank you for catching this - we have revised it.

C6: The aim and objectives are currently somewhat broad and overlapping with background information, which makes them less clear. The aim uses vague language (“to understand what has been published”) and would benefit from being more action-oriented, for example, “to map and synthesize evidence on adaptations of infectious disease interventions in urban poor settings in LMICs.” The objectives should flow directly from the aim and clearly reflect the research questions, specifying the population, setting, and type of interventions studied. Reframing the objectives to explicitly address (1) intervention adaptations in relation to context and (2) study designs, methods, and outcomes would improve clarity, specificity, and alignment with the research questions

Thank you for these comments. We have carefully reworded the aim, and objective of the review. Please see our new section on "Aims and objective of the scoping review”. This has helped clarify our own thinking.

C7: Some sentences are long and could be simplified for clarity, particularly in the “Information sources and search strategy” and “Data Extraction” sections. Breaking these into shorter sentences would improve readability.

Thank you – we have further edited the text.

C8: The use of the PCC (Population, Concept, Context) framework is suitable, but the description could be slightly more concise. For example, the “concept” could clearly state it focuses on interpersonal delivery adaptations rather than listing examples at this stage.

We have revised this section to ensure clarity. Please see our section on “Protocol design”.

C9: The rationale for selecting 2000 as the start year is not explicitly stated; adding a brief justification would be helpful.

We selected 2000 as the start year to ensure the review captures evidence from a time frame that is both **relevant to current global health delivery models** and **feasible for review**. Many large-scale infectious disease control initiatives in LMICs—including global immunization campaigns, scale-up of HIV/TB programs, and mass drug administration for NTDs—expanded significantly after 2000, often with increased attention to delivery in urban contexts.

We have now added this line under our inclusion criteria.

C10: Consider providing more detail on how search results will be managed across multiple languages, especially for non-English studies.

Thank you for the opportunity to clarify. As noted, we will not exclude studies based on language at this stage. If we have language capacity on our team, studies will be read in the original language. Where this is not possible, we will use translation software (e.g., Google Translate) to produce an English version and ask someone proficient in the language to compare the translation with the original to confirm accuracy. If this is not possible, we will document the exclusion of the study and report it transparently in the review.

C11: The table is detailed and clear. However, there is a minor inconsistency: in the text, the authors state that grey literature will be included, page 9, line 160, but in the table, grey literature is listed under exclusion. Clarification is needed.

Thank you for this observation. We have corrected line 160, as grey literature will not be included.

C12: The criteria could also briefly explain how mixed interventions or multi-component studies will be handled.

In mixed interventions or multi-component study information on each intervention and component will be collected.We have now added this text under our Analysis section

C13: Box 1 is detailed but long; consider summarizing key elements in the main text and moving detailed subpoints to an appendix.

We have revised this box also following comments from the editor to add more detail and would prefer to retain it within the main file. We believe pulling out the detail of interventions and how they were implemented is critical in implementation science.

C14: Clarify whether any software or tools will be used to manage qualitative synthesis (e.g., NVivo, MAXQDA) or if this will be entirely manual.

Thanks for this comment. Qualitative synthesis will be managed manually and supported by AI. We have added more detail on how this will be done to the sub-section on analysis under results.

C15; The timeline is realistic, but adding anticipated challenges or mitigation strategies (e.g., delays in full-text retrieval or translation) could strengthen planning transparency.

We appreciate this thought. However we have been able to start the screening, implementing feedback received in this review, and we believe we are on track. If our timings change we will report.

C16; Some citations are repeated or appear as placeholders (e.g., “[17, 17,28]”); these should be checked for accuracy, line 142.

We have also revised this. Thanks for catching this error.

C17: One minor point: the funding statement contains a typographical error (“No was received” should likely read “No funding was received”). Otherwise, this section is clear and adequate.

Thanks for catching this error. We have now revised it

C18: Minor language, typographical, and consistency issues should be corrected.

We have carefully revised the manuscript and corrected typographical errors.

C19: Emphasizing action-oriented language in the objective will strengthen the manuscript’s clarity and alignment with scoping review methodology.

Thank you, we have revised this and think it is now stronger.

C20: Please check your references and be consistent

We have carefully revised the manuscript and addressed this.

C21: Line 97: The aim and objectives are currently written in a way that is somewhat wordy and overlaps with background information. Ideally, the aim should be a clear, concise statement of the overall purpose of the review, separate from the context

We have carefully revised the manuscript and addressed this.

C22: Line 97; The aim uses the phrase “to understand what has been published,” which is vague and passive. For a scoping review, it is better to use action-oriented language such as “to map,” “to synthesize,” or “to describe.”

We have carefully revised the manuscript and addressed this. Please see our comments above on this.

C23: Line 113: The objectives should flow naturally from the aim and clearly reflect the research questions. Currently, there is a slight disconnect: the aim emphasizes understanding what has been published, whereas the objectives focus on intervention adaptations, contextual factors, methods, and outcomes. Please align the objectives to your research question

We have carefully revised the manuscript and addressed this. Please see our comments above on this.

C24: Line 114: The objectives could be more specific regarding the population (urban poor), setting (LMICs), and type of interventions (interpersonal delivery-focused interventions). This specificity will make the aim and objectives sharper and more actionable. Be clear with your scope

Thank you. We have carefully revised the manuscript and addressed this.

C25; Line 114: Why re you writing research questions mixed with research objectives? Reframe the aim to a concise, action-oriented statement. Reframe the objectives to explicitly reflect the research questions.

We have carefully revised the manuscript and addressed this.

C26: Line 166. Table 1: This PCC table is too broad, make it concise

We have carefully revised the manuscript and addressed this.
